# An Update on the Synthesis of Pyrrolo[1,4]benzodiazepines

**DOI:** 10.3390/molecules21020154

**Published:** 2016-01-28

**Authors:** George Varvounis

**Affiliations:** Department of Chemistry, Section of Organic Chemistry and Biochemistry, University of Ioannina, 451 10 Ioannina, Greece; gvarvoun@cc.uoi.gr or gvarvoun@gmail.com; Tel.: +30-26510-08382; Fax: +30-26510-08799

**Keywords:** small molecules, heterocycles, pyrrolobenzodiazepines, DNA-interactive agents, anticancer activity, anti-HIV activity

## Abstract

Pyrrolo[1,4]benzodiazepines are tricyclic compounds that are considered “privileged structures” since they possess a wide range of biological activities. The first encounter with these molecules was the isolation of anthramycin from cultures of *Streptomyces*, followed by determination of the X-ray crystal structure of the molecule and a study of its interaction with DNA. This opened up an intensive synthetic and biological study of the pyrrolo[2,1-*c*][1,4]benzodiazepines that has culminated in the development of the dimer SJG-136, at present in Phase II clinical trials. The synthetic efforts have brought to light some new synthetic methodology, while the contemporary work is focused on building trimeric pyrrolo[2,1-*c*][1,4]benzodiazepines linked together by various heterocyclic and aliphatic chains. It is the broad spectrum of biological activities of pyrrolo[1,2-*a*][1,4]benzodiazepines that has maintained the interest of researchers to date whereas several derivatives of the even less studied pyrrolo[1,2-*d*][1,4]benzodiazepines were found to be potent non-nucleoside HIV-1 reverse transcriptase inhibitors. The present review is an update on the synthesis of pyrrolo[2,1-*c*][1,4]benzodiazepines since the last major review of 2011, while the overview of the synthesis of the other two tricyclic isomers is comprehensive.

## 1. Introduction

**Figure 1:**
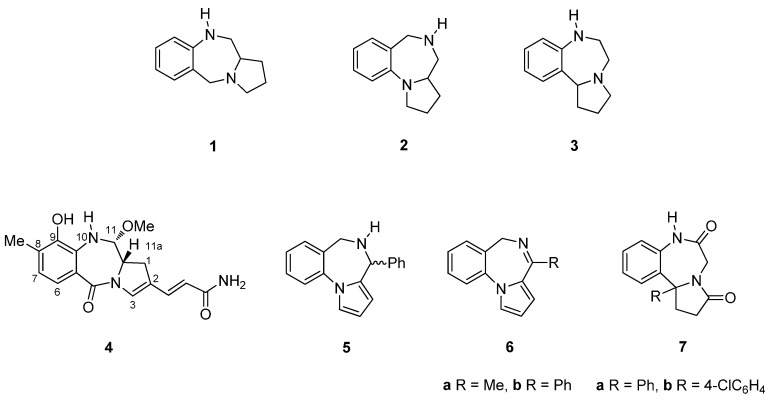
Structure of the three pyrrolo[1,4]benzodiazepine isomers **1**–**3** and the first derivatives **4**–**7**.

The pyrrolo[1,4]benzodiazepine (PBD) tricyclic ring system is represented by three possible structural isomers [2,1-*c*][1,4] **1**, [1,2-*a*][1,4] **2** and [1,2-*d*][1,4] **3** (Figure 1). The first derivative of any of these ring systems to be synthesized was a pyrrolo[2,1-*c*][1,4]benzodiazepine, anthramycin (**4**), reported by Leimgruber *et al.*, in 1968 [1]. Three years later Cheeseman and Rafiq [2] published the synthesis of the first two pyrrolo[1,2-*a*][1,4]benzodiazepines **5** and **6a**,**b**. Then in 1977, Yamawaki *et al.* [3] described the preparation of the first two pyrrolo[1,2-*d*][1,4]benzodiazepines **7a**,**b**. For the purposes of the present review, synthetic chemistry publications from the primary literature were retrieved from the SciFinder and Scopus databases. For the period 2010 to the middle of 2015, thirty-two pyrrolo[2,1-*c*][1,4]benzodiazepine-related publications were retrieved, whereas for the period 1960 to the present, twenty-five pyrrolo[1,2-*a*][1,4]benzodiazepine and seven pyrrolo[1,2-*d*][1,4]benzodiazepine publications were downloaded. This is the first review on the synthesis of PBDs that includes all three structural isomers.

### 1.1. Pyrrolo[2,1-c][1,4]benzodiazepines

**Figure 2:**
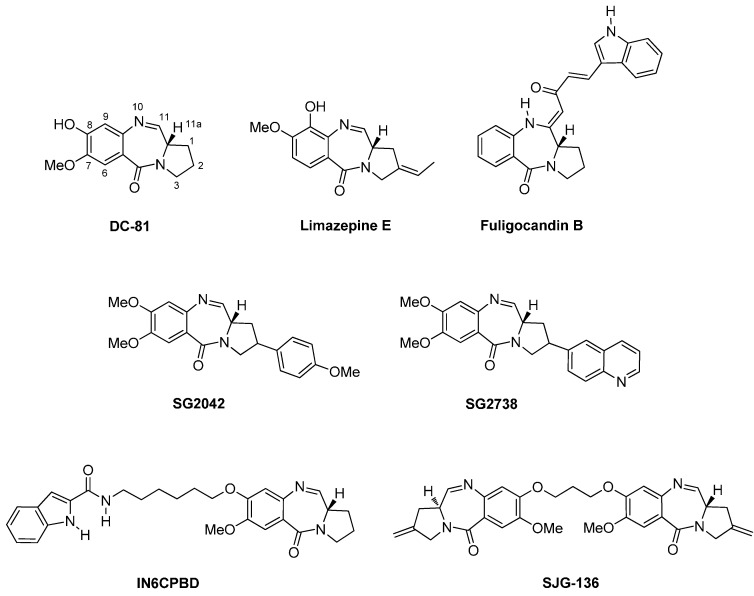
DC-81, Limazepine E and Fuligocandin B are natural products, SG2042 and SG2738 are PBD monomers, IN6CPBD is a PBD conjugate and SJG-136 is a PBD dimer.

Since the isolation of anthramycin (**4**, Figure 1) from cultures of *Streptomyces* by Leimgruber *et al.*, in 1965 [4], the chemistry and biology of pyrrolo[2,1-*c*][1,4]benzodiazepines took the lead as the most intensively studied isomer of the three. Other important PBDs isolated from *Streptomyces* species are sibiromycin, tomaymycin, the neothramycins and DC-81 [5]. Their mode of action relies on the right-handed helical conformation due to the (*S*)-configuration at C-11a, found from an X-ray crystal structure analysis of anthramycin (**4**) [6], enabling the molecules to adapt in the minor groove of DNA, selectivity at the 5′-purine-G-purine sequences, by alkylating the C2 amino group of guanine [7]. Anthramycin was the most promising of these natural products having activity against gastrointestinal and breast cancers, lymphomas and sarcomas. Although possessing low haematological toxicity, clinical use is limited because of dose-limiting cardiotoxicity. More recently limazepine E was isolated from a culture broth of *Micrococcus* [8] and fuligocandin B from the myxomycete *Fuligo candida* [9] (Figure 2).

The unwanted side-effects of the naturally occurring PBDs led to prolific research on the synthesis of analogues, known as PBD monomers. Two notable examples that have been tested *in vivo* against various human tumour xenografts are SG2042 and SG2738 possessing the C10-C11 imine structure (Figure 2), but at the end of the line no critical DNA damage was observed. Moreover, structure-activity relationship (SAR) studies have shown that linking primarily position C8 of DC-81 with other moieties such as other DNA intercalators or pyrrole and imidazole polyamide analogues of dystamycin and neotropsin, would produce PBD conjugates possessing two pharmacophoric heads with improved *in vitro* DNA binding affinity, sequence selectivity and/or cytotoxic efficacy. One promising outcome of this research was the work of Wang and co-workers [10] who designed and synthesized the indole-PBD conjugate IN6CPBD (Figure 2) and found that this compound exhibited higher cytotoxicity than DC-81 against human melanoma A375 cells and displayed enhanced DNA sequence selectivity. A significant breakthrough in the search of PBD antitumour agents was the discovery of PBD dimers, designed to be capable of creating cross-links in the DNA by forming covalent bonds to guanine bases at each end of the molecule. This work culminated in the development of C2-*exo*-methylene PBD dimer SJG-136 (Figure 2), at present in Phase II clinical trials.

The extensive literature on the isolation of PBD natural products and the synthesis of PBD monomers has been reviewed first by Thurston and Bose in 1994 [11]. An update including PBD conjugates and PBD dimers was reported recently by Antonow and Thurston [12]. In the present review the synthetic chemistry literature from 2010 to mid-2015 is covered.

Recently, a review by Hartley [13] described the development of PBD as antitumour agents while a review by Gerratana [14] reported the biosynthesis, synthesis, and biological activities of PBDs. The synthetic chemistry section of the latter article does not provide coverage of the work published since the last review [12]. The evaluation of the antitumour activity of PBDs is dominated by *in vitro* cytotoxicity against tumour cell lines, and, DNA thermal denaturation experiments and DNA footprinting assays, used to measure the DNA-binding affinity and find the sequence selectivity of these compounds.

The publications [15,16,17,18,19,20,21,22,23,24,25,26,27,28,29,30,31,32,33,34,35], cover the period 2010 to the middle of 2015, and refer solely to biological studies of PBDs. Three publications [36,37,38] are devoted to spectroscopic studies on PBDs whereas one publication deals with computational studies of PBDs [39] and another with the isolation of natural PBDs [40].

### 1.2. Pyrrolo[1,2-a][1,4]benzodiazepines

The comparatively small number of publications on the [1,2-*a*] and [1,2-*d*] isomers is a reflection of the lesser interest in the biology of these compounds. Nevertheless, it is known that pyrrolo[1,2-*a*][1,4]benzodiazepines exhibit a wide spectrum of biological effects, such as antinociceptive CNS [41,42], anti-inflammatory [41], analgesic [43] and fungicidal [44] activity, and are potent sedative [41,45,46], anticonvulsant [45,46], myorelaxant [45,47] and psychotropic [43,47] agents.

### 1.3. Pyrrolo[1,2-d][1,4]benzodiazepines

**Figure 3:**
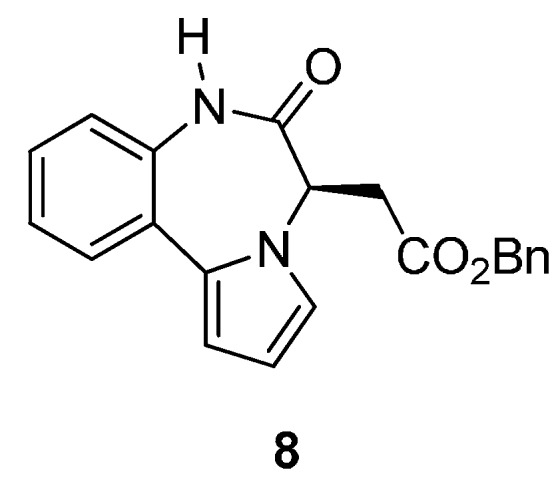
A biologically active pyrrolo[1,2-*d*][1,4]benzodiazepine.

Among the small number of publications on the pyrrolo[1,2-*d*][1,4]benzodiazepine ring system, one reference has been made to PBD **8** (Figure 3), as an important non-nucleoside HIV-1 reverse transcriptase inhibitor with IC_90_ = 0.29 μg/mL [48].

### 1.4. General Information

The review presents the synthetic routes to PBDs from simple starting materials according to the cyclisation procedure taking place to produce the PBD and also by the type of PBD synthesized.

## 2. Synthesis of Pyrrolo[1,4]benzodiazepines

### 2.1. Pyrrolo[2,1-c][1,4]benzodiazepines

The most common features of the pyrrolo[2,1-*c*][1,4]benzodiazepines presented in this review are: (i) an aromatic or non-aromatic pyrrole ring; (ii) one carbonyl group on the diazepine ring that is at position 5 of the PBD, then N10 could be a secondary amine, substituted tertiary amine or a N10-C11 imine; (iii) two carbonyl groups on the diazepine ring that are at positions 5 and 11 on the PBD, then these compounds are considered “dilactams” and (iv) monomers with various medium sized substituents, monomers that possess an extended C8-*O*-linked chain whose structure can possess various combinations of alkyl, ether, amide, carboxylic ester, carbodithioic ester, α,β-unsaturated carbonyl, carbocyclic and heterocyclic substitution (these as named “PBD conjugates”) and dimers that are linked via the *O*-atom at their C8 positions where linkers can be either a linear alkyl chain or a combination of alkyl and carbocyclic entities. C7-linked PBD conjugates and PBD trimers have not been reported in the past five years.

The most important contributions towards the chemistry and biological activity of pyrrolo[2,1-*c*][1,4]benzodiazepines have been from Antonow, Bose, Hurley, Kamal and Thurston, among which the last two authors have published the largest number of scientific papers in this field of research.

#### 2.1.1. Pyrrolo[2,1-*c*][1,4]benzodiazepine-5-one Monomers or Dimers with a Non-Aromatic Pyrrole Ring

During the last five years, three novel [2,1-*c*] PBD syntheses have been reported in this category of compounds, (Section “Reductive cyclisation of *N*-(2-azidobenzoyl)pyrrolidine-2-carboxaldehydes”), (Section “Cyclisation of *N*-(2-azidobenzoyl)pyrrolidine-2-carboxaldehydes”), although, a large number of new compounds containing [2,1-*c*] PBD scaffolds in their structure have been synthesized in the search of new effective anticancer agents. These include PDB conjugates and PDB monomers that possess an extended C8–*O*-linked pharmacophore, the most used strategy.

In this sense, the established cyclisation of *N*-(2-aminobenzoyl)pyrrolidine-2-carboxaldehyde diethyl thioacetals, described first by the Kamal group [49], is by far the most frequently used method for synthesizing these compounds. Several groups such as alkyl, carbonyl and thioacetal and ring structures including estradiol, chalcone, anisole, naphthyl, piperazine, 1,2,3-triazole, carbazole, benzo[*c*,*d*]indole, naphtha[1,8-*c*,*d*]isothiazole, bisindole, imidazo[1,2-*a*]pyridine and 1,3-benzothiazole, have been chosen in various combinations to form the second pharmacophore, that is connected to the C8–O of the PBD nucleus by the synthetic methodology presented in the following section. 

##### Cyclocondensation of *N*-(2-Aminobenzoyl)pyrrolidine-2-Carboxaldehyde Diethyl Thioacetals

All of the PBDs produced from the reactions of this subsection have an N10–C11 imine functionality in their structures. The imine group is known to interact electrophilically with the C2–NH_2_ group of a guanine within the minor groove of DNA and therefore there is a strong interest to synthesize analogues of these PBDs in order to study this interaction.

**Scheme 1:**
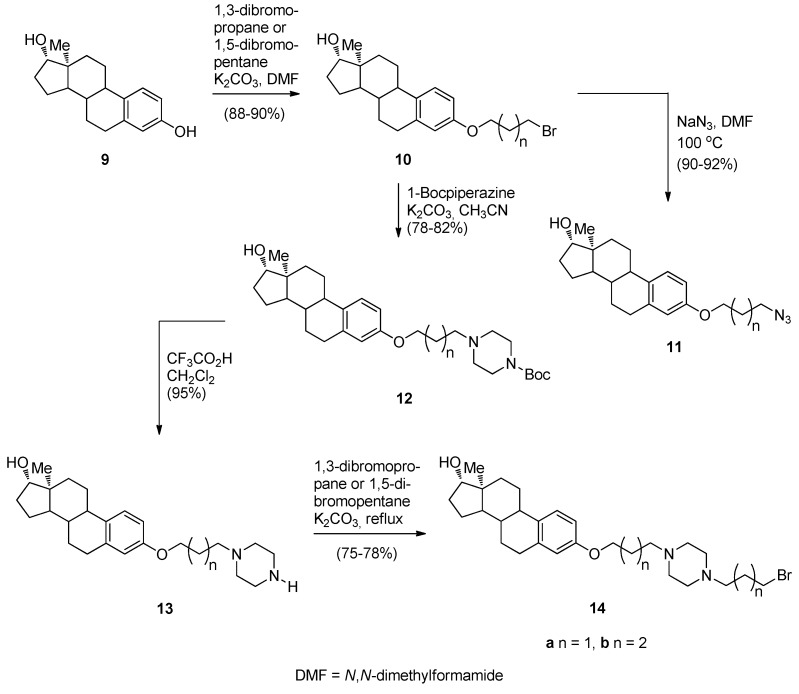
Synthesis of estradiol derivatives.

**Scheme 2:**
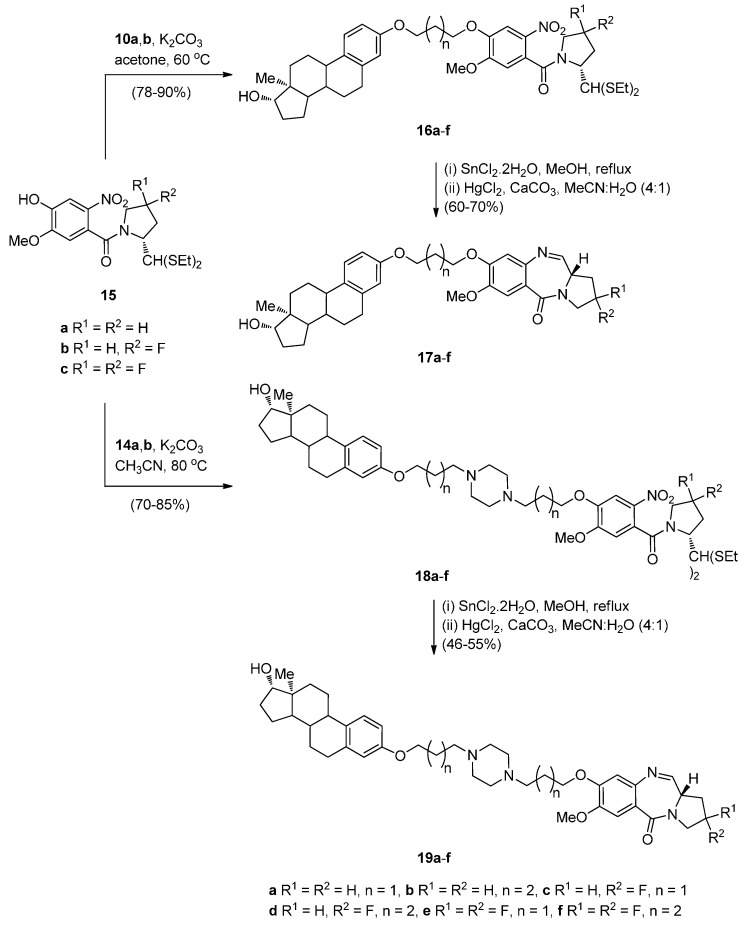
Synthesis of C8-*O*-substituted PBD conjugates **19a**–**f**.

**Scheme 3:**
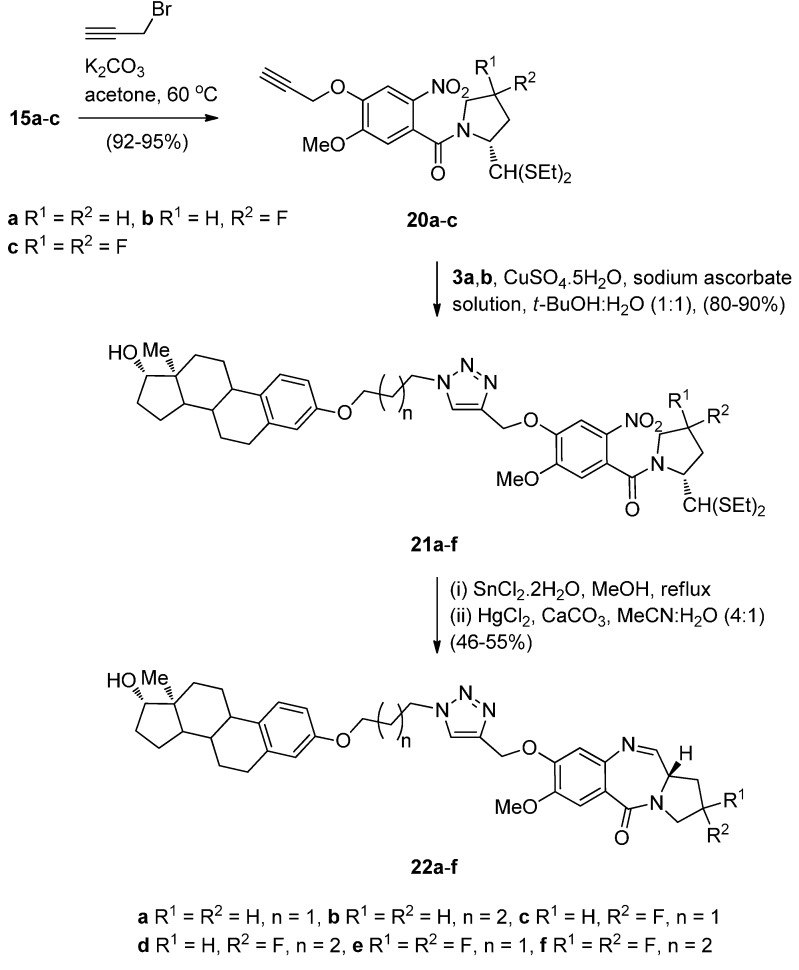
Synthesis of C8-*O*-substituted PBD conjugates **22a**–**f**.

Continuing their efforts in search of new effective anticancer agents, after having established themselves in the field of [2,1-*c*] PBDs, Kamal *et al.* [50] have designed, synthesized and studied the biological activities of several novel estradiol PBD conjugates **17a**–**f**, **19a**–**f** and **22a**–**f** (Scheme 1, Scheme 2 and Scheme 3). The convergent approach described in these schemes is the most common method of preparing PBD conjugates of this type. The synthetic strategy necessitated attaching to the phenolic side of estradiol (E2), stable bromoalkane spacers or a piperazine or 1,2,3-triazole moieties which are then linked to DC-81 (Figure 2). In the first step towards this goal estradiol derivatives **10a**,**b**, **11a**,**b** and **14a**,**b** were synthesized as outlined in Scheme 1. Estradiol **9** was alkylated with appropriate dibromoalkanes under basic conditions to afford the alkyl bromides **10a**,**b** which were transformed into azides **11a**,**b** or converted into the protected piperazine Boc derivatives **12a**,**b** and then deprotected to provide secondary amines **13a**,**b**. These amines were monoalkylated as above to the corresponding alkyl bromides **14a**,**b**.

The synthesis of estradiol PBD conjugates **17a**–**f** and **19a**–**f** required in the first step the alkylation of simple nitro-thioacetals **15a**–**c**, previously reported [49], with the corresponding alkyl bromides **10**,**b** and **14a**,**b** to produce substituted nitro-thioacetals **16a**–**f** and **18a**–**f**, respectively (Scheme 2). The method of preparing PBDs by reduction and deprotection of *N*-(2-nitrobenzoyl)pyrrolidine-2-carboxaldehyde diethyl thioacetals was also widely used up to 2010 [10]. The advantages of the diethyl thioacetal protective group are the ease with which it is introduced to an aldehyde precursor, its stability that enables, if required, a variety of chemical transformations to take place to nitro thioacetal scaffolds of type **15** and that no racemization occurs at the C11-a position of the final PBD structures. In the present synthesis, as with most syntheses of this type since 2011, the phenolic hydroxyl group of simple nitro-thioacetal scaffolds such as **15** is alkylated (*i.e.*, **15a**–**c** to **16a**–**f** and **18a**–**f** above) before reduction, deprotection and cyclisation to the final PBD. Reductive methods usually employ tin(II) chloride dihydrate (SnCl_2_·2H_2_O) in methanol and heating to reflux, as in the reduction of **16a**–**f** and **18a**–**f**, although, on several occasions hydrogenation with 10% palladium-on-carbon catalyst in methanol has been effective [10]. In general, regardless of the reduction method, after work-up, the resulting amino-thioacetals are obtained crude in 80%–90% yields and are directly used in the next step, due to potential stability problems. Thus, the crude amino-thioacetals from the tin(II) chloride dihydrate reduction of **16a**–**f** and **18a**–**f** were treated with mercury(II) chloride (HgCl_2_) and calcium carbonate and the resulting aminoaldehyde spontaneously cyclized to afford the estradiol PBD conjugates **17a**–**f** and **19a**–**f**, in 46%–70% overall yields. Although these established reaction conditions work well for this type of cyclisation method, the use of highly toxic mercury(II) chloride for deprotecting the thioacetal group does not however conform with green chemistry protocols. Nevertheless, this reagent remains in use even if efficient deprotection of thioacetals by less toxic iron(III) chloride hexahydrate or bismuth triflate has been reported [10]. Moreover, it seems that the use of the thioacetal protective group has not yet been overcome due to its efficiency, even if handling ethyl mercaptan during the protection/deprotection steps is inconvenient due to its pungent smell.

Compounds **15a**–**c** were alkylated with propargyl bromide in base to afford the terminal alkynes intermediates **20a**–**c** in high yields (Scheme 3). These were further reacted with azides **11a**,**b** to undergo the “click” reaction in the presence of *in situ* generated Cu(I) ions, to produce the estradiol 1,2,3-triazolenitrothioacetals **21a**–**f** in over 80% yields. Although this “click” chemistry has been used for the first time in 2011 to synthesize C8–triazole linked PBD conjugates of type **22**, the same author has previously reported [10] triazole linked PBD dimers and trimers where the cycloaddition step proceeded in comparable yields. Under the established reaction conditions, described above, compounds **21a**–**f** were converted into estradiol PBD conjugates **22a**–**f**, in moderate yields. PBD conjugates **22a**,**b** are derivatives where the estradiol pharmacophore is linked to the C8-position of DC-81.

**Scheme 4:**
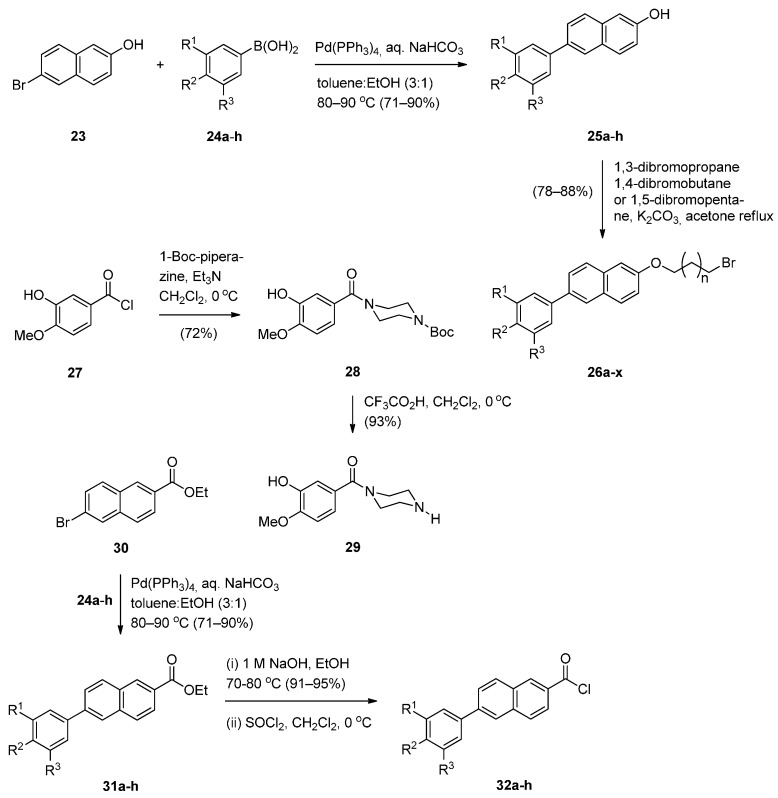
Synthesis of 2-methoxy-5-(piperazin-1-ylcarbonyl)phenol **29** and acid chlorides **32a**–**h**.

**Scheme 5:**
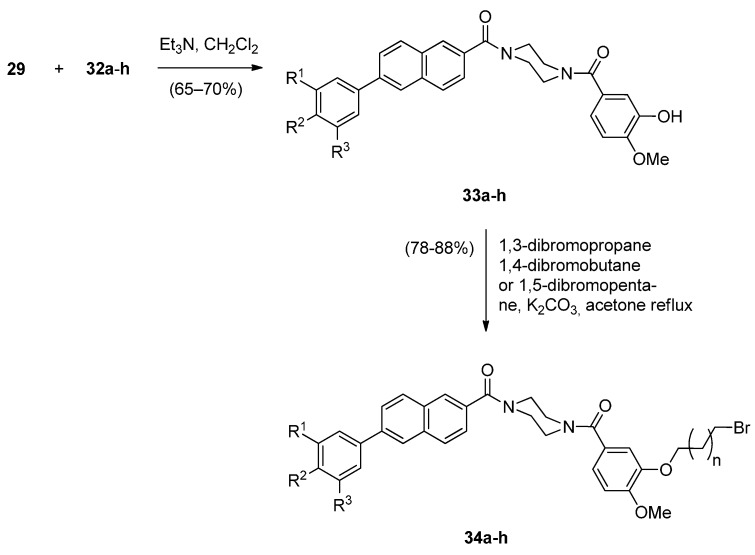
Synthesis of bromoalkyl derivatives **34a**–**h**.

**Scheme 6:**
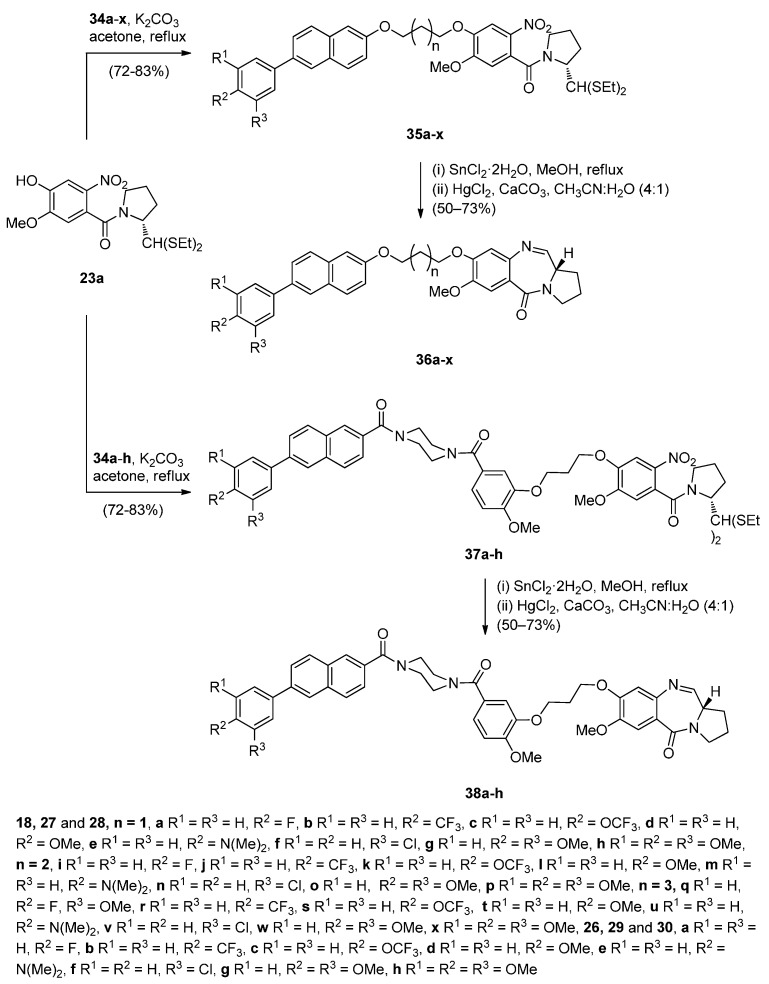
Synthesis of C8-*O*-substituted PBD conjugates **38a**–**h**.

The significant anticancer activity of both piperazine and naphthalene derivatives as well as piperazine-containing PBDs, motivated Kamal *et al.* [51] to explore various aryl-substituted naphthalene derivatives linked to the C8-position of DC-81 through stable alkane linkers and also by incorporating a piperazine moiety, as pharmacophores in the design of novel monomeric PBD conjugates **36a**–**x** and **38a**–**h**. The precursors for the synthesis of these PBDs are the bromoalkyl derivatives **26a**–**x** and **34a**–**h** that are prepared as shown in Scheme 4 and Scheme 5. The difference between the synthetic strategies towards PBDs **36a**–**x** and **38a**–**h** (Scheme 6) and PBDs **22a**–**f** (Scheme 3) is the method by which the non-PBD pharmacophores are linked to the hydroxyl group of the nitro-thioacetal precursors **15a**–**c**. Bromoalkyl derivatives **26a**–**x** were prepared in two steps from 6-bromo-2-naphthol **23** phenylboronic acids **24a**–**h** by a Suzuki palladium-catalyzed cross coupling reaction followed by an alkylation reaction with appropriate dibromoalkanes. 3-Bromopropyl 2-naphthyl ethers **34a**–**h** were prepared by acyl substitution of acid chlorides **32a**–**h** by 2-methoxy-5-(piperazin-1-ylcarbonyl)phenol **29** to give diamides **33a**–**h** which were alkylated by 1,3-dibromopropane (Scheme 5).

In the final two steps towards target C8-substituted PBDs **36a**–**x** and **38a**–**h** (Scheme 6) the hydroxyl group of dithioacetal **15a** was alkylated by the bromoalkyl derivatives **26a**–**x** and **34a**–**h** to provide the respective nitro-thioacetals **35a**–**x** and **37a**–**h**, which upon reduction of the nitro group to amino with tin(II) chloride dihydrate followed by mercury(II) chloride and calcium carbonate deprotection and subsequent spontaneous cyclisation of the resulting amino aldehyde, afforded the required PBD conjugates **36a**–**x** and **38a**–**h**, in yields ranging from 78% to 88%. The cyclisation of nitro-thioacetals **21a**–**f** (Scheme 3) by a similar manner gave much lower yields (46%–55%) of PBD conjugates **22a**–**f**.

**Scheme 7:**
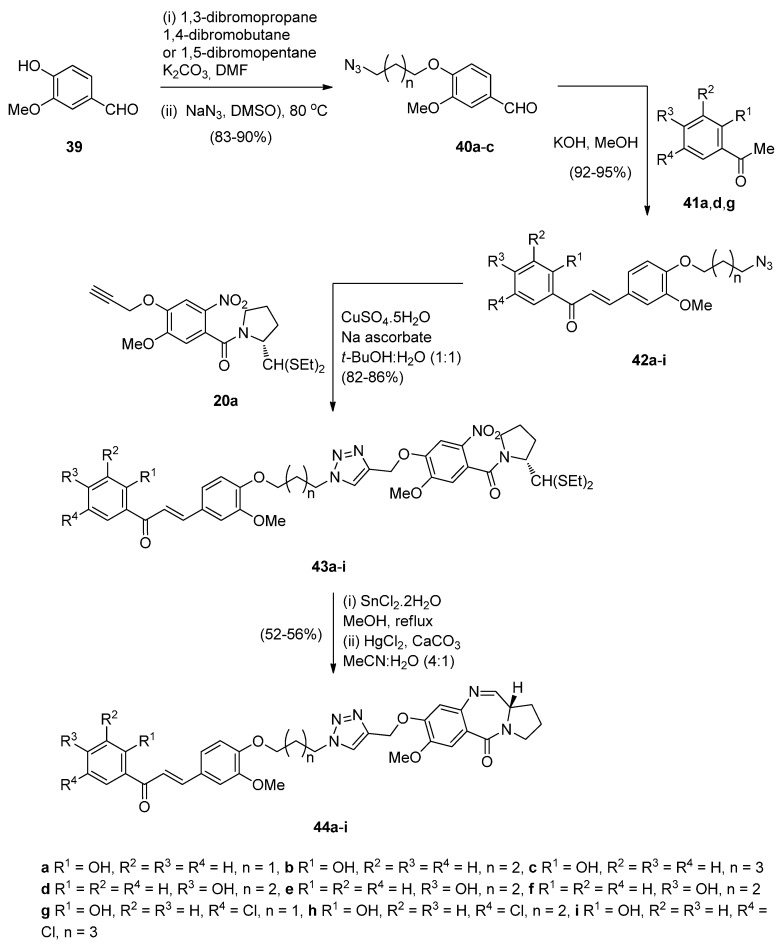
Synthesis of C8-*O*-substituted PBD conjugates **44a**–**i**.

**Scheme 8:**
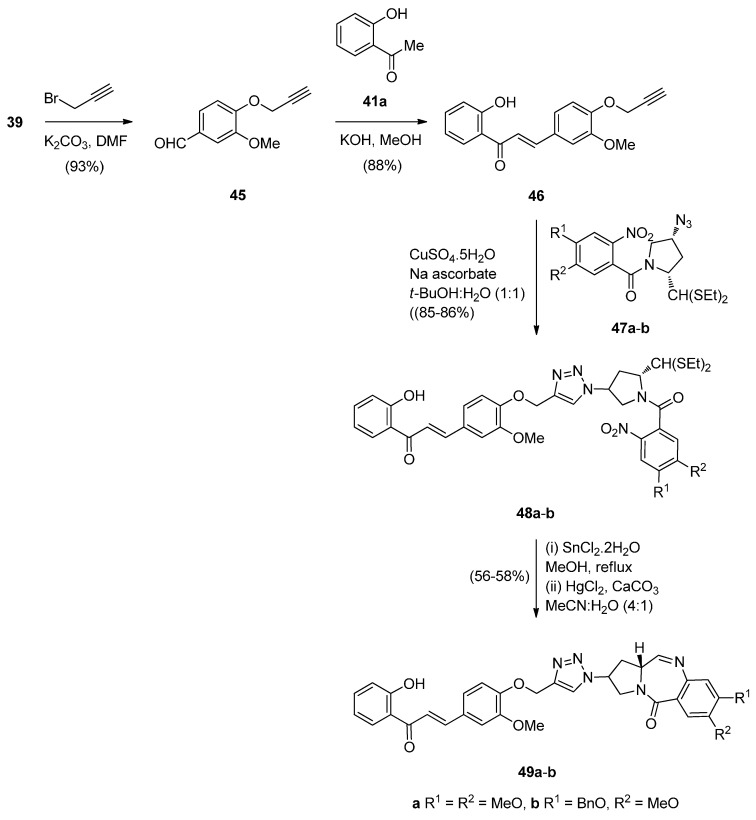
Synthesis of C2-substituted PBD conjugates **49a**–**b**.

Although chalcones and 1,2,3-triazoles are known to exhibit anticancer activity, chalcone has been further involved as a scaffold in structural modifications for the development of anticancer agents. With this in mind, Kamal *et al.* [52] set out to study the synthesis and biological evaluation of monomeric triazolochalcone-PBD conjugates **44a**–**i** and **49a**,**b** (Scheme 7 and Scheme 8). The synthetic strategy relies upon two key intermediates, that is azidochalcones **42a**–**e** and alkyne **20a**, which via 1,3-dipolar cycloadditions will provide the triazole derivatives **43a**–**i**, that will then lead to the monomeric PBD conjugates **44a**–**i** by the appropriate ring closure. This synthetic procedure is quite similar to that described by the same author in Scheme 3 where the non-PBD pharmacophore contains an azide group which undergoes cycloaddition with an alkyne substituted nitro thioacetal to form the connective triazole ring prior to cyclisation to the PBD conjugate. Azidochalcones **42a**–**e** (Scheme 7) were prepared by etherification of vanillin **39** with suitable dibromoalkanes followed by azidation of the non-isolable bromoalkylethers to produce the corresponding azido precursors **40a**–**c** which were then condensed with acetophenones **41a**,**d**,**g**. At this stage the “click” chemistry protocol was introduced between alkyne **20a** and azides **42a**–**e** to afford nitro intermediates **43a**–**i**. Reduction, deprotection and cyclocondensation by the established reaction conditions (Scheme 7) afforded the PBD conjugates **44a**–**i**.

The authors diversified this synthetic methodology for the synthesis of PBD conjugates **49a**,**b** (Scheme 8) by tethering the alkyne moiety on the non-PBD pharmacophore and introducing the azide group on the pyrrolidine ring of the nitro-thioacetal precursor. Thus, cycloaddition of alkyne **46** with azides **47a**,**b** provided 1,2,3-triazoles **49a**,**b** which cyclized under the established reaction conditions of the authors, to provide PBD conjugates **49a**,**b** in 52%–56% yields. The moderate yields of these cyclisations are comparable to the yields (45%–55%) of PBD conjugates **22a**–**f** (Scheme 3).

**Scheme 9:**
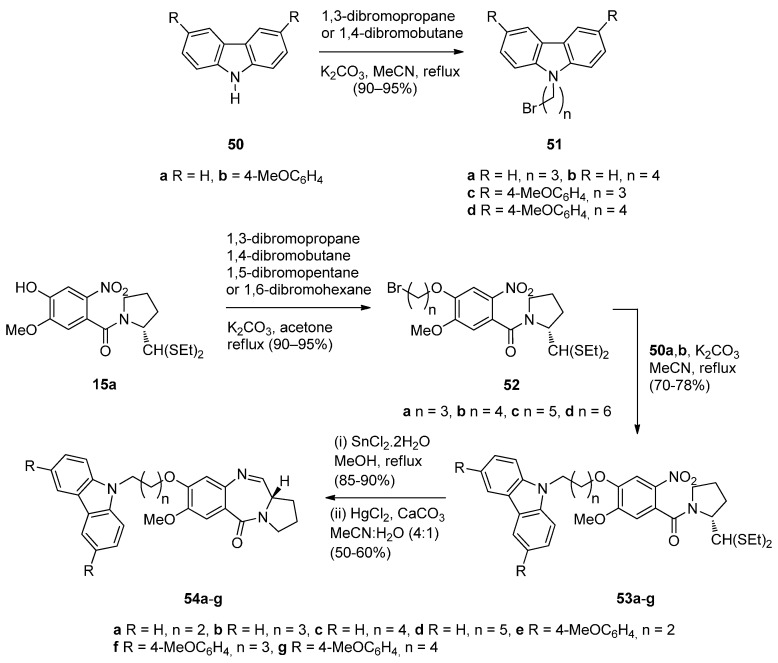
Synthesis of C8-*O*-substituted PBD conjugates **54a**–**g**.

**Figure 4:**
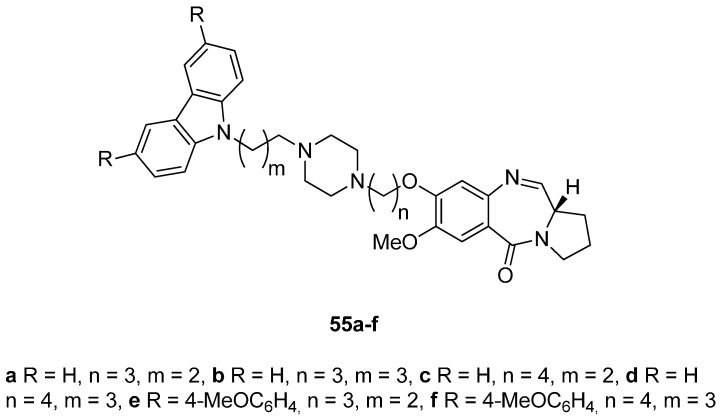
C8-*O*-substituted PBD conjugates **55a**–**f**.

Carbazoles belong to the unusual class of DNA binding agents while the carbazole scaffold is found in many synthetic anticancer agents. These facts encouraged Kamal *et al.* [53] to synthesise a series of monomeric C8–carbazole PBD conjugates **54a**–**g** and **55a**–**f** (Scheme 9 and Figure 4) and to determine their DNA binding ability by thermal denaturation studies that were supported by molecular docking studies. The carbazole bromoalkyl spacers **51a**–**d** were prepared by *N*-alkylation of carbazoles **50a**,**b** with suitable dibromoalkanes. The thioacetal **15a**, previously reported [48], was alkylated by the appropriate dibromoalkane, according to Thurston *et al.* [54] to provide bromoalkyl nitro-thioacetals **52a**–**d** which further reacted with carbazoles **50a**,**b** and nitro-thioacetals **53a**–**g**. Cyclisation of these compounds by the authors’ established reaction conditions (Scheme 9) produced the C8–carbazole PBD conjugates **54a**–**g** in 56%–58% yields that are comparable to similar type of cyclisations leading to PBD conjugates **22a**–**f** (45%–55%) (Scheme 3) and PBD conjugates **44a**–**i** (52%–56%) (Scheme 7).

By reacting **52a**,**b** first with 1-Boc-piperazine followed by deprotection of the Boc adduct to the secondary amine, then alkylation of this amine by bromoalkylcarbazoles **51a**–**d**, reduction of the nitro group of these products to amino and lastly deprotection and cyclisation, by a manner analogous to the steps of Scheme 9, furnished C8–carbazole-piperizinal PBD conjugates **55a**–**f** (Figure 4) in good yields.

**Scheme 10:**
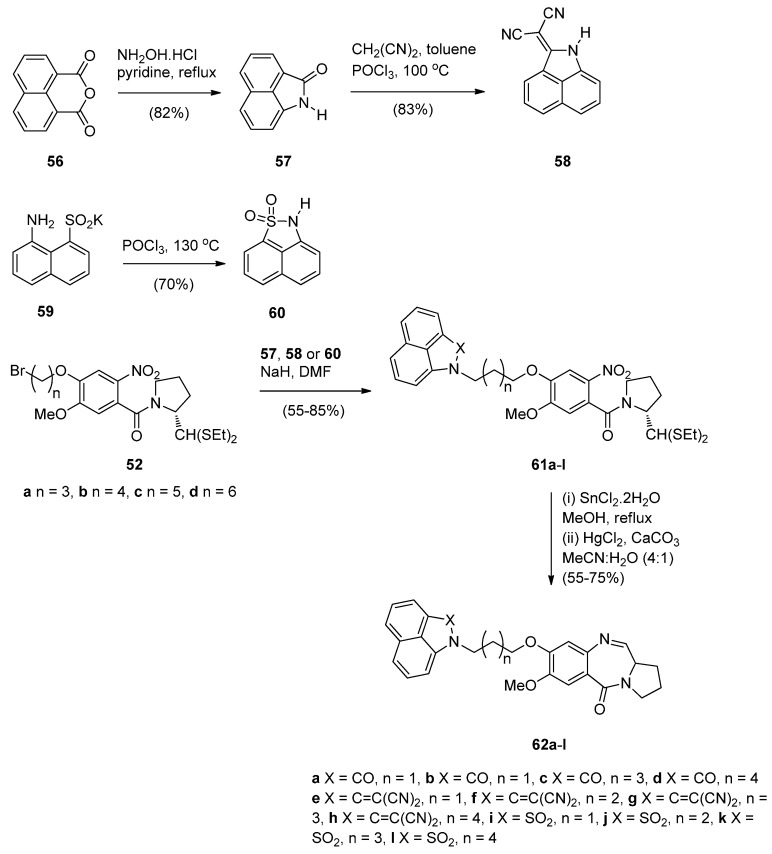
Synthesis of C8-*O*-substituted PBD conjugates **62a**–**l**.

In view of a recent report that describes certain benzo[*c*,*d*]indol-2(1*H*)one derivatives as novel DNA intercalators and as efficient antitumour agents, Kamal *et al.* [55] designed and synthesised a series of monomeric C8–benzo[*c*,*d*]indol-2(1*H*)one PBD conjugates **62a**–**l** as potential anticancer agents. The DNA binding ability of these conjugates was evaluated as well as their anticancer activity. The synthetic strategy for these PBDs (Scheme 10) involves first the preparation of benzo[*c*,*d*]indol-2(1*H*)-one (**57**), benzo[*c*,*d*]indol-2(1*H*)-ylidenemalononitrile (**58**) and 2*H*-naphtho-[1,8-*c*,*d*]isothiazole 1,1-dioxide (**60**) from 1*H*,3*H*-benzo[*d*,*e*]isochromene-1,3-dione (**56**) and [(8-amino-1-naphthyl)sulfonyl]potassium (**59**). In the next steps, indolone derivatives **57**, **58** and **60** were alkylated by bromoalkane-thioacetals **52a**–**d**, prepared earlier [54], to afford the respective nitro- thioacetals **61a**–**l**. By using the authors’ established reaction conditions compounds **61a**–**l** were converted into the corresponding C8–substituted PBD conjugates **62a**–**l** in 55%–75% yields that are on average slightly better than yields of similar cyclisations previously described such as PBDs **22a**–**f** (45%–55%) (Scheme 3), PBDs **44a**–**i** (52%–56%) (Scheme 7) and PBDs **54a**–**g** (50%–60%) (Scheme 9).

**Scheme 11:**
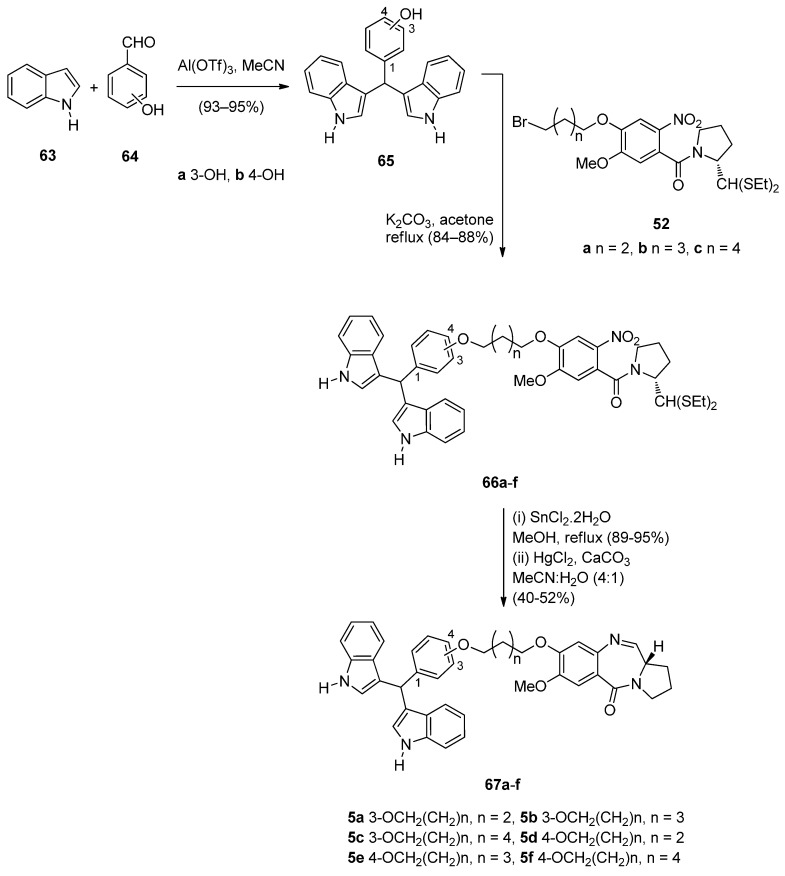
Synthesis of C8-*O*-substituted PBD conjugates **67a**–**f**.

Several bisindolyl methanes have recently been found to exhibit various anticancer effects and it was for this reason that Kamal *et al.* [56] decided to select and synthesize the six monomeric bisindole-PBDs **67a**–**f** and to study their anticancer properties. In the first step of the synthetic route towards these PBDs (Scheme 11), 3(or 4)-(di-1*H*-indol-3-ylmethyl)phenols **65a**,**b** were prepared from the reaction of 3-hydroxybenzaldehyde (**64a**) or 4-hydroxybenzaldehyde (**64b**) and 1*H*-indole (**63**) using aluminium triflate [Al(OTf)_3_] as a catalyst. Alkylation of phenolic bisindoles **65a**,**b** by the bromoalkyl derivatives **52a**–**c** [54], afforded nitro-thioacetals **66a**–**f**. Next, by applying the authors’ established reaction conditions [49] compounds **66a**–**f** were transformed into the C8-linked bisindole PBD conjugates **67a**–**f**.

**Scheme 12:**
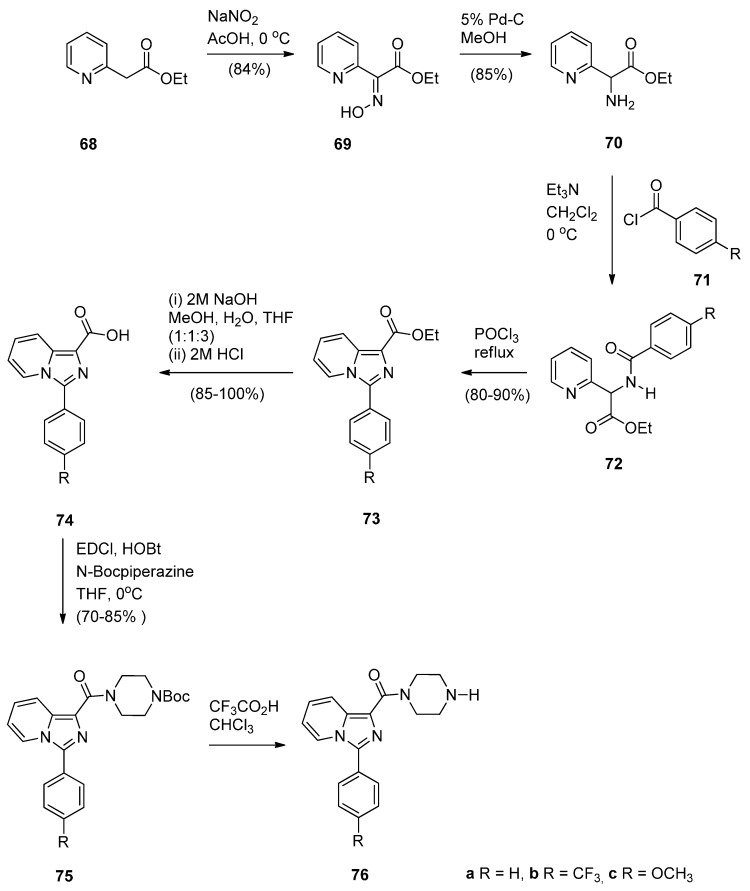
Synthesis of imidazo[1,5-*a*]pyridine precursors **76a**–**c**.

**Scheme 13:**
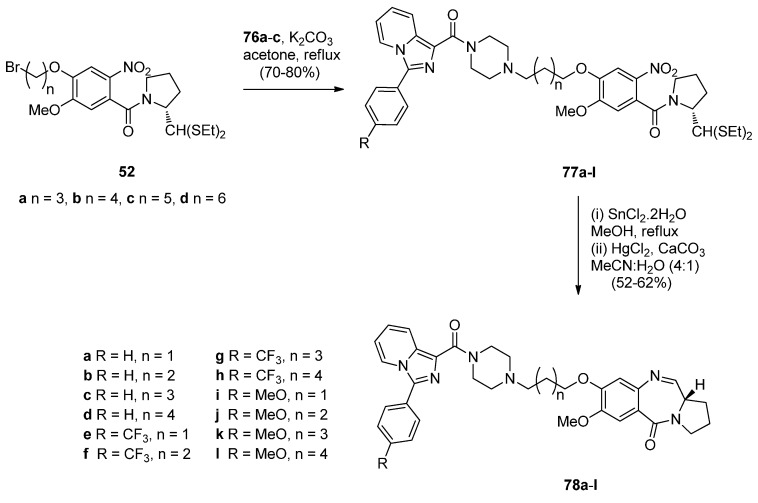
Synthesis of C8-*O*-substituted PBD conjugates **78a**–**l**.

As part of their work on the synthesis and study of PBD conjugates as DNA interactive drugs, Kamal *et al.* [57] became interested in imidazo[1,5-*a*]pyridine, a known pharmacological scaffold, and prepared a new class of imidazo[1,5-*a*]pyridine-piperazine linked PBD conjugates **79a**–**l**, in order to evaluate their antitumour potential. The synthetic strategy involves preparing the 3-ary-1-(piperazin-1-ylcarbonyl)imidazo[1,5-*a*]pyridines **76a**–**c** (Scheme 12) and the 1-(4-bromoalkoxy-5-methoxy-2-nitrobenzoyl]pyrrolidines **77a**–**d** (Scheme 13) and then linking the two to produce eventually the target compounds **79a**–**l**. The synthesis of precursors **76a**–**c** requires in the first step diazotisation of ethyl 2-(2-pyridyl) acetate **68** to give ethyl 2-hydroxyimino-2-(2-pyridyl) acetate (**69**). Catalytic hydrogenation of the latter afforded amino intermediate **70** which upon treatment with substituted acid halides **71a**–**c** gave the corresponding amides **72a**–**c**. Cyclisation of amides **72a**–**c** with phosphorous oxychloride provided imidazo[1,5-*a*]pyridines **66a**–**c** and then de-esterification of these, afforded the corresponding acids **74a**–**c**. Coupling of acids **74a**–**c** with 1-Boc-piperazine gave amides **75a**–**c**. Deprotection of **75a**–**c** produced the desired precursors **76a**–**c** as shown in Scheme 12. The route to PBD conjugates **78a**–**l** (Scheme 13) was carried out by applying established chemistry described by these authors [49] (*vide supra*).

**Scheme 14:**
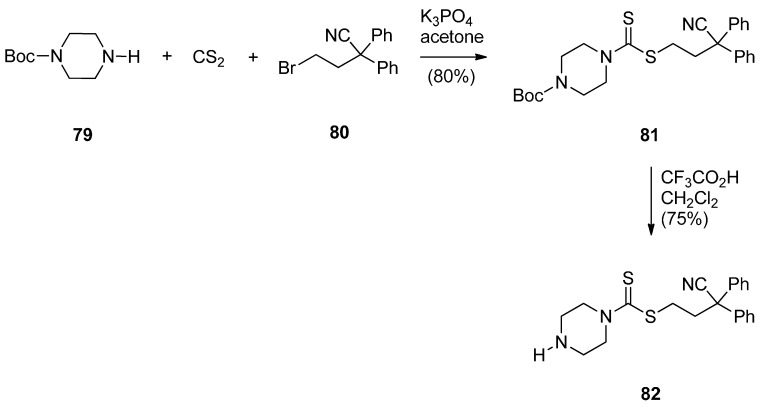
Synthesis of carbodithioate derivative **82**.

**Scheme 15:**
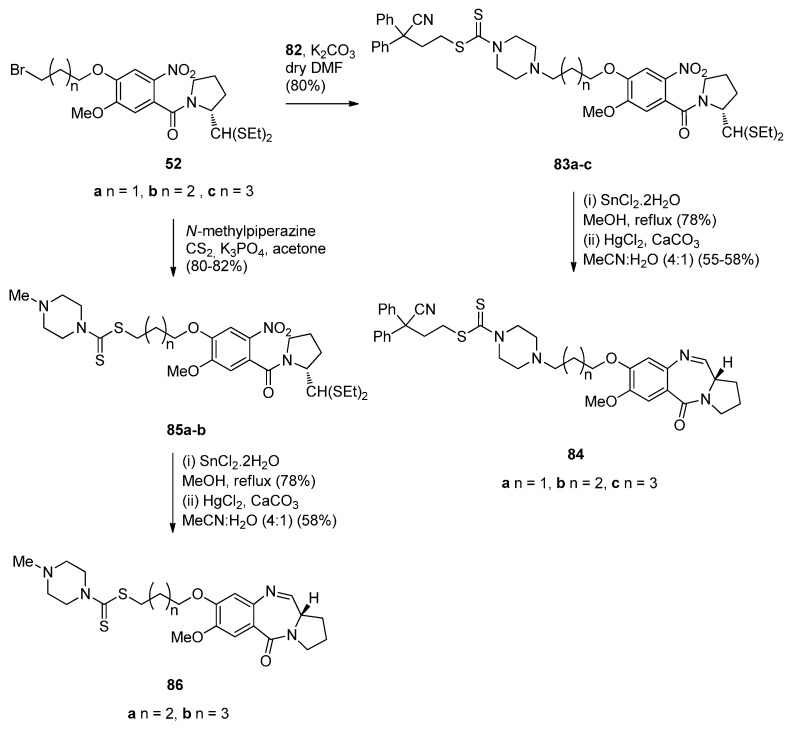
Synthesis of C8-*O*-substituted PBD conjugates **84a**–**c** and **86a**,**b**.

A novel series of C8-linked dithiocarbamate/piperazine bridged PBD conjugates **84a**–**c** and **86a**,**b** were prepared (Scheme 14 and Scheme 15) and evaluated for their cytotoxic potential and DNA-binding ability by Kamal *et al.* [58]. The synthesis of target compounds **84a**–**c** required in the first step an S*_N_*2 reaction between carbodithioate **82** and bromoalkyl nitro-thioacetals **52a**–**c**, prepared previously [54], which gave nitro-thioacetals **83a**–**c**, in very good yields. Precursor **82** was prepared by addition of carbon disulfide to 1-Boc-piperazine **79**, alkylation by propylbromide **80** to produce conjugate **81** and then deprotection with trifluoroacetic acid (Scheme 14).

*O*-substituted nitro-thioacetals **83a**–**c** prepared by alkylating **82** by **52a**–**c** and *O*-substituted nitro-thioacetals **85a**,**b** prepared by alkylating *N*-methylpiperazine with **52b**,**c** were efficiently reduced by stannous (II) chloride dihydrate (SnCl_2_·2H_2_O) to afford the corresponding amino-thioacetals which were deprotected using mercuric(II) chloride (HgCl_2_) and calcium carbonate (CaCO_3_) and cyclocondensed, according to the authors’ established reaction procedures [48] (Scheme 15), to afford PBD conjugates **84a**–**c** and **86a**,**b** in 55%–58% and 58% yields, respectively. These yields are comparable to yields of previous similar cyclisations that give PBDs **22a**–**f** (45%–55%) (Scheme 3), PBDs **44a**–**i** (52%–56%) (Scheme 7) PBDs **54a**–**g** (50%–60%) (Scheme 9), **67a**–**f** (40%–52%) (Scheme 11) and **79a**–**l** (52%–62%) (Scheme 13), with the exception of PBDs **62a**–**l** (55%–75%) where yields are on average slightly higher.

This synthetic approach to the PBD conjugates that is, by using a bromoalkyl nitro-thioacetal (*i.e.*, **52**) and then coupling with a non-PBD pharmacophore, has been previously described by these authors [53] (Scheme 9), [55] (Scheme 10) and [57] (Scheme 13).

The last two synthetic steps described so far from the work of Kamal *et al.* [47,49,50,51,53] and [54,55,56], all involve the reduction of the nitro group of an *N*-(2-nitrobenzoyl)pyrrolidine-2-carboxaldehyde diethyl thioacetal derivative to the corresponding amino derivative and workup of the reaction mixture without characterizing the product or checking its purity (TLC). The authors however report that the nitro compounds are efficiently reduced to amino compounds and due to potential stability problems they proceed to the next step. The yields of these crude *N*-(2-amino-benzoyl)pyrrolidine-2-carboxaldehyde diethyl thioacetal derivatives are given in three papers [51] (85%–90%), [54] (89%–95%) and [56] (78%).

**Scheme 16:**
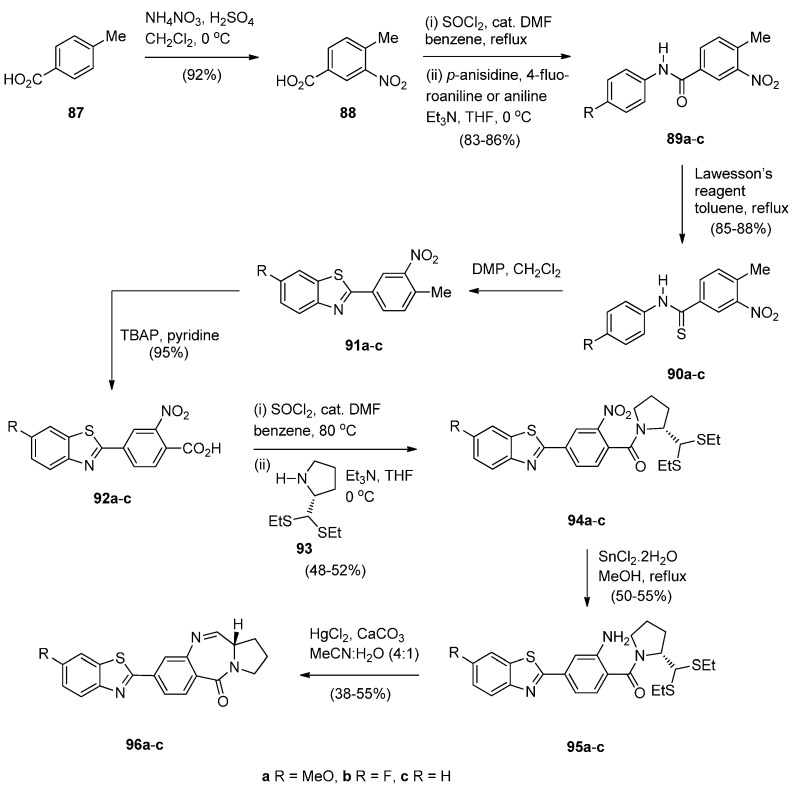
Synthesis of C8-substituted PBD conjugates **96a**–**c**.

Bose *et al.* [59] became interested in synthesising benzothiazole (BTA) PBD conjugates **95a**–**c** after the recognition of 4-(1,3-benzothiazol-2-yl)-2-methylaniline and two of its derivatives as novel anticancer drug candidates. The synthetic plan required first the synthesis of the key intermediate 4-(6-substituted-1,3-benzothiazol-2-yl)-2-nitrobenzoic acids **92a**–**c** from 4-methylbenzoic acid (**87**) (Scheme 16). Thus, **87** was nitrated to nitro derivative **88** which was then converted into the corresponding acid chloride that was used in crude form in the acyl substitution with *p*-anisidine, 4-fluoroaniline or aniline to afford amides **89a**–**c**. Amides **89a**–**c** were then treated with Lawesson’s reagent to afford thioamides **90a**–**c** which were cyclised using Dess-Martin periodinane (DMP), to yield benzothiazoles **91a**–**c**. The methyl group of compounds **91a**–**c** was oxidised with tetrabutylammonium permanganate (TBAP) to afford the corresponding benzothiazolyl-2-benzoic acids **92a**–**c**. The next step required the coupling of the acid chlorides of **92a**–**c** with (2*S*)-2-[bis(ethylthio)methyl]pyrrolidine (**93**) to afford the amides **94a**–**c**. Although **93** has been synthesized earlier by Langley and Thurston [60] here it was prepared in better yields with excellent optical purity in five steps from (*S*)-proline. Following established reaction conditions [49], reduction of nitro compounds **94a**–**c** to the corresponding amines **95a**–**c** followed by their deprotection and spontaneous cyclodehydration, afforded the corresponding BTA PBD conjugates **96a**–**c**, in moderate yields. This synthetic approach to the PBDs differs in the last but two steps from the syntheses in Scheme 1, Scheme 2, Scheme 3, Scheme 4, Scheme 5, Scheme 6, Scheme 7, Scheme 8, Scheme 9, Scheme 10, Scheme 11, Scheme 12, Scheme 13, Scheme 14 and Scheme 15, in that the non-PBD pharmacophore is incorporated as a substituent at position 4 of 2-nitrobenzoic acids **92a**–**c** and is then coupled to pyrrolidine **93** to produce the *N*-(2-nitro-benzoyl)pyrrolidine-2-carboxaldehyde diethyl thioacetals **94a**–**c**. Furthermore, reduction of the latter derivatives led to *N*-(2-aminobenzoyl)pyrrolidine-2-carboxaldehyde diethyl thioacetals **95a**–**c** that after workup were purified by column chromatography and characterized by ^1^H-NMR, low resolution MS and IR spectroscopy, the first time for these type of compounds.

##### Oxidative Cyclisation of *N*-(2-(Alloc or Troc)aminobenzoyl)pyrrolidine-2-Methanol Derivatives

**Scheme 17:**
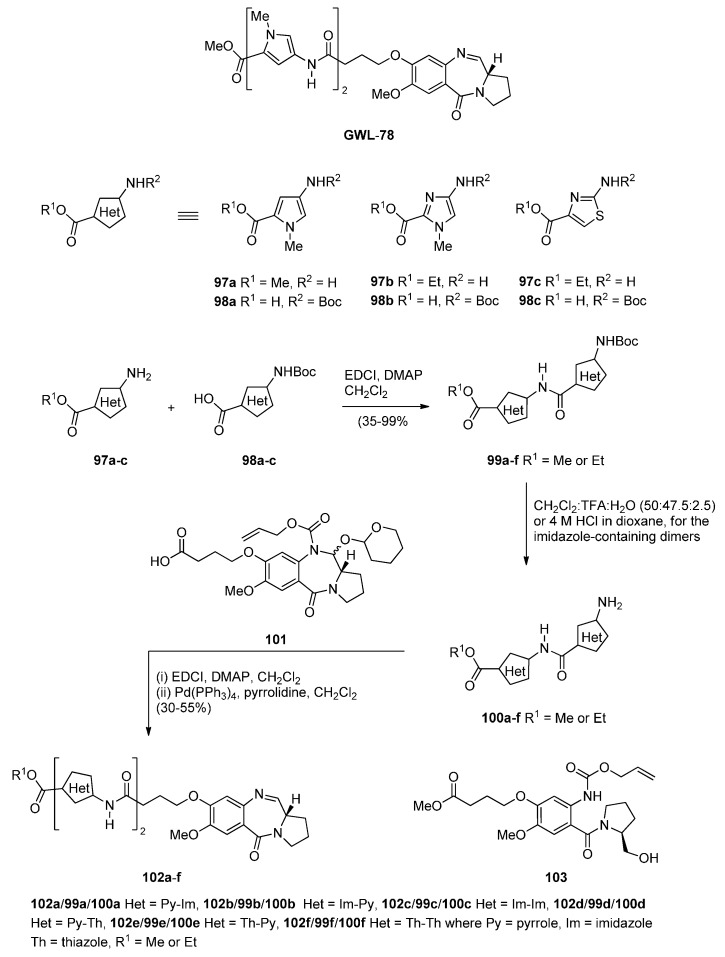
Synthesis of C8-*O*-substituted PBD conjugates **102a**–**f**.

The interest in earlier work by Thurston and co-workers [61,62] who synthesied a series of C8-linked poly(*N*-methylpyrrole) PBD conjugates (e.g., **GWL**-**78**) and demonstrated their ability to interact with a variety of CCAAT-containing promoters leading to p53-independentcell cycle arrest, was renewed and six C8-linked poly(heterocyclic) PBD conjugates **102a**–**f** (Scheme 17) were synthesized in order to study further this biological interaction [63]. In the synthetic route to **102a**–**f** the starting heterocyclic amino esters **97a**–**c** and the Boc-protected heterocyclic amino acids **98a**–**c** were synthesized as previously reported [61]. These compounds were then coupled together using standard methodology to produce the Boc-protected imidazole **99a**–**c** and thiazole **99d**–**f** dimers which, without purification, were deprotected with trifluoroacetic acid to provide dimeric amino esters **100a**–**f**. These amines were then coupled to the carboxylic acid group of previously described [62] C8-(3-carboxypropoxy)-N10-alloc-C11-*O*-THP protected PBD **101**, under standard reaction conditions, followed by treatment with tetrakis(triphenylphosphine)palladium(0) [Pd(Ph_3_P)_4_] and pyrrolidine that promotes simultaneous removal of the alloc and THP protecting groups to afford C8-linked poly(heterocyclic) PBD imine conjugates **102a**–**f**, in moderate to good yields. PBD **101** is prepared in three steps from *N*-(2-alloc protected aminobenzoyl)pyrrolidine-2-methanol derivative **103** by Swern oxidative cyclisation to an C8-(3-methoxycarbonylpropoxy)-N10-alloc-C11-OH PBD, protection to a C8-(3-methoxycarbonylpropoxy)-N10-alloc-C11-*O*-THP PBD and hydrolysis of the ester group to a carboxylic acid group.

This methodology differs from those so far described in that a PBD is constructed with a C8 substituent possessing a carboxylic acid end group. The rest of the C8 moiety is then extended by coupling this carboxylic acid group with the amino group of preformed 5-membered heterocyclic aminoester dimers.

**Scheme 18:**
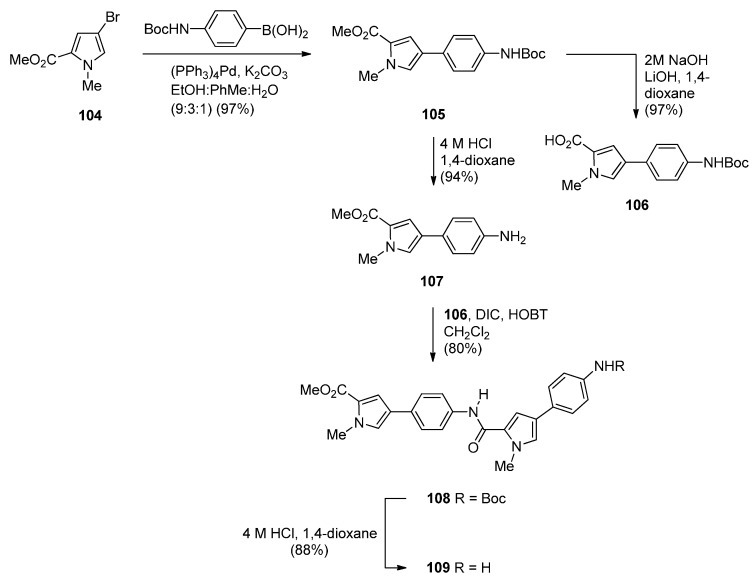
Synthesis of aminoester precursors **107** and **109**.

**Scheme 19:**
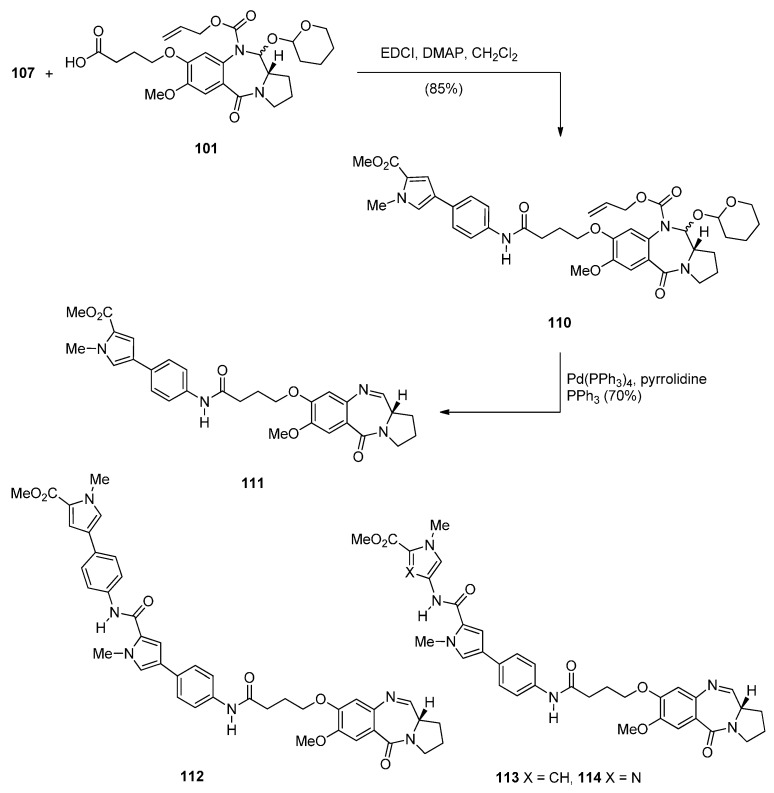
Synthesis of C8-*O*-substituted PBD conjugates **111**–**114**.

**Scheme 20:**
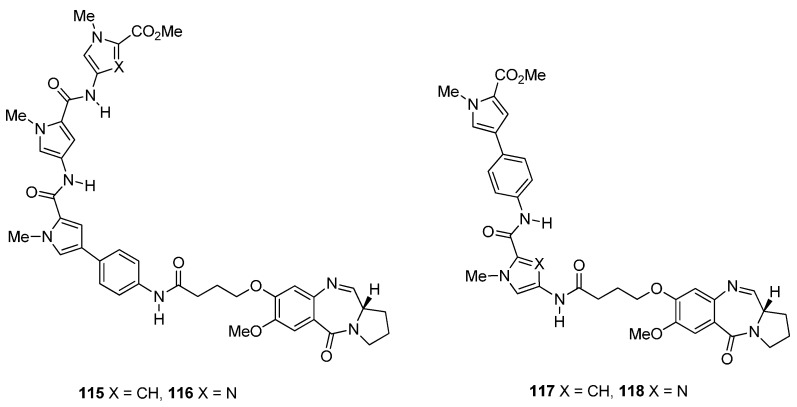
Synthesis of C8-*O*-substituted PBD conjugates **115**–**118**.

Two years later Thurston and co-workers presented [64] a continuation of their previous work [61] on the synthesis and biological study of C8-linked poly(*N*-methylpyrrole) PBD conjugates and used similar methodology to synthesise seven biaryl(poly)amide PBD conjugates **111**–**118**. The simplest of these compounds, **111**, was prepared in two steps from the smallest biaryl building block, namely methyl 4-(4-aminophenyl)-1-methyl-1*H*-pyrrole-2-carboxylate (**107**, Scheme 18). Coupling **107** with PBD-carboxylic acid **101** under standard reaction conditions afforded PBD adduct **110**, in very good yield. The N10–alloc and C11–OTHP protecting groups of **110** were then simultaneously removed with tetrakis(triphenylphosphine)palladium(0) [(PPh_3_)_4_Pd] catalyst and pyrrolidine to afford **111** in good yield. Although PBD **101** has been synthesized earlier [61], aminoester **107** was prepared from commercially available bromoester **104** in two steps. Suzuki coupling between bromoester **104** and 4-[(*tert*-butoxycarbonyl)amino]phenylboronic acid took place with tetrakis(triphenylphosphine)-palladium(0) [(PPh_3_)_4_Pd] catalyst and potassium carbonate in a mixture of ethanol, toluene and water (9:3:1) under microwave irradiation for 8 minutes, to produce 4-arylpyrrole **105** in excellent yield. Deprotection of the Boc group of **105** afforded the aminoester **107**. Building block **109** R=H was synthesized as shown in Scheme 18 and served to prepare biarylpolyamide PBD conjugate **112** by coupling with PBD-carboxylic acid **101** and then deprotecting the product obtained, in a manner similar to that described for compound **111** in Scheme 19. Conjugates **113**–**118** were prepared by analogous reactions (Scheme 19 and Scheme 20).

**Scheme 21:**
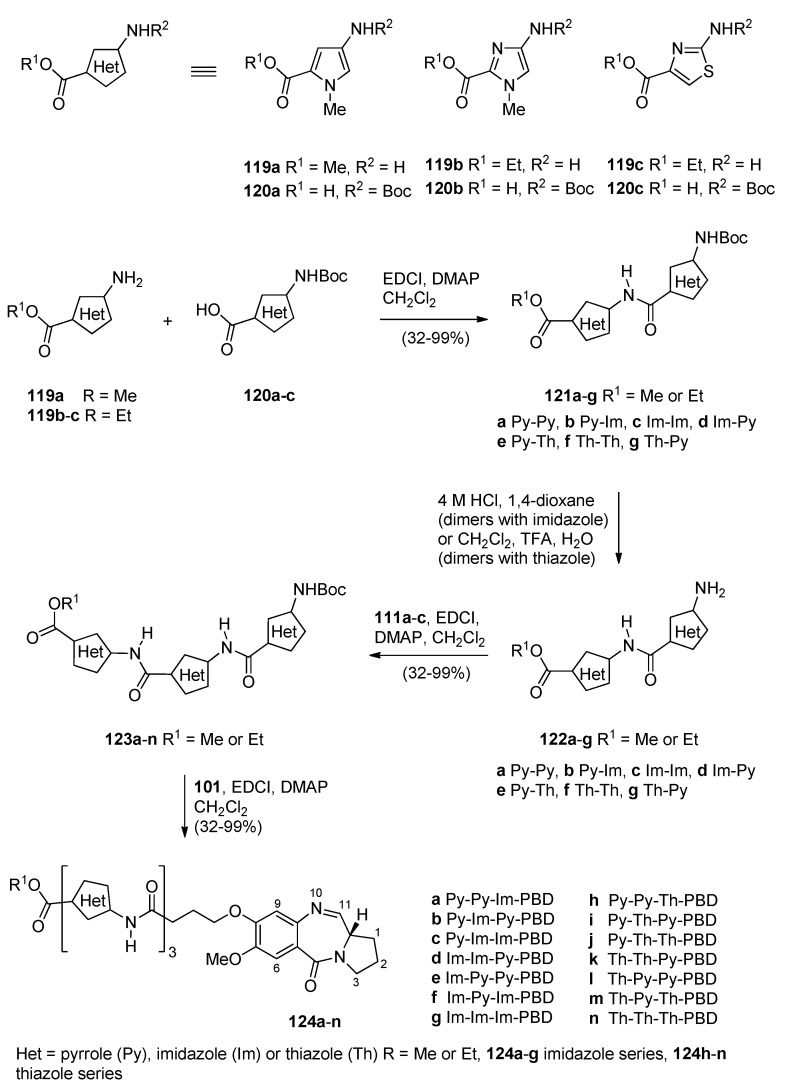
Synthesis of C8-*O*-substituted PBD conjugates **124a**–**n**.

Based on recent findings that the binding of nuclear factor Y (NF-Y) to inverted CCAAT boxes (ICBs), in the Topo IIα cellular region, a target for anticancer agents, Thurston and co-workers [65] prepared a library of PBD conjugates where one PBD molecule is linked via the C8 oxygen atom to an ethane moiety which is joined to three alternative amide/5-membered heterocyclic ring units **124a**–**n** (Scheme 21). These are potential NF-Y inhibitory molecules that were then studied as inhibitors against the replication of cancer cells. This synthetic procedure is similar to the previous two synthetic procedures as far as the preparation of PBD-carboxylic acid **93** and its coupling with preformed 5-membered heterocyclic amino ester trimers **123a**–**n**, is concerned. Thus, for the preparation of heterocyclic trimers **123a**–**n** the solution phase approach was used to couple amino ester heterocycles **119a**–**c** to the Boc-protected heterocyclic acids **120a**–**c** to provide the Boc-protected heterocyclic dimers **121a**–**g** in sufficient yield and purity, to be used directly in the following deprotection step. It was found that 4 M hydrochloric acid in dioxane was a suitable deprotection system for the imidazole/pyrrole-containing dimers, whereas a mixture of dichloromethane–trifluoroacetic acid–water (50:47.5:2.5 *v*/*v*) was required for the thiazole/pyrrole-containing dimers. The resulting amino ester dimers **122a**–**g** were subsequently coupled to the Boc-protected acids **120a**–**c** to furnish the Boc-protected trimers **123a**–**n**. The free amines of **123a**–**n** were then coupled to the previously described [61] PBD carboxylic acid **101** to afford PBD conjugates **124a**–**n**, in overall good yields. The synthesized PBD conjugates **124a**–**n** underwent DNA thermal denaturation experiments to determine their DNA-binding affinity, the DNase I footprinting assay to determine both their DNA binding affinity and sequence selectivity, the electrophoretic mobility shift assay to measure the inhibition of NF-Y binding to the ICB1 and ICB2 sites and were evaluated for their *in vitro* toxicity at the NCI. Furthermore, following the discovery that PBD conjugate **124a** is a potent of NF-Y binding to the ICB2 site, molecular modeling gave an insight of its covalent interaction with DNA.

**Scheme 22:**
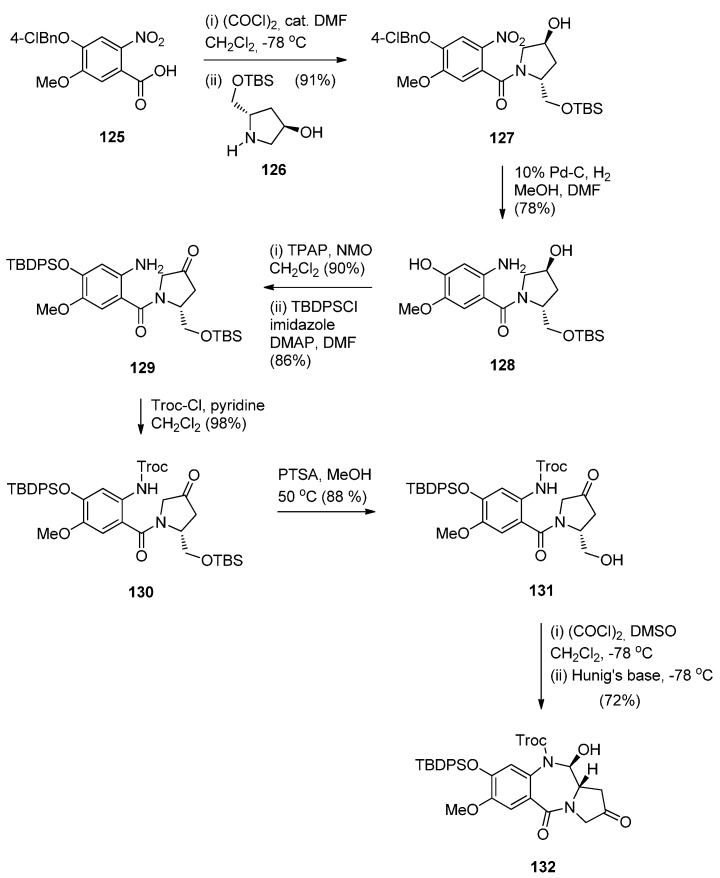
Synthesis of C8-*O*-protected PBD precursor **132**.

**Scheme 23:**
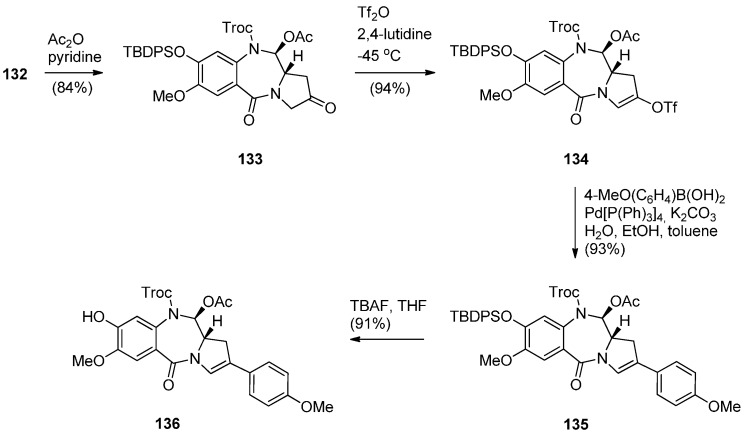
Synthesis of C8-OH N10-Troc C11-OAc PBD precursor **136**.

**Scheme 24:**
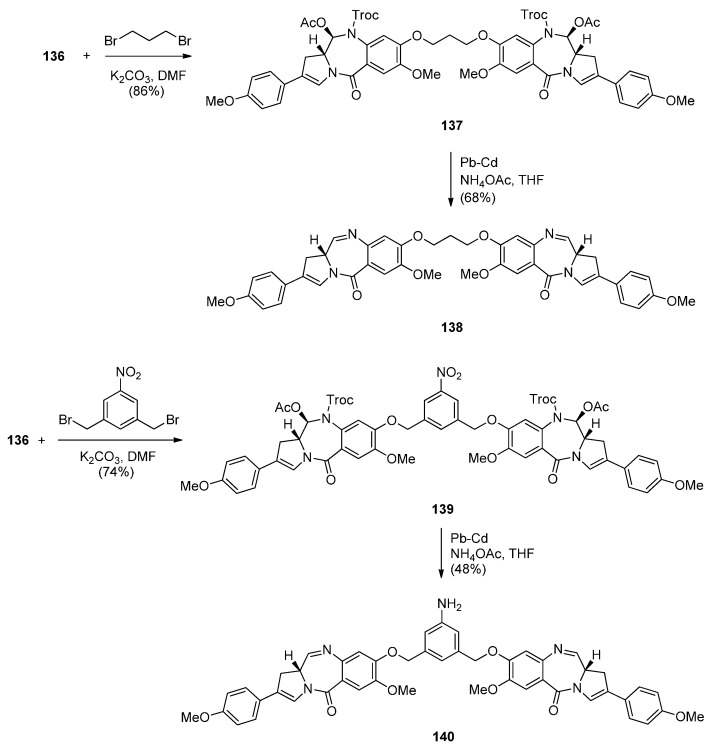
Synthesis of C8,C8′-linked PBD symmetric dimers **138** and **140**.

**Scheme 25:**
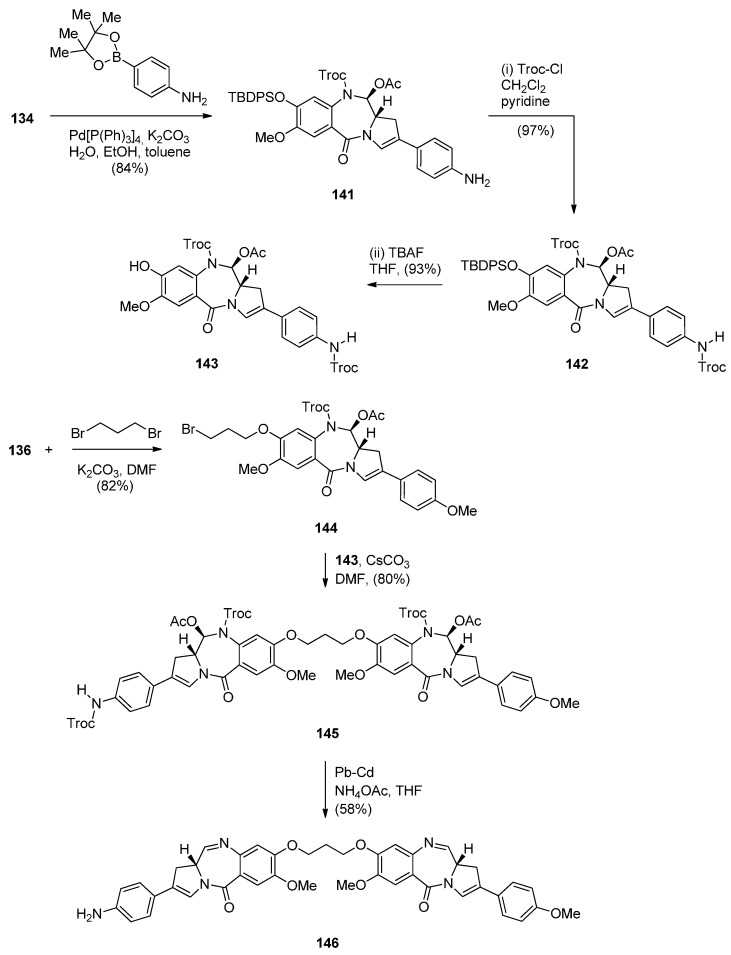
Synthesis of C8,C8′-linked PBD non-symmetric dimer **146**.

In view of the more potent cytotoxic activity of PBD dimers over PBD monomers and in order to better evaluate the properties of this class of therapeutic agents for ADCs (antibody–drug conjugates) for treating cancer, Kolakowski *et al.* [66] developed the versatile C2–aryl PBD monomer **136** (Scheme 22 and Scheme 23) that enables the late-stage diversification and synthesis of both symmetric and non-symmetric PBD dimers **137**–**140**, and, **145** and **146**, respectively (Scheme 24 and Scheme 25). This synthetic methodology is quite similar to the one presented by Thurston and co-workers [63] Scheme 17, [64] Scheme 19 and [65] Scheme 21, in that the PBD ring is constructed with either a 3-carboxypropyl substituent attached to the hydroxyl group at C8, *i.e.*, **101** or without a substituent at the C8–OH group, *i.e.*, **136**. In both cases the substitution at C8 is elaborated further while imine formation at N10–C11 of the PBD ring takes place in the last synthetic step. The synthesis of the key intermediate, PBD monomer **134** (Scheme 23), began by converting the readily available carboxylic acid **125** to the corresponding acid chloride and without isolation treated with TBS-protected pyrrolidine diol **126** to yield the amide **127**. Under catalytic hydrogenation the nitro group of **127** was reduced to amino and the phenol group deprotected to provide amino-phenol **128**. The 3-hydroxyl group of **128** was subjected to a Ley oxidation to give the corresponding ketone whose phenolic hydroxyl group reacted with *tert*-butyldiphenylsilyl chloride (TBDPSC) to afford protected **129**. Next **129** was protected by reaction with trichloroethyl chloroformate (Troc-Cl) to afford Troc-amine **130**. Selective deprotection of the TBS group of **130** gave primary alcohol **131**. Oxidative cyclisation of **131** to PBD lactol **132** was accomplished by the Swern oxidation.

In order to proceed to the synthesis of symmetrical and non-symmetrical PBD dimers key intermediate **136** (Scheme 23) was required. This was achieved by four synthetic steps from lactol **132**. Acetylation of the C11–OH of **132** to **133** was followed by enol triflation to compound **134** which underwent Suzuki coupling with 4-methoxyphenylboronic acid to give 2-aryl derivative **135**. Treatment of **135** with tetra–*n*-butylammonium fluoride (TBAF) removed the phenolic TBDPS group to yield the C8–OH PBD **136**.

The following four PBD dimers **137**–**140** were synthesized as shown in Scheme 24. The synthetic strategy, starting from compound **125** (Scheme 22), was focused on preparing the PBD monomer **136** with a C2 4-methoxyphenyl substituent required in the PBD dimer structures and a C8–OH group used for joining two PBD monomers with an appropriate linker, to form the PBD dimer. Thus, PBD dimer **137** was obtained by alkylating PBD monomer **136** with half an equivalent of 1,3-dibromopropane in base. Deprotection of the N10–Troc and C11–acetoxy groups of PBD **137** and concomitant formation of the N10–C11 imine group took place in a one-pot procedure utilizing palladium-cadmium couple to afford the well-studied C2 *p*-methoxyphenyl dimer SG2202, PBD **138**. Accordingly, dimerization of monomer **136** with 1,3-bis(bromomethyl)-5-nitrobenzene to PBD dimer **139** and then reduction, afforded the symmetrical PBD dimer **140**.

Non-symmetric dimers **145** and **146** (Scheme 25) were prepared essentially as the above symmetrical dimers. This time apart from PBD monomer **136** the other PBD is C8–OH C2 4-NHTroc protected monomer **143**. Troc protection is required to avoid alkylation of the aniline group when the two PBD monomers are connected by alkylation of their C8–OH groups. Thus, the synthetic strategy towards PBD dimer **145** (Scheme 25) requires Suzuki coupling of **134** with 4-aminophenylboronic acid pinacol ester, protection of the 4-aniline group of **141** as NHTroc derivative **142** and then deprotection of the *tert*-butyldiphenylsilyl (TBDPS) group of **142** to yield **143**. PBD monomers **136** and **143** are linked via 1,3-dibromopropane, as shown in Scheme 25, to yield the protected non-symmetric PBD dimer **145**. Deprotection of the latter compound by a lead-cadmium couple led to PBD imine dimer **146**.

##### Reductive Cyclisation of *N*-(2-Azidobenzoyl)pyrrolidine-2-Carboxaldehydes

**Scheme 26:**
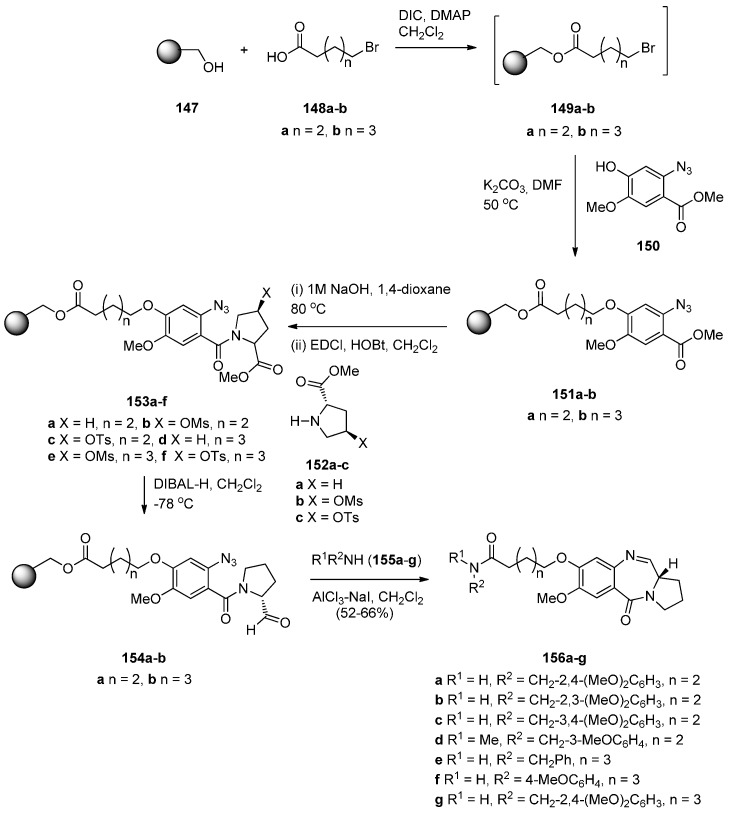
Synthesis of C8-*O*-substituted PBD conjugates **156a**–**g**.

The use of *N*-(2-azidobenzoyl)pyrrolidine-2-carboxaldehydes to obtain PBD N10–C11 imines was reported independently by Eguchi and Molina [10] in 1995. They used the Staudinger reaction on the azido aldehyde to form an iminophosphorane which spontaneously cyclised via an aza-Wittig reaction with the adjacent aldehyde group of the molecule. Kamal and co-workers introduced polymer-supported reagents for this methodology and also reported various methods of reducing the nitro group of *N*-(2-azidobenzoyl)pyrrolidine-2-carboxaldehydes to an amino group followed by cyclisation to the PBDs [10]. The latter method of cyclisation combined with the use of a polymer support was recently published by Kamal *et al.* [67] who prepared a library of tricyclic PBDs **156a**–**g** (Scheme 26). The synthetic approach used was first to couple the Wang resin **147** to the bromo-acids **148a**,**b** and without isolating resulting resin-bound esters **149a**, their reaction with methyl 2-azido-4-hydroxy-5-methoxybenzoate (**150**) [68] to provide the desired polymer supported ethers **151a**,**b**. The aromatic ester group of ethers **151a**,**b** was hydrolysed and subsequently coupled with substituted L-proline esters **152a**–**c** to provide the key resin-bound intermediates **153a**–**f**. The resin-bound azido esters **153a**,**b** were selectively reduced with diisobutylaluminium hydride (DIBAL-H) to their corresponding resin-bound azido-aldehydes **154a**,**b** and then submitted to a one-pot reaction of aluminium trichloride and sodium iodide assisted cleavage of the resin to the free acid, coupling with the added excess amount of amines **155a**–**g** and concomitant tandem selective azido-reductive cyclocondensation with the aldehyde group, to afford the desired final PBD conjugates **156a**–**g**, in 52%–66% yields. The synthetic method just described constitutes a novel route to C8–*O*-substituted PBD N10–C11 imine conjugates, employing the aluminium trichloride and sodium iodide system to execute four independent reactions under one roof, resin cleavage, amino and carboxylic acid coupling to amide, reduction of azide to amine and finally intramolecular condensation of amine and aldehyde to imine.

##### Cyclisation of *N*-(2-Azidobenzoyl)pyrrolidine-2-carboxaldehydes

**Scheme 27:**
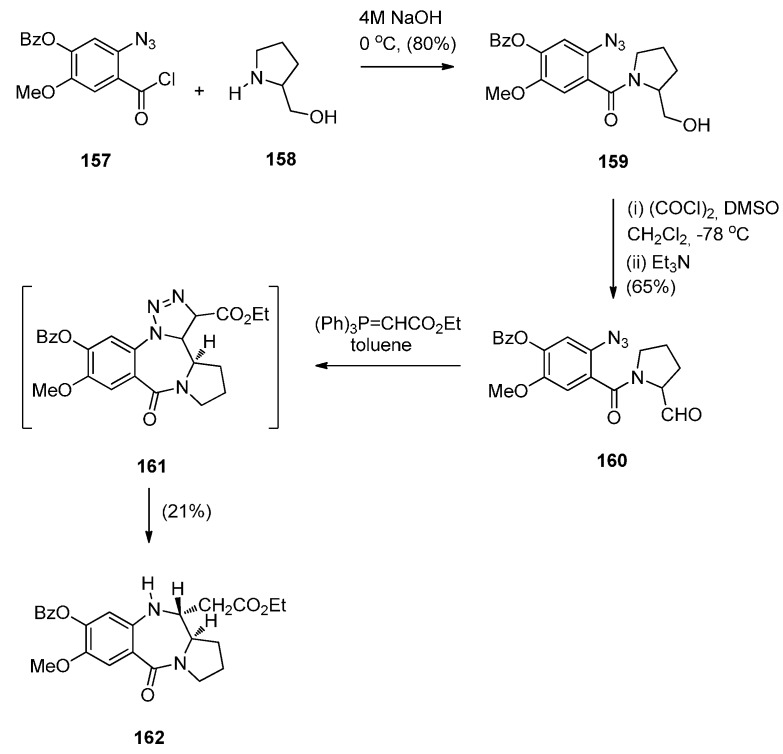
Synthesis of PBD **162**.

A rather intriguing but very low yielding approach to a PBD 5-one was described by Hemming *et al.* [69]. In the first step, the reaction involves acyl substitution between 2-azidobenzoyl chloride **157** and (*S*)-prolinol **158** to furnish amide **159** (Scheme 27) whose hydroxymethyl group was oxidised to afford, aldehyde **160** as a mixture of rotamers. Wittig reaction of this aldehyde with (carbethoxymethylene)triphenylphosphorane gave the corresponding α,β-unsaturated ester that underwent spontaneous intramolecular 1,3-dipolar cycloaddition, to give PBD **162** as a single diastereoisomer, in 21% yield. The authors assume that PBD **162** arises as a result of a free-radical based loss of nitrogen from intermediate triazoline **161** followed by hydrogen abstraction from the toluene solvent. Although the present synthetic route constitutes a novel method of preparing 11-ethoxycarbonylmethyl PBD 5-ones, the author has presented only one example and the route suffers from a very low yield in the final step.

**Scheme 28:**
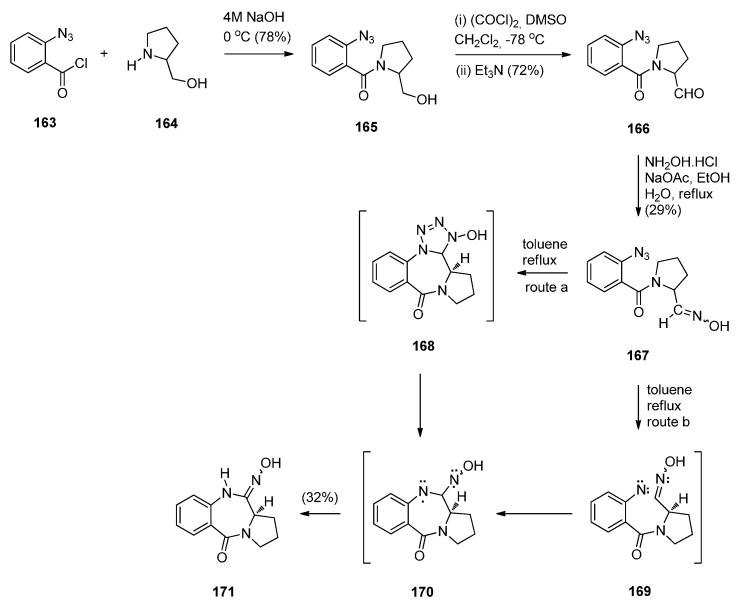
Synthesis of PBD **171**.

In the same year, Hemming *et al.* [70] reported the synthesis of azido-oxime **167** in order to study the potential of the azide to oxime intramolecular cycloaddition (Scheme 28). Acyl substitution of 2-azidobenzoyl chloride **163** by (*S*)-prolinol **164** afforded amide **165** that was subjected to Swern oxidation to yield azido-aldehyde **166**. Although up to this step the yields of products were over 70%, the oxime **167** was obtained in only 29% yield and PBD **171** was isolated in 30% yield. The formation of this product is consistent with either intramolecular 1,3-dipolar cycloaddition followed by nitrogen extrusion and diradical rearrangement (route a) or nitrogen extrusion followed by nitrene insertion and diradical rearrangement (route b). This synthetic route leading to a N10–OH PBD 11-oxime is another novel preparation of a PBD 5-one although only one product in low yield is described by the authors.

#### 2.1.2. Pyrrolo[2,1-*c*][1,4]benzodiazepine-5,11-Diones (Dilactams) with a Non-Aromatic Pyrrole Ring

The relatively strong interest in synthesing PBD dilactams stems from the fact that although they contain a non-electrophilic amidic functionality at the N10–C11 region, they are known to interact with DNA predominantly by hydrogen bonding [10]. There are five most commonly used methods to obtain these compounds, reductive cyclisation of alkyl *N*-(2-azido or nitrobenzoyl)-pyrrolidine-2-carboxylates, cyclocondensation of isatoic anhydrides with substituted prolines, and cyclisation of 1-benzoyl-*N*-methoxypyrrolidine-2-carboxamides and of *N*-(2-trifluoromethylsulfonyl-oxybenzoyl)-pyrrolidine-2-carboxylic esters [10]. Although the most prolific of these methods is reductive cyclisation of alkyl *N*-(2-nitrobenzoyl)pyrrolidine-2-carboxylates, the need in most cases for acid-catalysed cyclisation of the intermediate alkyl *N*-(2-aminobenzoyl)pyrrolidine-2-carboxylates can be avoided when methyl *N*-(2-azidobenzoyl)pyrrolidine-2-carboxylates are used instead. These azido ester reductive cyclisations leading to PBD lactams have been pioneered by Kamal and co-workers [10] who recently have reported another application of this method (Section “Reductive Cyclisation of Methyl *N*-(2-Azidobenzoyl)pyrrolidine-2-Carboxylates”). Another report leading to PBD dilactams uses the isatoic anhydride route (Section “Reaction of isatoic anhydrides with d-prolines”).

##### Reductive Cyclisation of Methyl *N*-(2-Azidobenzoyl)pyrrolidine-2-Carboxylates

**Scheme 29:**
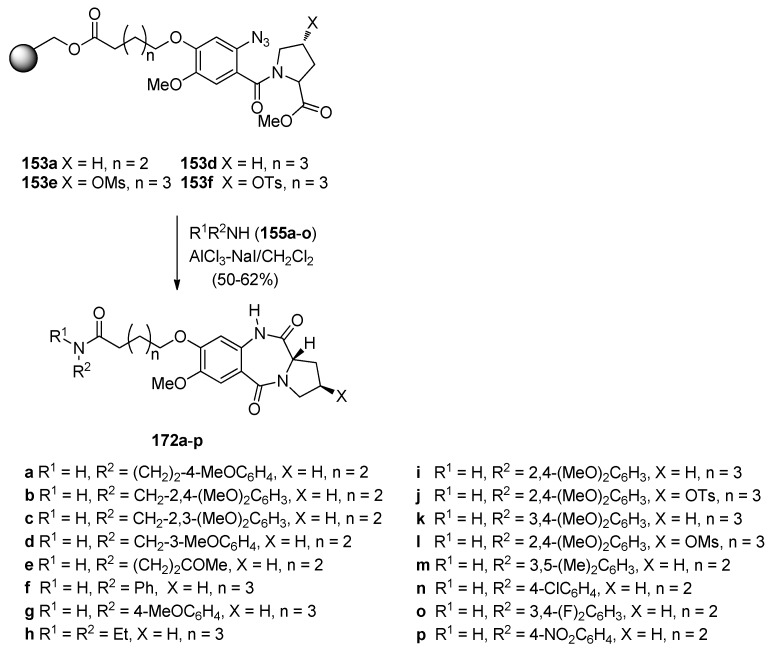
Synthesis of C8-*O*-substituted PBD conjugates **172a**–**p**.

By picking two of the key resin-bound methyl *N*-(2-azidobenzoyl)pyrrolidine-2-carboxylates **153a**,**d** described earlier (Section “Reductive cyclisation of *N*-(2-azidobenzoyl)pyrrolidine-2-carboxaldehydes”), Kamal *et al.* [67] used excess amines **155a**–**o** and applied the same cyclisation procedure (Scheme 29) as they had done for the cyclisation of *N*-(2-azidobenzoyl)pyrrolidine-2-carboxaldehydes **154a**,**b** (Section “Reductive Cyclisation of *N*-(2-azidobenzoyl)pyrrolidine-2-Carboxaldehydes”) and obtained the desired C8-substituted PBD-5,11-diones **172a**–**p**, in 50%–62% yields. The yields of this cyclisation reaction are much lower than the yields (80%–99%) of PBD lactams obtained from corresponding non-resin bound methyl *N*-(2-azidobenzoyl)pyrrolidine-2-carboxylates using a variety of reagents such as, hexamethyldisilathiane in methanol, trimethylsilyl chloride with sodium iodide in acetonitrile, ferrous sulfate heptahydrate in aqueous ammonia solution ferric chloride with sodium iodide, zinc and ammonium formate in methanol, aqueous hydriodic acid, sodium iodide in acetic acid, boron trifluoride diethyl etherate with ethanethiol or sodium iodide, and, boride in acidic methanolic media under microwave radiation [10].

##### Reaction of Isatoic Anhydrides with d-Prolines

The advantage of using isatoic anhydrides in the synthesis of PBD dilactam scaffolds is their convenient preparation, their high yielding condensation with L-proline, and also with *trans*-4-hydroxy-l-proline and l-glutamic acid, and the ease of purifying the PBD products. Condensation procedures typically involve heating with dimethyl sulfoxide (DMSO) at 100–120 °C, refluxing in *N*,*N*-dimethylformamide (DMF), heating without solvent at 150 °C and without solvent under microwave radiation where reaction times of 3 min have been recorded.

**Scheme 30:**
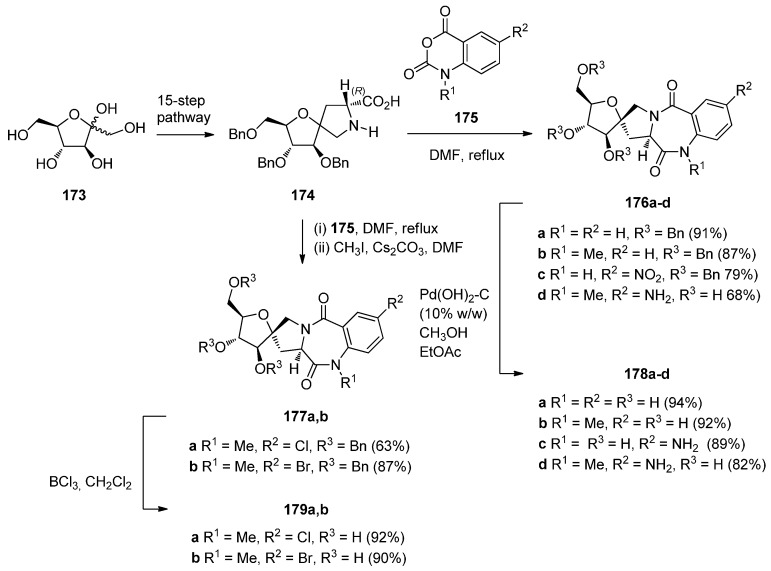
Synthesis of spiro PBD derivatives **176a**–**d**, **177a**,**b**, **178a**–**d** and **179a**,**b**.

Araújo *et al.* [71] have applied the isatoic anhydride route to synthesise a library of enantiomeric and conformationaly constrained PBDs dilactams spiro-linked to d- or l-fructose rings **176a**–**d**, **177a**,**b**, **178a**–**d** and **179a**,**b** (Scheme 30) which were then evaluated as GABAA receptor ligands. The starting material for this synthetic route is the bicyclic spiro d-proline analogue **174** prepared through a 15-step pathway from d-fructose **173** as described earlier [72]. Thus, d-proline analogue **174** was condensed with isatoic anhydrides **175** to afford PBDs **176a**–**d** with (11a*R*) configuration. Hydrogenolysis of PBDs **176a**–**d** with palladium (II) hydroxide on carbon resulted both in debenzylation and reduction of the nitro group, yielding the sugar-based PBDs **178a**–**d**. The synthesis of D-fructose-based PBDs **177a**,**b** was achieved by condensation of compound **174** with 5-chloro- or 5-bromoisatoic anhydride derivatives of **175**, followed by *N*-methylation. By using boron trichloride in dichloromethane debenzylation of PBDs **177a**,**b** occurred without removal of the halogen atoms to give PBDs **179a**,**b**. The yields of the PBD dilactams, obtained from condensing isatoic anhydrides and d- or l-prolines in boiling DMF are in the range 63%–91% whereas corresponding condensations in DMSO at around 110 °C or heating without solvent alone or under microwave radiation, are rarely under 80% [10].

#### 2.1.3. Pyrrolo[2,1-*c*][1,4]benzodiazepine-11-Ones with a Non-Aromatic Pyrrole Ring

##### Cyclodehydration of *N*-[2-(Aryl or Heteroaryl)hydroxymethyl]-l-Prolinamides

**Scheme 31:**
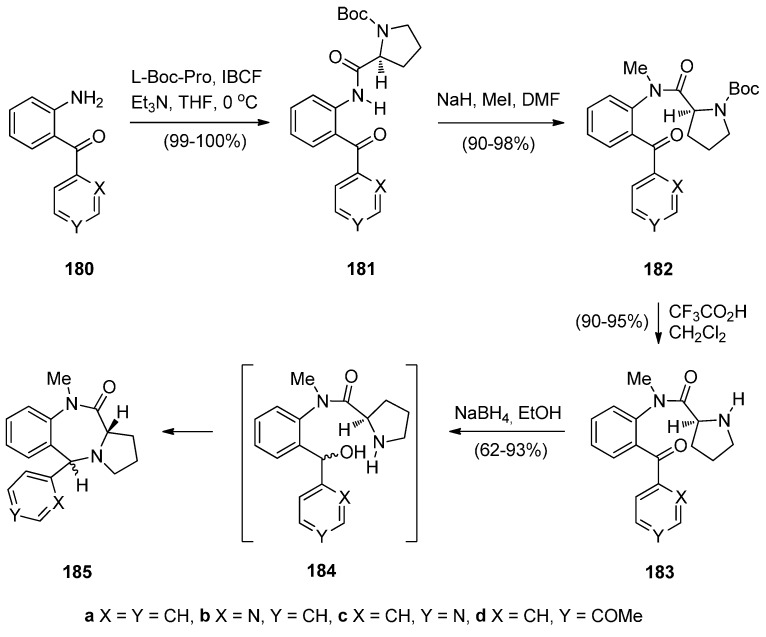
Synthesis of PBD derivatives **185a**–**d**.

Legerén and Domínguez [73] combined the structural features of 5-aryl-1,4-benzodiazepin-2-ones and pyrrolo[2,1-*c*][1,4]benzodiazepin-11-ones, both biologically versatile molecular families, and developed a new synthetic approach towards novel 5-arylpyrrolo[2,1-*c*][1,4]benzodiazepin-11-ones **185a**–**d** (Scheme 31) The starting materials for this synthesis, 2-aminophenylmethanones **180a**–**d**, were added to solution of l-Boc-proline and triethylamine that had been treated with isobutyl chloroformate (IBCF), to afford amides **181a**–**d** in virtually quantitative yield. Tertiary amides **182a**–**d** were prepared in excellent yields by methylation of amides **181a**–**d** which were then deprotected with trifluoroacetic acid (TFA) to amides **183**. HPLC of compounds **182a** and **183a** on chiral columns confirmed that the stereochemical integrity of the proline stereocentre is intact. Compounds **183a**–**d** were reduced to produce PBDs **185a**–**d** via cyclodehydration of the intermediate amidobenzhydrols **184a**–**d**. PBDs **185a**–**d** were isolated in the corresponding 63, 93, 62 and 68% yields and *trans*/*cis* diastereoisomer ratios 75/25, 90/10, 95/05 and 80/20, respectively.

#### 2.1.4. Pyrrolo[2,1-*c*][1,4]benzodiazepines with an Aromatic Pyrrole Ring

##### Cyclisation of *N*-(2-Aminobenzoyl)-1*H*-Pyrroles with Aldehydes.

**Scheme 32:**
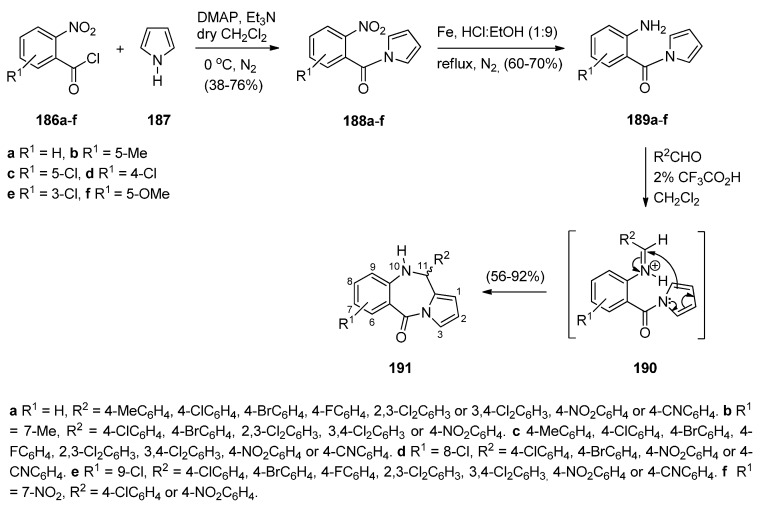
Synthesis of PBD derivatives **191a**–**f**.

Kundu and co-workers [74] synthesized a large number of PBDs **191a**–**f** (Scheme 32) with an aromatic pyrrole ring which are analogues of naturally occurring sibiromycin [75] and limazepine D [8]. In the first step of the synthetic route towards these compounds, 2-nitrobenzoyl chlorides **186a**–**f** reacted with 1*H*-pyrrole **187** to afford (2-nitrophenyl)pyrrol-1-ylmethanones **188a**–**f**. Reduction of the latter gave anilines **189a**–**f** which underwent cationic π-(7-*endo*) cyclisation with a variety of aromatic aldehydes and 2% trifluoroacetic acid as catalyst to afford PBDs **191**, with yields ranging from 56%–92%. In these reactions the intermediate electrophilic cationic iminium species is assumed to be intermediate **190**.

**Scheme 33:**
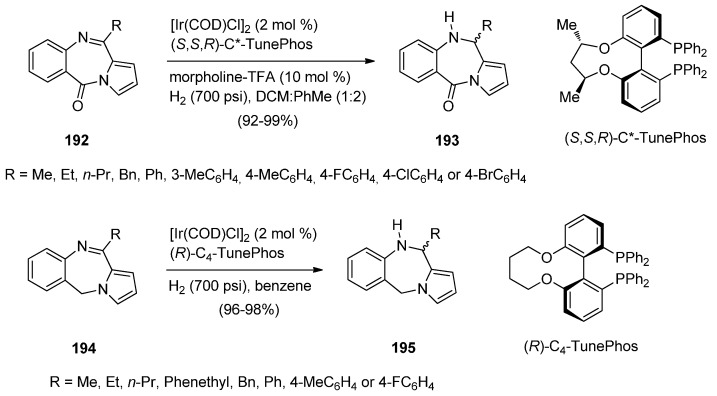
Synthesis of PBD derivatives **193** and **195**.

A novel and highly efficient Ir-catalysed asymmetric hydrogenation method for reducing the imine moiety contained in the seven-membered ring of 11-(alkyl or aryl)-5*H*-pyrrolo[2,1-*c*][1,4]-benzodiazepin-5-ones **193** and 11-(alkyl or aryl)-5*H*-pyrrolo[2,1-*c*][1,4]benzodiazepine **195** was developed by Zhou and co-workers [76] (Scheme 33). PBDs **193** and **195** were synthesised according to the procedures published by Kundu and co-workers [74] and Molinari *et al.* [77]. [Ir(COD)Cl]_2_ and (*S*,*S*,*R*)-C3*-TunePhos in a mixture of dichloromethane and toluene were added to the appropriate PBDs **192** and morpholine-trifluoroacetic acid and then hydrogenated at 700 psi of hydrogen to afford high yields of (+)-11-(alkyl or aryl)-10,11-dihydro-5*H*-PBDs **193** with 90%–96% ee. Similarly, but this time using [Ir(COD)Cl]_2_ and (*R*)-C_4_-TunePhos in benzene, the appropriate PBDs **194** were hydrogenated at 700 psi of hydrogen to afford high yields of (−)-11-(alkyl or aryl)-10,11-dihydro-5*H*-PBDs **195** with 82%–96% ee.

##### Cyclisation of 2-(*N*-Phthalimido)-*N*-[2-(1*H*-pyrrol-1-yl-methyl)phenyl]-Acetamide

**Scheme 34:**
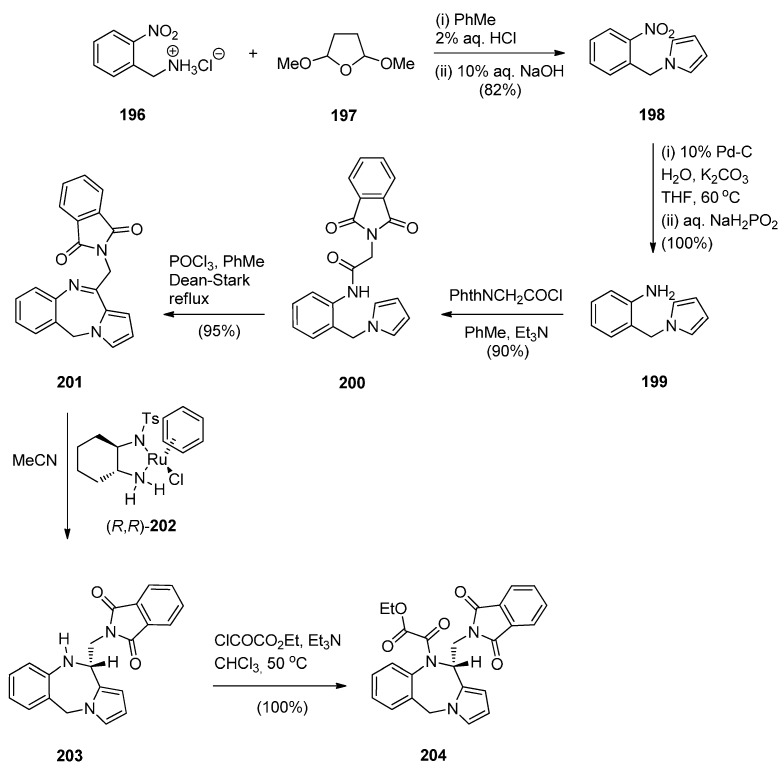
Synthesis of PBD derivatives **201**, **203** and **204**.

Three pyrrolo[2,1-*c*][1,4]benzodiazepines **201**, **203** and **204** were synthesised by Czarnocki *et al.* [78] en route to (2-methyl-1,3,4,14b-tetrahydro-2*H*,10*H*-pyrazino[1,2-*a*]pyrrolo[2,1-*c*][1,4]benzodiazepine (aptazepine), a potent α2-adrenoreceptor blocking agent. The synthetic pathway that we adopted is presented in Scheme 34 where commercially available 2-nitrobenzylamine hydrochloride (**196**) is condensed with 2,5-dimethoxy-tetrahydrofuran to 1-(2-nitrobenzyl)-1*H*-pyrrole (**198**) which was reduced and subsequently acylated with *N*-phthalylglycyl chloride to afford amide **200**. Conversion of this amide into the prochiral endocyclic imine **201** by a Bischler-Napieralski cyclisation and then asymmetric hydrogen-transfer using catalyst **202**, afforded PBD (*S*)-**203** with superior asymmetric induction (63% ee) and higher chemical yield (60%). Finally, amine **203** was reacted with ethyl chloro(oxo)acetate to afford amide (*S*)-**204** in quantitative yield. All the steps in this synthetic route gave yields that were over 80%.

#### 2.1.5. Pyrrolo[2,1-*c*][1,4]benzodiazepine-3-ones with a Non-Aromatic Pyrrole Ring

##### Reductive Cyclisation of *in Situ* Generated 1-(2-aminobenzyl)-5-aroylpyrrolidin-2-ones

**Scheme 35:**
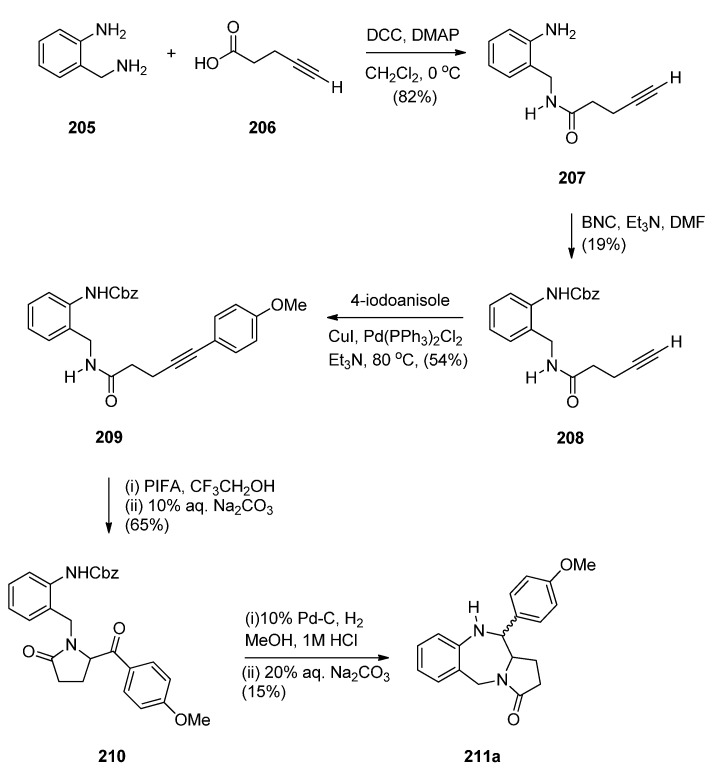
Synthesis of PBD derivative **211a**.

Domínguez and co-workers [79] synthesised PBD **211a** by a sequence of reactions starting from 2-(aminomethyl)aniline (**205**) (Scheme 35). The key step towards the synthesis of PBD **211a** is reductive cyclisation of amino-ketone **210** derived from transforming **205** with pent-4-ynoic acid (**206**) into amide **207**, protecting as carboxybenzyl derivative **208**, performing a Sonogashira coupling with 4-iodoanisole to give alkyne **209** and then [bis(trifluoroacetoxy)iodo]benzene (PIFA) mediated cyclisation to afford the pyrrolidinone **210**, in good yield. Catalytic hydrogenation of **210** in acidic methanol produced PBD **211a** in very low yield and poor diastereoselectivity (67/33, *syn*/*anti*), through a combination of three consecutive single processes, deprotection, condensation with the carbonyl group, and reduction of the resultant imine.

**Scheme 36:**
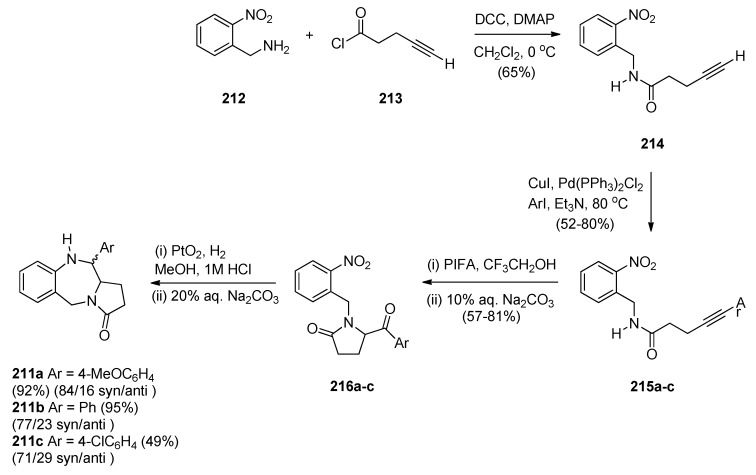
Synthesis of PBD derivatives **211a**–**c**.

The authors considered the above synthetic route to PBD **211a** quite unsatisfactory since it produced a low overall yield and poor diastereoselectivity in the final step. They therefore devised a new protection-free synthetic alternative to this compound and to two other derivatives **211b**,**c** (Scheme 36). The synthesis started from 2-(nitrobenzyl)amine (**212**) and followed a route similar to that depicted in Scheme 35. Thus, after subjecting **212** to successive steps of amidation that gave **214**, Sonogashira coupling that gave **215a** and PIFA-mediated intramolecular cyclisation that gave pyrrolidinone **216a**, the final reductive cyclisation step of **216a** into PBD **211a** was optimized by experimenting with different catalysts (10% by weight of PtO_2_, Ra-Ni, Pd(OH)_2_ or Pd-black, in methanol). The best conditions required the use of Adam’s catalyst (PtO_2_) under a hydrogen atmosphere at 70 psi. PBD **211a** was obtained in excellent yield with 84/16 *syn*/*anti* stereoselectivity. PBDs **216b**,**c** were prepared in a similar manner. Similar reductive cyclisation of **216b**,**c** into the corresponding PBDs afforded **211b** in excellent yield and **211c** in moderate yield while both compounds showed good overall diastereoselectivities.

### 2.2. Pyrrolo[1,2-a][1,4]benzodiazepines

A large number of [1,2-*a*] PBDs have been synthesised from the condensation of 1-(2-aminomethylphenyl)pyrroles with carbonyl compounds to provide non-isolable imines that undergo an intramolecular Mannich reaction, in acidic conditions, to form the diazepine ring (Section “Cyclisation of 1-(2-aminomethylphenyl)pyrrole with Carbonyl Compounds or a Hemiacetal”). Another frequently used method of synthesising [1,2-*a*] PBDs is reacting 1-(2-aminomethyl-phenyl)pyrroles with ethyl formate, carboxylic acid chlorides or anhydrides to form amides that are subjected to Bischler-Napieralski reaction conditions (Section “Cyclisation of Amide, Urea and Thiourea Derivatives of 1-(2-aminomethylphenyl)pyrrole”)

#### 2.2.1. Pyrrolo[1,2-*a*][1,4]benzodiazepines with an Aromatic Pyrrole Ring

The most characteristic features of pyrrolo[1,2-*a*][1,4]benzodiazepines in this category are: (i) all PBDs have an aromatic pyrrole ring; (ii) no carbonyl groups on the diazepine ring and the PBD N5 is a secondary amine; (iii) no carbonyl groups on the diazepine ring and the PBD C4–N5 is an imine; (iv) no carbonyl groups on the diazepine ring and the PBD has a thiocarbonyl group at C4 as a C4–C5 thiolactam; (v) no carbonyl groups on the diazepine ring and the PBD N5–C6 is an imine or (vi) carbonyl group on diazepine ring which is an N5–C6 lactam of the PBD.

##### Cyclisation of 1-(2-Aminomethylphenyl)pyrrole with Carbonyl Compounds or a Hemiacetal

**Scheme 37:**
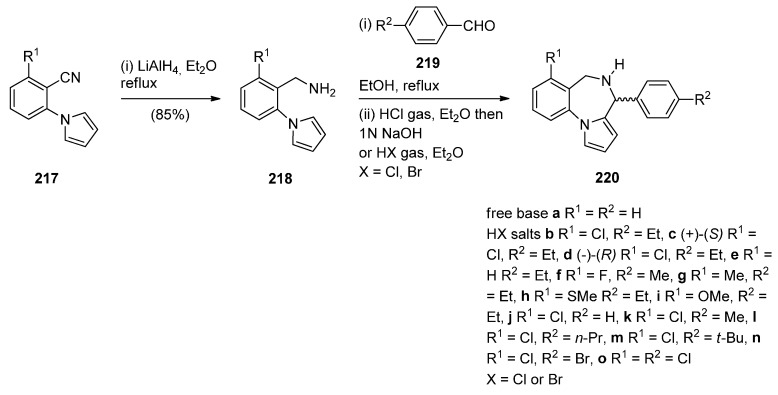
Synthesis of PBD derivatives **220a**–**o**.

The first synthesis of the pyrrolo[1,2-*a*][1,4]benzodiazepine ring system was reported by Cheeseman and Rafiq [2]. 1-(2-Cyanophenyl)pyrrole (**217a**, Scheme 37) was prepared by heating under reflux 2-aminobenzonitrile with 2,5-diethoxytetrahydrofuran in acetic acid via a modified Hantzsch pyrrole synthesis, was reduced to primary amine **218** with lithium aluminium hydride (LiAlH_4_). Condensation of amine **218** with benzaldehyde **219a** formed the corresponding imine which underwent a spontaneous intramolecular Mannich reaction to afford a racemic mixture of PBD **220a**. Meerpoel *et al.* [44] use this methodology in the search for novel anti-fungal agents and synthesised racemic PBD salts **220b**-**o**. Although in the former reaction the intramolecular Mannich step did not require the addition of a dry acid catalyst, in the latter reactions the starting materials were consumed faster during both the imine formation and the intramolecular step, by the addition of dry hydrochloric or hydrobromic acid in diethyl ether. Furthermore, PBD **220b** was separated into its (+)-(*S*) enantiomer **220c** and (−)-(*R*) enantiomer **220d** using chiral HPLC. The absolute configuration of these compounds was found by vibrational circular dichroism (VCD)-FTIR measurement and cancellation.

**Scheme 38:**
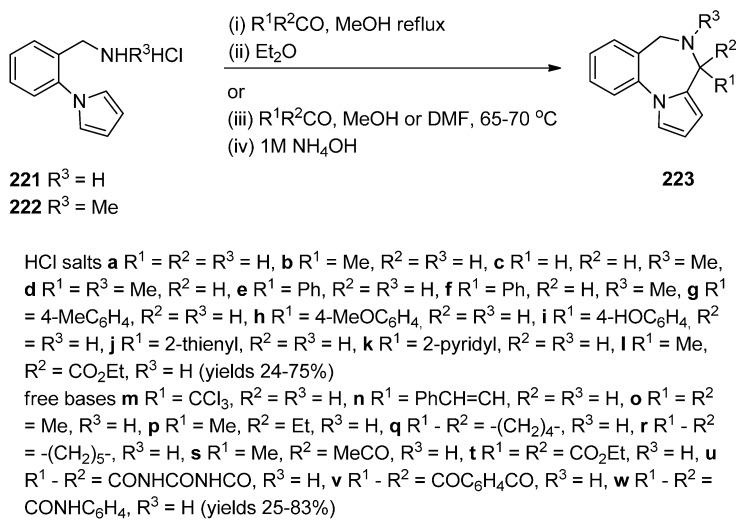
Synthesis of PBD derivatives **223a**–**w**.

Raines *et al.* [80] expanded on the initial work of Cheeseman and Rafiq [2] by reacting the aminomethyl hydrochloride **221** and **222** (Scheme 38) with appropriate carbonyl compounds in boiling methanol and then precipitating the PBDs **223a**–**l**, in poor to very good yields, by addition of diethyl ether. The carbonyl compounds used were aliphatic and aromatic aldehydes whereas one carbonyl compound was ethyl 2-oxopropanoate that yielded PBD **223l**. Cheeseman and Greenberg [81] continued their line of work [2] and also used the cyclocondensation of aminomethyl hydrochloride **221** (Scheme 38) with a variety of carbonyl compounds in either methanol or DMF at 65–70 °C followed by basification with ammonium hydroxide, in order to release the PBD cyclic secondary amines **223m**–**w**, in yields comparable to those obtained for compounds **223a**–**l**.

**Scheme 39:**
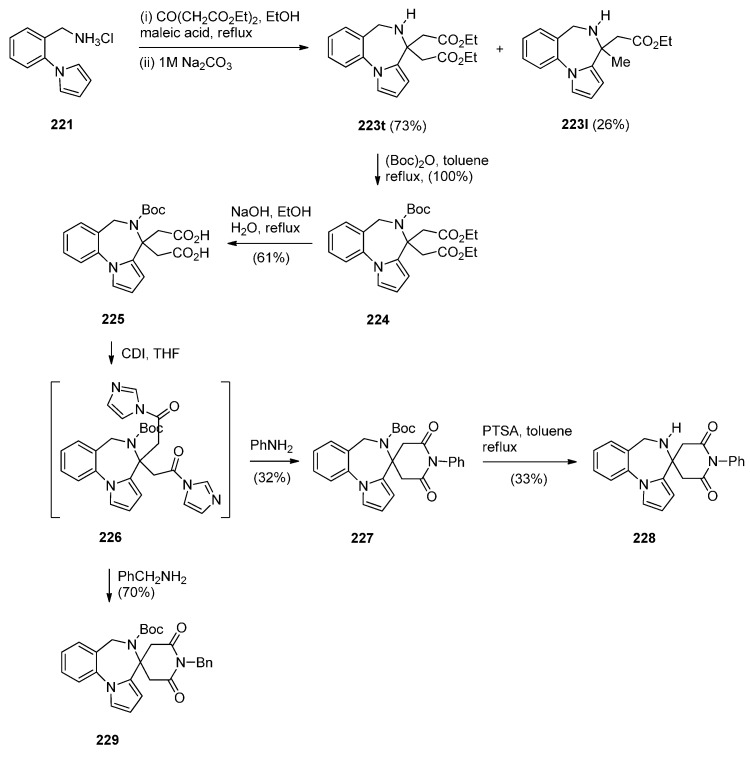
Synthesis of PBD derivatives **223**–**229**.

Artico and co-workers [82] became interested in PBDs due to their ongoing study on buspirone-like spiro derivatives and their desire to explore the usefulness 5,6-dihydro-4*H*-pyrrolo[1,2-*a*][1,4]benzodiazepine-4,4,-diacetic acid diethyl ester (**223t**, Scheme 39) as a scaffold for the synthesis of polycyclic nitrogen compounds of potential pharmacological interest. The target spirodioxopiperidine-PBDs **227**–**229** were synthesised from the PBD diester **223t**. This compound was formed together with PBD ester **223l** from the reaction of aminomethyl hydrochloride **221** with diethyl 1,3-acetonedicarboxylate. Reaction of PBD **223t** with di-*tert*-butyl dicarbonate (Boc_2_O) afforded Boc protected derivative **224**. The latter compound was hydrolysed to the PBD diacetic acid **225** and then reacted with 1,1′-carbonyldiimidazole (CDI) to form the intermediate PBD diamide **226**. Addition of aniline to the latter produced spirodioxopiperidine-PBD **227**. The benzyl derivative **229** was obtained similarly from intermediate **226** by adding benzylamine in the place of aniline. Deprotection of the Boc group of **227** yielded spirodioxopiperidine-PBD **228**.

**Scheme 40:**
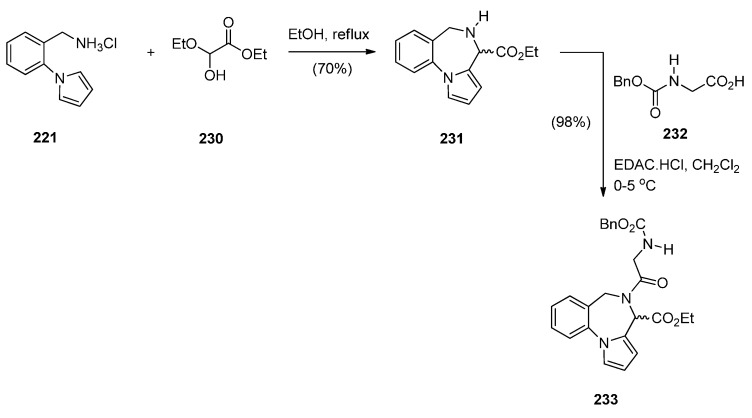
Synthesis of PBD derivatives **231** and **233**.

Massa, Corelli and co-workers [83] in search of new central nervous system agents with a pyrrole moiety and in particular pyrazino[2,1-*c*]pyrrolo[1,2-*a*][1,4]benzodiazepines, found out that the best route to synthesise these compounds is *via* PBDs **231** and **233** (Scheme 40). Therefore, Pictet-Spengler condensation of amine hydrochloride **221** with ethyl 2-ethoxy-2-hydroxyacetate (**230**) provided racemic PBD **231**. The secondary amino group of **231** was derivatised by reaction with benzyloxycarbonylglycine **232** in the presence of l-(3-dimethylaminopropyl)-3-ethylcarbodiimide hydrochloride (EDAC.HCl) to provide racemic PBD **233**, in excellent yield. The methyl ester of **231** was prepared analogously [46].

##### Cyclisation of Amide, Urea And Thiourea Derivatives of 1-(2-Aminomethylphenyl)pyrrole

**Scheme 41:**
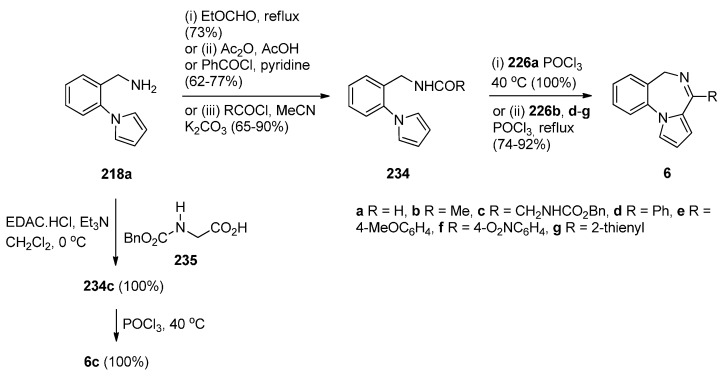
Synthesis of PBD derivatives **6a**–**g**.

Cheeseman and Rafiq [2] were the first to report the application of the Bischler–Napieralski reaction on amidomethyl derivatives **234b**,**d** to produce the corresponding PBD C4–N5 imines **6b**,**d** (Scheme 41). Amides **234b**,**d** were derived from aminomethyl derivative **218a** by reaction with acetic anhydride and benzoyl chloride, respectively. En route to the synthesis of tetracyclic nitrogen heterocycles as potential pharmacophoric structures for drug design, Massa *et al.* [84] prepared PBDs **6a**,**c** (Scheme 41). Both compounds were prepared from amine **218a**. The parent PBD **6a** required heating the amine with ethyl formate to give formamide **234c** and then cyclisation with phosphoryl chloride at 40 °C while PBD **6c** was prepared by coupling the amine with protected glycine **235** in the presence of 1-ethyl-3-(3-dimethylaminopropyl)carbodiimide hydrochloride (EDAC.HCl) to produce amide **234c** and then cyclisation using similar reaction conditions. In two recent publications Voskressensky *et al.* [85,86] used the same methodology to synthesise novel PBDs **6b**,**d**–**g** from amine **218a** via cyclisation of amides **234b**,**d**–**g**. The yields of the Bischler–Napieralski cyclisation step are noted to be from 74% with up to quantitative yield. Quantitative yields were obtained only when the precursors contained either *N*-methylformamide or *N*-methylacetamide groups and the reactions were heated in phosphoryl chloride at 40 °C. Heating under reflux in phosphoryl chloride is by far most frequently used for precursors containing *N*-methylbenzamide, *N*-methylarylamide and *N*-methylthiophene-2-carboxamide groups.

**Scheme 42:**
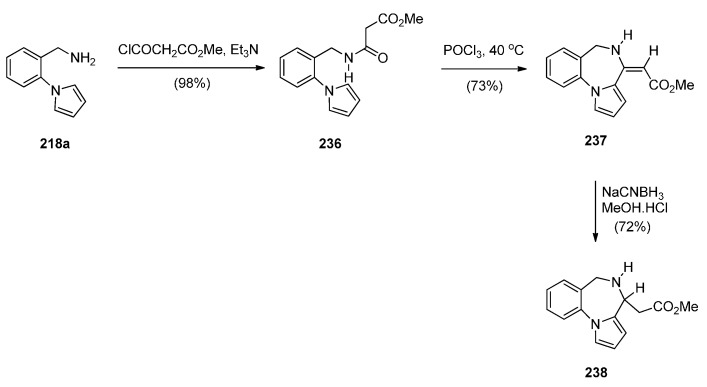
Synthesis of PBD derivatives **237** and **238**.

In the Section “Cyclisation of 1-(2-Aminomethylphenyl)pyrrole with Carbonyl Compounds or a Hemiacetal”, Artico and co-workers [82] synthesized the 4-methyl ethyl ester derivative of **238** (*i.e.*, **223l**) by an intramolecular Mannich reaction (Scheme 38). In the same paper he also presented a convenient synthetic route to PBD methyl ester **238**, unsubstituted at position 4 (Scheme 42). Following the isolation of acetamidopyrrole **236** in high yield from the reaction between and methyl malonyl chloride, the Bischler–Napieralski reaction provided PBD enamine **237**, after tautomerisation of the initially formed PBD C4–N5 imine. Compound **237** was then reduced with sodium cyanoborohydride to afford PBD **238**.

**Scheme 43:**
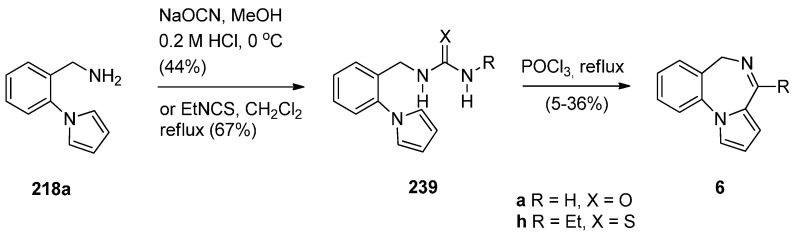
Synthesis of PBD derivatives **6a**,**h**.

Another method of synthesizing [1,2-*a*] PBD C4–N5 imines with an aromatic pyrrole ring is using Bischler–Napieralski reaction conditions on precursors such as **239** (Scheme 43) containing, instead of an amide function group, a urea or thiourea group. Duceppe and Gauthier [87] used this method to synthesize PBDs **6a**,**h** during an effort to prepare tricyclic compounds incorporating a pyrrole ring that could be of potential pharmacological interest. PBDs **6a**,**h** were prepared from amine **218a** in two steps by reaction of with sodium cyanate to give urea **239a** and reaction of **218a** with ethyl isothiocyanate to give thiourea **239h**. Heating **239a**,**h** with phosphoryl chloride provided parent PBD **6a** in only 5% yield and PBD **6h** in 36% yield.

##### Cyclisation of the Isothiocyanate Derivative of 1-(2-Aminomethyl-phenyl)pyrrole

**Scheme 44:**
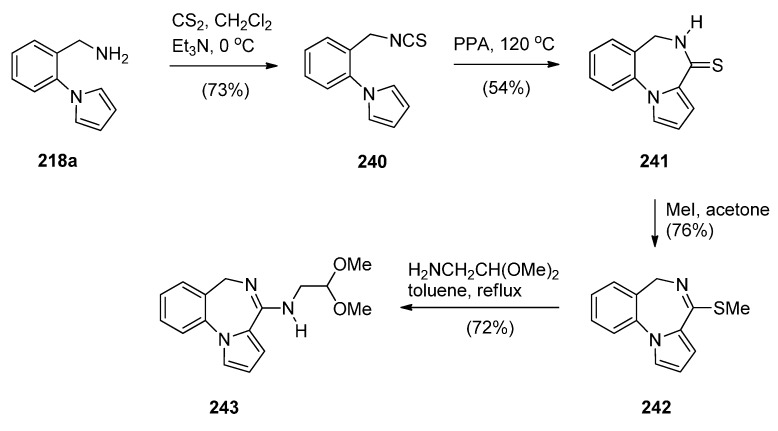
Synthesis of PBD derivatives **241**–**243**.

Duceppe and Gauthier [87] investigated yet another route to [1,2-*a*] PBD C4–N5 imines and this time targeted compound **242** (Scheme 44) whose 4-methylthio group was substituted by a primary amine, thus showing its potential in building a library of C4 substituted derivatives for biological evaluation. In the first step towards PBD **242** amine **218a** reacted with carbon disulfide followed by subsequent treatment of the resulting isothiocyanate **240** with polyphosphoric acid (PPA) at 120 °C. Thiolactam **241** was *S*-methylated with methyl iodide to afford C4–methylthio PBD **242** which was then reacted with 2-aminoacetaldehyde dimethylacetal to furnish amidoketal PBD **243**. The cyclisation of isothiocyanate **240** in polyphosphoric acid at 120 °C provided PBD thiolactam **241** in a mediocre 54% yield whereas the Bischler–Napieralski cyclisations of amides **234** and **236** (Scheme 41 and Scheme 42) provided PBDs **6** and **237**, in yields of 73%–100%.

##### Cyclocondensation of 2-Aminomethyl-1-(2-aroyl)pyrrole Derivatives

**Scheme 45:**
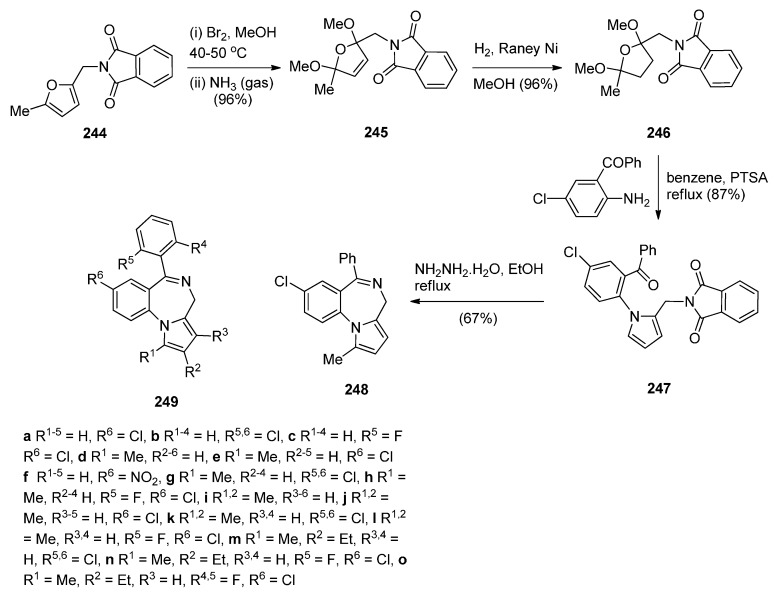
Synthesis of PBD derivatives **248** and **249a**–**o**.

Hara and co-workers [88] described a convenient high yielding synthesis of PBD N5–C6 imine **248** in four steps from 2-methyl-5-phthalimidomethylfuran **244** (Scheme 45). Bromination at the α-positions of the furan ring of compound **244** followed by substitution from methanol yielded 2,5-dimethoxydihydrofuran **245**. Compound **245** was obtained as a near 1:1 mixture of *trans*/*cis* isomers that were separated by fractional crystallisation. *Trans*-isomer **245** was then reduced by catalytic hydrogenation to the tetrahydrofuran **246**. Next, compound **246** was condensed with 2-amino-5-chlorobenzophenone, to afford pyrrolylbenzophenone **247**. The latter was heated with hydrazine hydrate causing cleavage of the phthaloyl group and cyclocondensation of the resulting intermediate aminoketone to yield PBD **248**. Hara *et al.* [45] continued to use this synthetic methodology and one year later presented the synthesis of fifteen more examples of this tricyclic ring, that is PBDs **249a**–**o**.

**Scheme 46:**
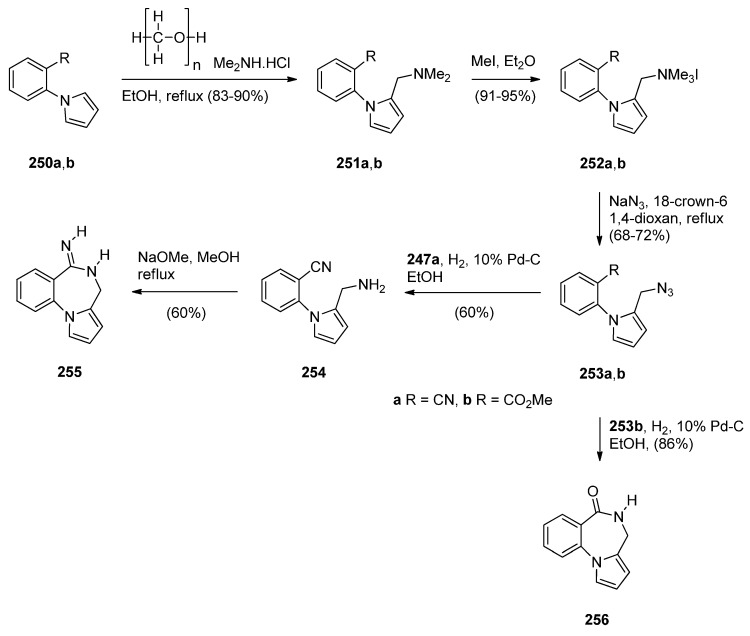
Synthesis of PBD derivatives **255** and **256**.

Within the framework of their scientific interests regarding the synthesis of nitrogen containing tricyclic compounds of potential pharmaceutical value, Korakas and Varvounis [89,90] presented the preparation of PBDs **255** and **256** from 1-arylpyrroles **250a**,**b** (Scheme 46). In the first step, pyrroles **250a**,**b** were subjected to Mannich reaction conditions to afford the respective dimethylamino-methylpyrroles **251a**,**b** which were treated with methyl iodide to produce the corresponding quaternary ammonium salts **252a**,**b**. Displacement of triethylamine from quaternary salts **252a**,**b** by azide anion afforded azides **253a**,**b** that were reduced by catalytic hydrogenation to afford, in the case of nitrile **253a**, aminomethylpyrrole **254** whereas in the case of ester **253b**, intermediate 2-aminomethyl-1-(2-methoxycarbonylphenyl)pyrrole reacted further by intramolecular acyl substitution, to yield PBD **256**. Upon heating aminonitrile **254** in methanolic sodium methoxide, intramolecular nucleophilic addition occurred to produce PBD 6-one **255**.

##### Cyclocondensation of *N*-(2,5-Dicarbonylalkyl)-2-nitrobenzamide Precursors Then Formation of Pyrrole Ring

**Scheme 47:**
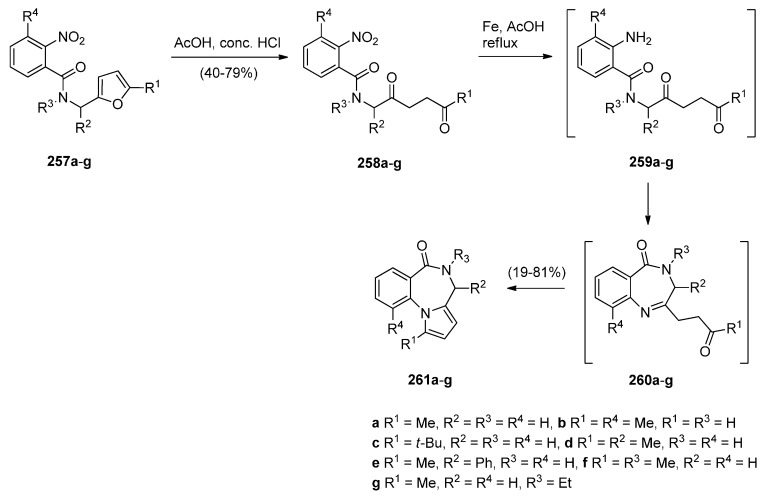
Synthesis of PBD derivatives **261a**–**g**.

Based on the importance of pyrrolo[1,2-*a*][1,4]benzodiazepines as therapeutic molecules, Butin *et al.* [91] implemented a new method to synthesise PBDs **261a**–**g** (Scheme 47), that consists of acid-catalyzed furan ring opening of *N*-(furfuryl)-2-nitrobenzamides **257a**–**g** followed by a *one pot* reduction of the nitro group to amino and subsequent cyclisation with concomitant formation of the diazepine and pyrrole rings. Hence, at first the efficiency of the furan ring-opening reaction was tested with various acids and it was found that furans **257a**–**g** treated with a mixture of glacial acetic acid and concentrated hydrochloric acid afforded nitrodiketones **261a**–**g** in yields ranging from 40%–79%. Reduction of these compounds in glacial acetic with iron powder produced non-isolable amines **259a**–**g** that cyclocondensed spontaneously to afford intermediate benzodiazepines **260a**–**g** which underwent another cyclocondensation to afford PBDs **261a**–**g**. With the exception of **261c**, obtained in only 19% yield due to steric hinderance from the *t*-Bu group, PBDs **261a**,**b**,**d**–**g** were isolated in very good yields.

#### 2.2.2. Pyrrolo[1,2-*a*][1,4]benzodiazepines with a Non-Aromatic Pyrrole Ring

The most characteristic features of [1,2-*a*] PBDs in this category are: (i) non-aromatic pyrrole ring in all PBDs; (ii) one carbonyl group on the diazepine ring either at positions 4 or 6 of the PBD, N atoms are at position 5 are substituted part of either C4–N5 lactams or N5–C6 lactams, or; (iii) no carbonyl groups on the diazepine ring and the PBD N5 is a secondary amine.

##### Cyclisation of a 2-Fluoro-*N*-(pyrrolidin-2-ylmethyl)benzamide Precursor

**Scheme 48:**
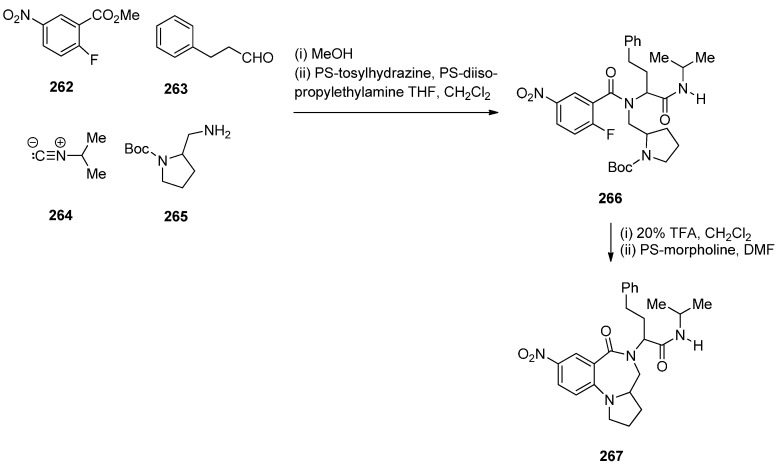
Synthesis of PBD **267**.

Tempest, Hulme and co-workers [92] developed a modified Ugi-4-component reaction to synthesise a series of biologically useful polycyclic heterocyclic cores. The synthesis of PBDs **267** (Scheme 48) by this method involves reacting 2-fluoro-5-nitrobenzoic acid (**262**) with 3-phenyl-propanal (**263**), isopropyl isocyanide (**264**) and *tert*-butyl 2-(aminomethyl)pyrrolidine-1-carboxylate (**265**), followed by the addition of polymer-bound PS-tosylhydrazine and PS-diisopropylethylamine, to afford Ugi product **266**, in good yield. Boc-deprotection of **266** by 20% trifluoroacetic acid (TFA) in dichloromethane and intramolecular S_N_Ar by subsequent addition of a proton scavenger such as polymer-bound PS-morpholine in DMF, produced PBD **267**, in very good yield.

##### Rearrangement of Intermediate 3,4′-Dioxospiro[pyrrolidine-1,1′-quinazoline]ylides

**Scheme 49:**
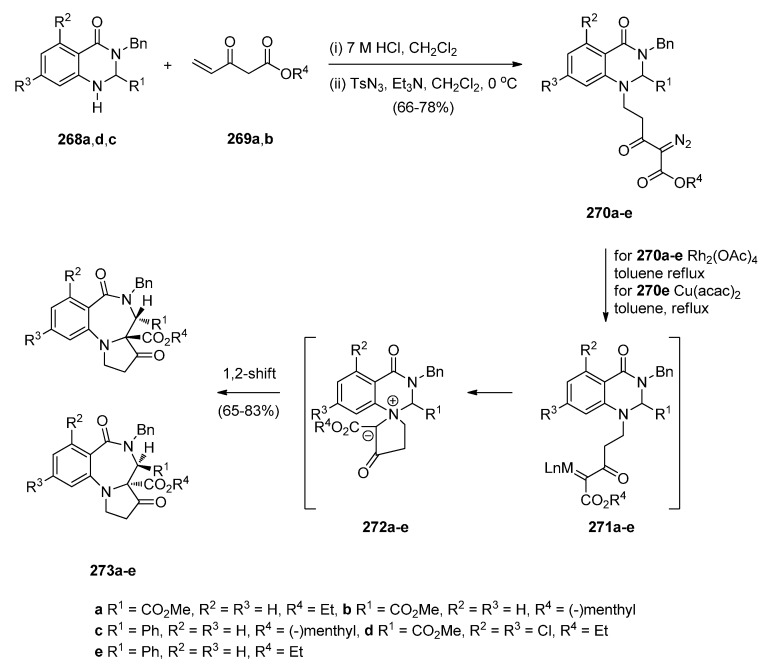
Synthesis of PBD derivatives **273a**–**e**.

Continuing their work on the synthesis of enantiopure bicyclic and tricyclic alkaloids, Saba and co-workers [93] describe the synthesis of PBDs **273a**–**e** (Scheme 49) by using the metallo carbenoid/spiro-[6,5]-ammonium ylide/Stevens[1,2]-shift with a ring-expansion sequence. The reaction sequence starts by the conjugate addition of (±)-methyl 4-oxo-1,2,3,4-tetrahydroquinazoline-2-carboxylates **268a**,**d** or (±)-3-benzyl-2-phenyl-2,3-dihydroquinazolin-4(1*H*)-one **268c**, to the appropriate ethyl-3-ketopent-4-enoate **269a** or (–)-menthyl-3-ketopent-4-enoate dichloromethane **269b** in dichloromethane containing hydrochloric acid, followed by diazo group transfer reaction with tosyl azide. The corresponding diazo compounds **270a**–**e** were isolated in good yields. Rhodium(II) acetate dimer [Rh_2_(OAc)_4_] catalyzed diazo-decomposition of **270a**–**d** was performed in refluxing toluene and it took forty minutes to give the PBDs **273a**–**d** as the sole products and in very good yields. For **270e** the reaction worked equally well when copper(II) acetylacetonate [Cu(acac)_2_] was used as catalyst, requiring only a few minutes boiling in toluene. PBD **273e** was isolated in 80% yield. It is expected that intermediate spiro-[6,5]-ammonium ylides **272**, that are formed via the nitrogen trapping of the metallo carbenoid group correctly situated on the pendant of intermediate **271**, undergo sigmatropic rearrangement to furnish the PBDs **273a**–**d**. Compounds **273a**,**d** and **e** were isolated as single diastereoisomers with trans configuration the substituents at the C-3a and C-4 positions whereas the chiral (–)-menthyl substituted derivatives **273b** and **273c** were obtained as a 1:1 mixture of trans diastereomers that could not be separated by column chromatography.

**Scheme 50:**
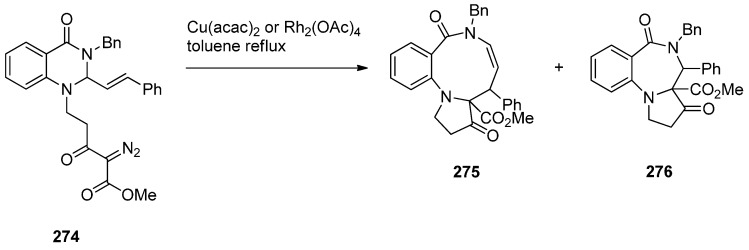
Synthesis of PBD **276**.

Two years later Saba and co-workers [94] prepared diazoquinazolinone **274**, using similar methodology as explained above (Scheme 49), to find out that the copper(II) acetylacetonate [Cu(acac)_2_] catalysed diazo decomposition of **274** (Scheme 50) gave a mixture of the pyrrolobenzodiazonine **275** and PBD **276**, in a 81:19 ratio. The same reaction using rhodium(II) acetate dimer [Rh_2_(OAc)_4_] afforded a mixture of **275** and PBD **276** in a 54:46 ratio. In both reactions the decompositions gave quantitative overall yields.

**Scheme 51:**
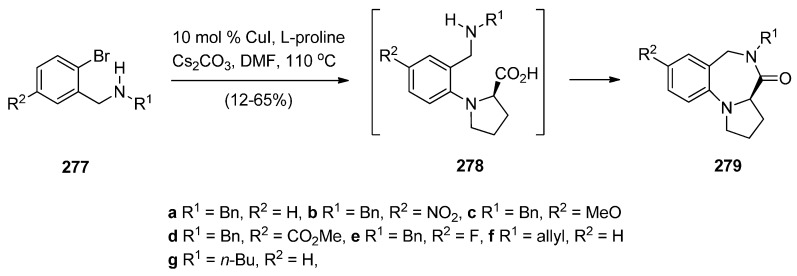
Synthesis of PBD derivatives **249a**–**g**.

There are numerous substituted 1,4-benzodiazepin-3-ones that exhibit useful biological activity. Gao, Ma and co-workers [95] have recently shown interest in the chemistry of amino acid promoted copper iodide-catalysed C–N bond formation and used this method to synthesise PBDs **279a**–**g** (Scheme 51). Thus, of *N*-(benzyl, allyl or *n*-butyl)-2-bromobenzylamines **277a**–**g** were coupled with l-proline using copper iodide and cesium carbonate to give first the intermediate coupling products **278a**–**g** which underwent intramolecular coupling to yield PBDs **279a**–**g**. It was found the electronic-rich substrate **277c** provided low yield (12%) of PBD **279c**.

##### Reductive Cyclisation of 1-[2-(Aminomethyl)phenyl]-5-(4-methoxybenzoyl)pyrrolidin-2-one

**Scheme 52:**
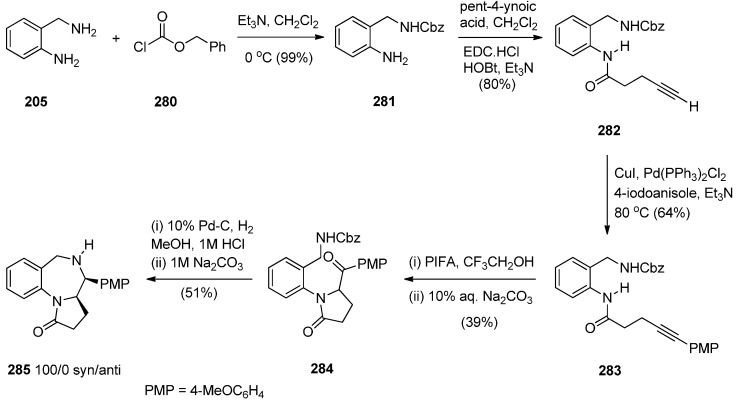
Synthesis of PBD **285**.

Domínguez and co-workers [79], (Section “Reductive cyclisation of *in situ* generated 1-(2-aminobenzyl)-5-aroylpyrrolidin-2-ones”), have also reported the synthesis of 4-(4-methoxyphenyl) PBD 1-one **285** (Scheme 52). The synthetic route starts by protecting selectively 2-(aminomethyl)aniline (**205**) as carbamate **281**. Coupling **281** with pent-4-ynoic acid using 1-ethyl-3-(3-dimethylaminopropyl)carbodiimide hydrochloride (EDC.HCl) and *N*-hydroxybenzotriazole (HOBt) afforded amide **282**. Next, Sonogashira coupling between alkyne group of **282** and 4-iodoanisole under standard reaction conditions, rendered 4-pentyamide **283**. Formation of the pyrrolidinone ring of **284** required (bis(trifluoroacetoxy)iodo)benzene (PIFA)-mediated cyclisation of **283** followed by basic hydrolysis. Catalytic hydrogenation of **284** with 10% palladium-on-carbon in acidic media caused successive deprotection, cyclodehydration of the ensuing aminoketone and reduction of the resulting cyclic imine to give the *syn*-diastereomer **285**, exclusively. The overall yield of PBD **285** is 10%.

### 2.3. Pyrrolo[1,2-d][1,4]benzodiazepines

#### 2.3.1. Pyrrolo[1,2-*d*][1,4]benzodiazepines with a Non-Aromatic Pyrrole Ring

There are only five examples of [1,2-*d*] PBDs where: (i) all have a saturated pyrrole ring with a carbonyl group adjacent the N atom; (ii) two compounds have a carbonyl group on the diazepine ring which forms a C6–N7 lactam of the PBD and (iii) three compounds are without a carbonyl group on the diazepine ring and the PBD has an N7 tertiary amine.

##### Rearrangement of Pyrrolo[2,1-*a*]isoquinolinediones

**Scheme 53:**
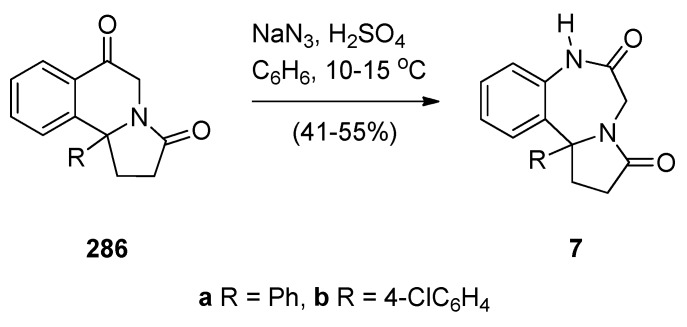
Synthesis of PBD derivatives **7a**,**b**.

The first two examples of [1,2-*d*] PBDs **7a**,**b** (Scheme 53) were reported in 1977 by Saito and co-workers [3]. Dihydropyrrolo[2,1-*a*]isoquinolinediones **286a**,**b** were subjected to Schmidt reaction conditions, namely sodium azide and sulphuric acid in benzene at low temperature to yield PBDs **7a**,**b** in moderate yields.

##### Cyclisation of 1-(2-Anilinoethyl)-5-ethoxypyrrolidin-2-ones

**Scheme 54:**
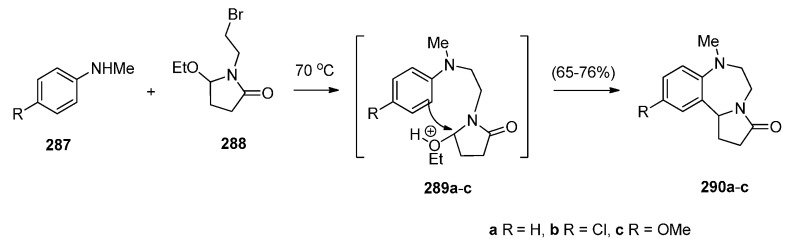
Synthesis of PBD derivatives **290a**–**c**.

Kraus and Yue [96] became interested in synthesizing PBDs **290a**–**c** (Scheme 54) due to their work on intermolecular aminoalkylations on aromatic rings. They used as starting material 1-(2-bromoethyl)-5-ethoxypyrrolidin-2-one **288** which was heated with the appropriate anilines **287a**–**c** at 70 °C without solvent to give the non-isolable alkylated anilines **289a**–**c**, that underwent spontaneous intramolecular substitution of the protonated ethoxy group by the aniline C-2, to afford PBDs **290a**–**c** in very good yields.

#### 2.3.2. Pyrrolo[1,2-*d*][1,4]benzodiazepines with an Aromatic Pyrrole Ring

There are thirty-eight examples of [1,2-*d*] PBDs where: (i) all PBDs have an aromatic pyrrole ring, (ii) thirty-three compounds have a carbonyl group on the diazepine ring which is an C6-N7 lactam of the PBD and (iii) five compounds have a carbonyl group on the diazepine ring which is a C6-N7 N-substituted lactam of the PBD.

##### Reductive Cyclisation of [2-Methyl-5-(2-nitrophenyl)pyrrol-1-yl]acetic Acids

**Scheme 55:**
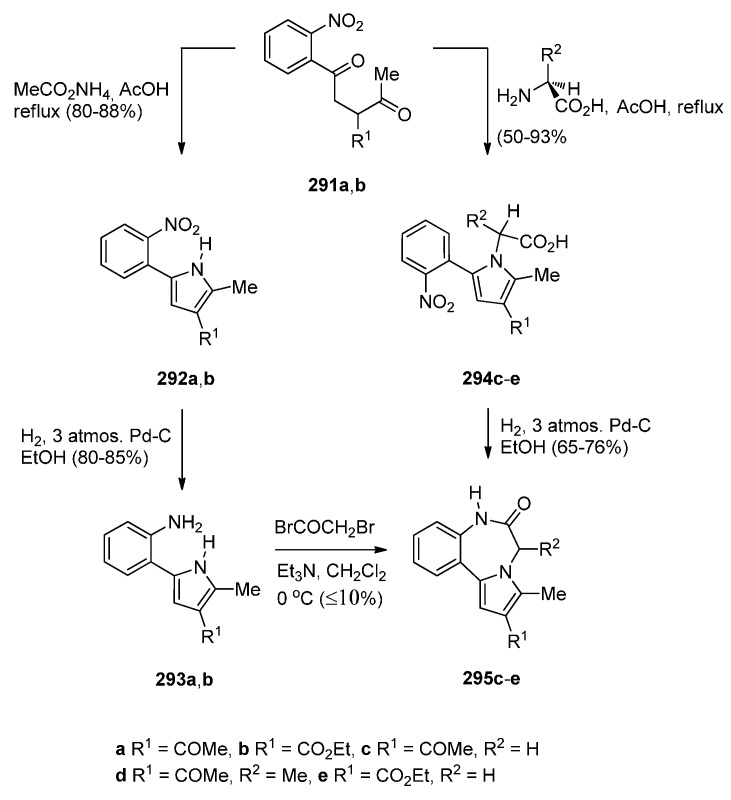
Synthesis of PBD derivatives **295c**–**e**.

The synthesis of PBDs **295c**–**e** from diketones **291a**,**b** has been reported by Aiello *et al.* [97] (Scheme 55). Thus, 3-acetyl-1-(2-nitrophenyl)pentane-1,4-dione **291a** or ethyl 2-acetyl-4-(2-nitro-phenyl)-4-oxo-butanoate **291a**,**b** were reacted with ammonium acetate on one hand and either glycine or alanine in the other, to produce 1-(2-nitrophenyl)pyrroles **292a**,**b** and **294c**–**e**, respectively. Reduction of pyrroles **292a**,**b** by catalytic hydrogenation afforded amino pyrroles **293a**,**b**. It was found that cyclisation of **293a**,**b** with bromoacetyl bromide was best performed with triethylamine in dichloromethane at low temperature but even after column chromatography PBDs **295c**,**e** were isolated in less than 10% yield. This problem was overcome by the shorter and more efficient route whereby the nitro acids **294c**–**e** were submitted to catalytic hydrogenation which caused reduction of the nitro group to amino and spontaneous cyclocondensation to yield PBDs **295c**–**e**, in very good yields. The by-products of the reaction between amino pyrroles **293a**,**b** and bromoacetyl bromide were identified in a later publication [98].

##### Cyclisation of *N*-(2-Carbonylphenyl)glycinamide Precursors Then Formation of Pyrrole Ring

**Scheme 56:**
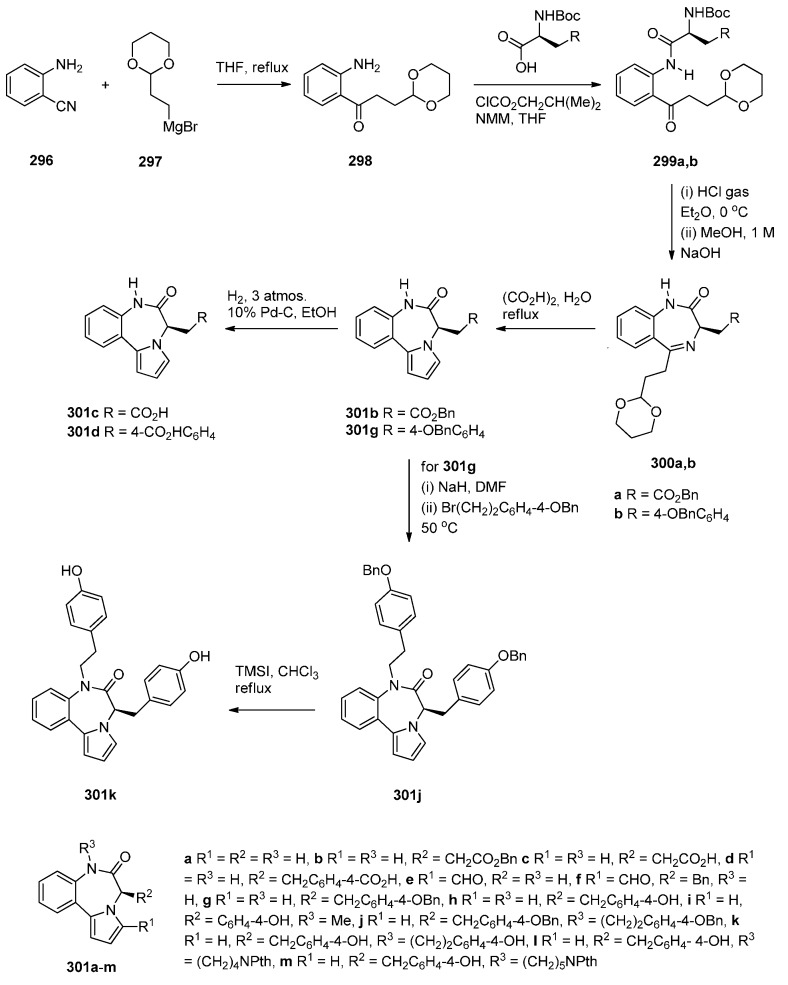
Synthesis of PBD derivatives **248** and **249a**–**o**.

Lucca and Otto [48] became aware from studies conducted by the Janssen Pharmaceutical Company that certain benzodiazepine derivatives synthesized for a peptidomimetics program could have antiviral properties against HIV-I. One of these compounds was PBD **301b** and in this context a large number of analogues **301a**–**m** were synthesized and tested. Selected syntheses are presented for PBDs **301b**,**c**,**d**,**g**,**j**,**k** in Scheme 56. The first step of the synthesis utilizes an excess of Grignard reagent **297** on anthranilonitrile **296** to give aminoketone **298** which is then coupled with either *N*-α-Boc-d-aspartic acid β-benzyl ester or Boc-*O*-benzyl-l-tyrosine in the presence of isobutyl chloroformate and *N*-methylmorpholine (NMM), to provide amides **299a**,**b**. Next, N-Boc deprotection and cyclocondensation produces benzodiazepines **300a**,**b** which upon heating in an aqueous solution of oxalic acid caused cyclisation into PBDs **301b**,**g**. Debenzylation by catalytic hydrogenation yielded PBD carboxylic acids **301c**,**d**. Furthermore, PBD **301g** was *N*-alkylated to PBD **301j** and by deprotecting both benzyl groups of this compound with trimethylsilyl iodide (TMSI), diphenolic PBD **301k** was obtained. The authors refer to the use of standard literature procedures and do not report the yields of the synthesized compounds. The results of the antiviral activity tests of compounds **301a**–**m** showed that compound **301b** is the most active with an IC_90_ of 0.29 μg/mL.

**Scheme 57:**
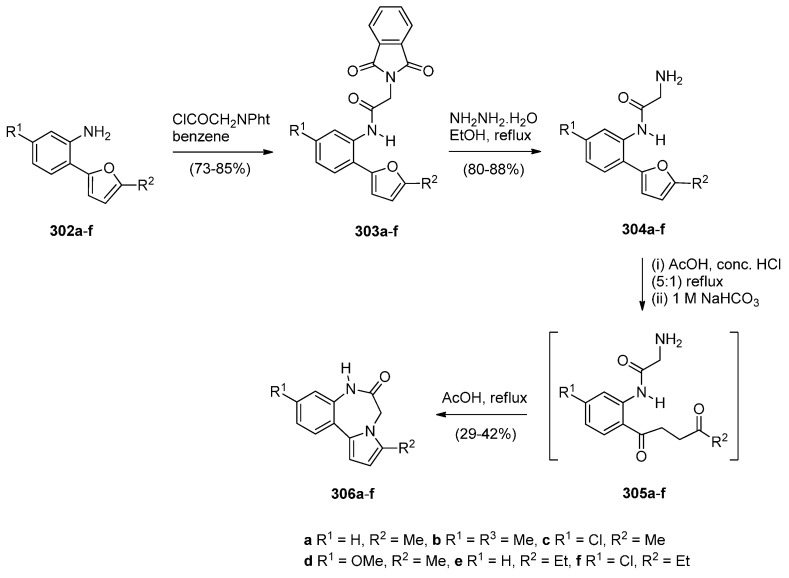
Synthesis of PBD derivatives **306a**–**f**.

Butin *et al.* [99] applied the same principle of an earlier method of synthesizing pyrrolo[1,2-*a*][1,4]benzodiazepines [91] from furfuryl derivatives in order to synthesize pyrrolo[1,2-*d*][1,4]benzodiazepines **306a**–**f** (Scheme 57). The starting materials for this synthesis are the 2-(2-aminoaryl)furans **302a**–**f** which were reacted with 2-(phthalimido)acetyl chloride in benzene to afford amides **303a**–**f**. Hydrazinolysis of the phthalamido ring of **303a**–**f** produced the corresponding amines **304a**–**f** which were heated in a mixture of acetic acid and hydrochloric acid causing ring opening of the furan ring to afford the intermediate crude aminodiketones **305a**–**f**. These were heated in glacial acetic acid causing two consecutive cyclodehydrations to afford PBDs **306a**–**f** in low yields. One reason for the low yields of the PBDs is incomplete conversion of furans **304** into intermediate diketones **305** which is possibly due to the conjugation between furan and benzene rings.

**Scheme 58:**
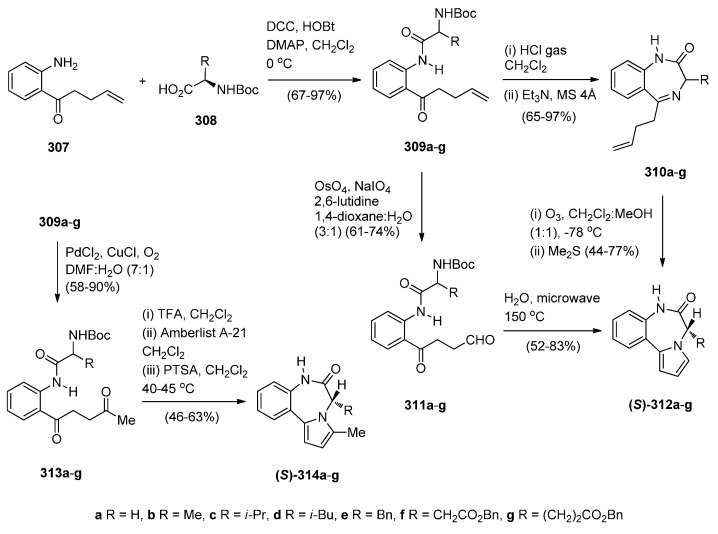
Synthesis of PBD derivatives **312a**–**g** and **314a**–**g**.

1,4-Diazepin-2-ones are considered important synthetic targets because they have shown therapeutic potential against inflammation and autoimmune diseases and have served as receptor ligands and enzyme inhibitors. They also have the ability to mimic natural γ- and β-turn peptide secondary structure. In order to investigate novel diazepinone analogues as potential γ-turn mimics, Dörr and Lubell [100] synthesized PBDs **312a**–**g** and **314a**–**g** (Scheme 58) and examined them by X-ray diffraction since X-ray structural analysis of certain diazepin-2-ones has revealed that their amino acid components adopt dihedral angle values similar to those of the central residue in a γ-turn. The starting point for the synthesis of these PBDs is 1-(2-aminophenyl)pent-4-en-1-one (**307**) which was coupled to Boc-l-α-amino acids **308a**–**g** using *N*,*N*′-dicyclohexylcarbodiimide (DCC), and 4-dimethyl-aminopyridine (DMAP) while the addition of *N*-hydroxybenzotriazole (HOBt) prevented racemisation so that amides **309a**–**g** were obtained, after chromatography, in 67%–97% yields and >93% enantiomeric excess. Two different routes to PBDs **312a**–**g** have been presented. The first route involves removal of the Boc group from ketones **309a**–**g**, cyclocondensation of the formed aminoketones and free-basing of the cyclic imine hydrochlorides, to afford benzodiazepinones **310a**–**g**. Ozonolysis of these compounds and subsequent reduction of the formed ozonide with excess dimethyl sulphide, provided (*S*)-PBDs **312a**–**g**. The second route to these compounds requires oxidative cleavage of olefins **309a**–**g** to provide 1,4-ketoaldehydes **311a**–**g** that underwent microwave irradiation causing cleavage of the Boc group followed by two consecutive cyclocondensations to afford (*S*)-PBDs **312a**–**g**, in slightly better yields than the first method. The 3-methyl analogues of the latter compounds, (*S*)-PBDs **314a**–**g**, were prepared from 1,4-diones **313a**–**g** using the same principle of forming first the diazepine ring followed by the pyrrole ring but under different reaction conditions. Thus, trifluoroacetic acid (TFA) was applied to remove the Boc group of compounds **313a**–**g**, the resulting ammonium trifluoroacetates were free-based using Amberlyst A-21 ion-exchange resin while the non-isolable aminoketones that were produced, underwent double cyclocondensation with *p*-toluenesulfonic acid as catalyst, to provide (*S*)-PBDs **314a**–**g**. Next, the Tsuji-Wacker oxidation was applied to convert the terminal ethene group of compounds **309a**–**g** to the ethanal group of compounds **313a**–**g**. Comparing the two double cyclisations of diketones **311a**–**g** and **313a**–**g** leading to the respective PBDs **312a**–**g** and **314a**–**g**, it seems that the use of microwave irradiation at 150 °C is more efficient without the need to use solvents and reagents.

## 3. Conclusions

The interest in synthesising pyrrolo[1,4]benzodiazepines is mainly due to the ability of pyrrolo[2,1-*c*][1,4]benzodiazepines to interact with DNA. The molecules fit perfectly in the minor groove of DNA because of their right-handed helical conformation adopted from the (*S*)-configuration at C11a and interact with a selectivity for 5′-purine-G-purine sequences forming a covalent bond with guanine residues. The synthesis of these molecules extends from monomeric analogues of the natural products to dimeric adducts and recently to trimeric adducts. The evaluation of the antitumour activity of these compounds is dominated by *in vitro* cytotoxicity against tumour cell lines, DNA thermal denaturation experiments and DNA footprinting assays used to measure the DNA-binding affinity and establish the sequence selectivity. Although pyrrolo[1,2-*a*][1,4]benzodiazepines and pyrrolo[1,2-*d*][1,4]benzodiazepines have been very much less studied, they possess a wide range of useful biological activities.

## References

[1] Leimgruber W., Batcho A.D., Czajkowski R.C. (1968). Total synthesis of anthramycin. J. Am. Chem. Soc..

[2] Cheeseman G.W.H., Rafiq M. (1971). Further cyclisation reactions of 1-arylpyrroles. J. Chem. Soc. C.

[3] Yamawaki Y., Watanabe M., Yamamura S., Saito S. (1977). Studies on synthetic drugs. II. Syntheses of pyrrolo[1,2-*d*][1,4]benzodiazepine derivatives. Yakugaku Zasshi.

[4] Leimgruber W., Stefanovic V., Shenker F., Karr A., Berger J. (1965). Isolation and characterization of anthramycin, a new antitumour antibiotic. J. Am. Chem. Soc..

[5] Thurston D.E., Neidle S., Waring M.J. (1993). Advances in the study of pyrrolo[2,1-*c*][1,4]benzodiazepine (PBD) antitumour antibiotics. Molecular Aspects of Anticancer Drug-DNA Interactions.

[6] Arora S.K. (1979). Structural investigations of mode of action of drugs. II. Molecular structure of anthramycin methyl ether monohydrate. Acta Crystallogr. B.

[7] Hurley L.H., Reck T., Thurston D.E., Langley D.R., Holden K.G., Hertzberg R.P., Hoover J.R.E., Gallagher G., Faucette L.F. (1988). Pyrrolo[1,4]benzodiazepine antitumour antibiotics: Relationship of DNA alkylation and sequence specificity to the biological activity of natural and synthetic compounds. Chem. Res. Toxicol..

[8] Fotso S., Zabriskie T.M., Proteau P.J., Flatt P.M., Santosa D.A., Mahmud T. (2009). Limazepines A–F, pyrrolo[1,4]benzodiazepine antibiotics from an Indonesian *Micrococcus* sp.. J. Nat. Prod..

[9] Nakatani S., Yamamoto Y., Hayashi M., Komiyama K., Ishibashi M. (2004). Cycloanthranilylproline-Derived Constituents from a Myxomycete *Fuligo candida*. Chem. Pharm. Bull..

[10] Hu W.-P., Yu H.-S., Sung P.-J., Tsai F.-Y., Shen Y.-K., Long-Sen Chang L.-S., Wang J.-J. (2007). DC-81-Indole Conjugate Agent Induces Mitochondria Mediated Apoptosis in Human Melanoma A375 Cells. Chem. Res. Toxicol..

[11] Thurston D.E., Bose D.S. (1994). Synthesis of DNA interactive pyrrolo[2,1-*c*][1,4]benzodiazepines. Chem. Rev..

[12] Antonow D., Thurston D.E. (2011). Synthesis of DNA-interactive pyrrolo[2,1-*c*][1,4]benzodiazepines (PBDs). Chem. Rev..

[13] Hartley J.A. (2011). The development of pyrrolobenzodiazepines as antitumour agents. Expert Opin. Investig. Drugs.

[14] Gerratana B. (2012). Biosynthesis, synthesis, and biological activities of pyrrolobenzodiazepines. Med. Res. Rev..

[15] Kaliszczak M., Antonow D., Patel K.I., Howard P., Jodrell D.I., Thurston D.E., Guichard S.M. (2010). Optimization of the antitumor activity of sequence-specific pyrrolobenzodiazepine derivatives based on their affinity for ABC transporters. Am. Assoc. Pharm. Sci. J..

[16] Rahman K.M., Vassoler H., James C.H., Thurston D.E. (2010). DNA sequence preference and adduct orientation of pyrrolo[2,1-*c*][1,4]benzodiazepine antitumor agents. ACS Med. Chem. Lett..

[17] Janjigian Y.Y., Lee W., Kris M.G., Miller V.A., Krug L.M., Azzoli C.G., Senturk E., Calcutt M.W., Rizvi N.A. (2010). A phase I trial of SJG-136 (NSC#694501) in advanced solid tumours. Cancer Chemother. Pharmacol..

[18] Hartley J.A., Hamaguchi1 A., Coffils M., Martin C.R.H., Suggitt M., Chen Z., Gregson S.J., Masterson L.A., Tiberghien A.C., Hartley J.M. (2010). SG2285, a novel C2-aryl-substituted pyrrolobenzodiazepine dimer pro-drug that cross-links DNA and exerts highly potent antitumour activity. Cancer Res..

[19] Reid J.M., Buhrow S.A., Kuffel M.J., Jia L., Spanswick V.J., Hartley J.A., Thurston D.E., Tomaszewski J.E., Ames M.M. (2011). Pharmacokinetics, pharmacodynamics and metabolism of the dimeric pyrrolobenzodiazepine SJG-136 in rats. Cancer Chemother. Pharmacol..

[20] Rosado H., Rahman K.M., Feuerbaum E.-A., Hinds J., Thurston D.E., Taylor P.W. (2011). The minor groove-binding agent ELB-21 forms multiple interstrand and intrastrand covalent cross-links with duplex DNA and displays potent bactericidal activity against methicillin-resistant Staphylococcus aureus. J. Antimicrob. Chemother..

[21] Rahman K.M., James C.H., Bui T.T.T., Drake A.F., Thurston D.E. (2011). Observation of a single-stranded DNA/pyrrolobenzodiazepine adduct. J. Am. Chem. Soc..

[22] Rahman K.M., James C.H., Thurston D.E. (2011). Effect of base sequence on the DNA cross-linking properties of pyrrolobenzodiazepine (PBD) dimers. Nucleic Acids Res..

[23] Deans A.J., West S.C. (2011). DNA interstrand crosslink repair and cancer. Nat. Rev. Cancer.

[24] Rahman K.M., James C.H., Thurston D.E. (2011). Observation of the reversibility of a covalent pyrrolobenzodiazepine (PBD) DNA adduct by HPLC/MS and CD spectroscopy. Org. Biomol. Chem..

[25] Yonemoto I.T., Li W., Khullar A., Reixach N., Gerratana B. (2012). Mutasynthesis of a potent anticancer sibiromycin analogue. ACS Chem. Biol..

[26] Hartley J.A., Hochhauser D. (2012). Small molecule drugs—Optimizing DNA damaging agent-based therapeutics. Curr. Opin. Pharm..

[27] Hartley J.A., Hamaguchi A., Suggitt M., Gregson S.J., Thurston D.E., Howard P.W. (2012). DNA interstrand cross-linking and *in vivo* antitumor activity of the extended pyrrolo[2,1-*c*][1,4]benzodiazepine dimer SG2057. Investig. New Drugs.

[28] Rahman K.M., Rosado H., Moreira1 J.B., Feuerbaum E.-A., Fox K.R., Stecher E., Howard P.W., Gregson S.J., James C.H., de la Fuente M. (2012). Antistaphylococcal activity of DNA-interactive pyrrolobenzodiazepine (PBD) dimers and PBD-biaryl conjugates. J. Antimicrob. Chemother..

[29] Thurston D.E., Vassoler H., Jackson P.J.M., James C.H., Rahman K.M. (2015). Effect of hairpin loop structure on reactivity, sequence preference and adduct orientation of a DNA-interactive pyrrolo[2,1-*c*][1,4]benzodiazepine (PBD) antitumour agent. Org. Biomol. Chem..

[30] Jeffrey S.C., Burke P.J., Lyon R.P., Meyer D.W., Sussman D., Anderson M., Hunter J.H., Leiske C.I., Miyamoto J.B., Nicholas N.D. (2013). A potent anti-CD70 antibody-drug conjugate combining a dimeric pyrrolobenzodiazepine drug with site-specific conjugation technology. Bioconjug. Chem..

[31] Sutherland M.S.K., Walter R.B., Jeffrey S.C., Burke P.J., Yu C., Kostner H., Stone S., Ryan M.C., Sussman D., Lyon R.P. (2013). SGN-CD33A: A novel CD33-targeting antibody–drug conjugate using a pyrrolobenzodiazepine dimer is active in models of drug-resistant AML. Blood.

[32] Flygare J.A., Thomas H., Pillow T.H., Aristoff P. (2013). Antibody-drug Conjugates for the treatment of cancer. Chem. Biol. Drug Des..

[33] Hartley J.A., Neidle S. (2014). Antibody-Drug Conjugates Delivering DNA Cytotoxics. Cancer Drug Design and Discovery.

[34] Rahman K.M., Corcoran D.B., Bui T.T.T., Jackson P.J.M., David E., Thurston D.E. (2014). Pyrrolobenzodiazepines (PBDs) do not bind to DNA G-quadruplexes. PLoS ONE.

[35] Schneditz G., Rentner J., Roier S., Pletz J., Herzog K.A.T., Troeger R.B.H., Schild S., Weber H., Breinbauer R., Gorkiewicz G. (2014). Enterotoxicity of a nonribosomal peptide causes antibiotic-associated colitis. Proc. Natl. Acad. Sci. USA.

[36] Rettig M., Langel W., Kamal A., Weisz K. (2010). NMR structural studies on the covalent DNA binding of a pyrrolobenzodiazepine–naphthalimide conjugate. Org. Biomol. Chem..

[37] Hopton S.R., Thompson A.S. (2011). Nuclear Magnetic Resonance Solution Structures of Inter- and Intrastrand Adducts of DNA Cross-Linker SJG-136. Biochemistry.

[38] Raju G., Srinivas R., Reddy V.S., Idris M.M., Kamal A., Nagesh N. (2012). Interaction of pyrrolobenzodiazepine (PBD) ligands with parallel intermolecular G-quadruplex complex using spectroscopy and ESI-MS. PLoS ONE.

[39] Jackson P.J.M., James C.H., Jenkins T.C., Rahman K.M., David E., Thurston D.E. (2014). Computational studies support the role of the C7-sibirosamine sugar of the pyrrolobenzodiazepine (PBD) sibiromycin in transcription factor inhibition. ACS Chem. Biol..

[40] Oh M., Jang J.-H., Choo S.-J., Kim S.-O., Kim J.W., Ko S.-K., Soung N.-K., Lee J.-S., Kim C.J., Oh H. (2014). Boseongazepines A–C, pyrrolobenzodiazepine derivatives from a *Streptomyces* sp. 11A057. Bioorg. Med. Chem. Lett..

[41] Corelli F., Massa S., Stefancich G., Ortenzi G., Artico M., Pantaleoni G.C., Palumbo G., Fanini D., Giorgi R. (1986). Benzodiazepines with Both Sedative and Analgesic Activities. Eur. J. Med. Chem.

[42] Mai A., di Santo R., Massa S., Artico M., Pantaleoni G.C., Giorgi R., Coppolino M.F., Barracchini A. (1995). Pyrrolobenzodiazepines with antinociceptive activity: Synthesis and pharmacological activities. Eur. J. Med. Chem..

[43] Massa S., Artico M., Mai A., Corelli F., Pantaleoni G.C., Giorgi R., Ottaviani D., Cagnotto A. (1990). Pyrrolobenzodiazepine and related systems. I. Synthesis and pharmacological evaluation of new 5,6-dihydro-4*H*-pyrrolo[1,2-*a*][1,4]benzodiazepine derivatives. Farmaco.

[44] Meerpoel L., van Gestel J., van Gerven F., Woestenborghs F., Marichal P., Sipido V., Terence G., Nash R., Corens D., Richards R.D. (2005). Pyrrolo[1,2-*a*][1,4]benzodiazepine: A novel class of non-azole anti-dermatophyte anti-fungal agents. Bioorg. Med. Chem. Lett..

[45] Hara T., Kayama Y., Mori T., Itoh K., Fujimori H., Sunami T., Hashimoto Y., Ishimoto S. (1978). Diazepines. 5. Synthesis and Biological Action of 6-Phenyl-4*H*-pyrrolo[1,2-*a*][1,4]benzodiazepines. J. Med. Chem..

[46] Massa S., Corelli F., Artico M., Mai A., Silvestri R., Pantaleoni G.C., Palumbo G., Fanini D., Giorgi R. (1989). 5-Aroyl-5,6-dihydro-4*H*-pyrrolo[1,2-*a*][1,4]benzodiazepin-4-carboxylic acids: Synthesis and analgesic and neurobehavioral activity. Farmaco.

[47] Massa S., Artico M., Mai A., Corelli F., Botta M., Tafi A., Pantaleoni G.C., Giorgi R., Coppolino M.F., Cagnotto A. (1992). Pyrrolobenzodiazepines and related systems. 2. Synthesis and biological properties of isonoraptazepine derivatives. J. Med. Chem..

[48] De Lucca G.V., Otto M.J. (1992). Synthesis and anti-HIV activity of pyrrolo[1,2-*d*][1,4]benzodiazepine-6-ones. Bioorg. Med. Chem. Lett..

[49] Kamal A., Rajender, Reddy R., Reddy M.K., Balakishan G., Shaik T.B., Chourasia M., Sastry G.N. (2009). Remarkable enhancement in the DNA-binding ability of C2-fluoro substituted pyrrolo[2,1-c]-[1,4]benzodiazepines and their anticancer potential. Bioorg. Med. Chem..

[50] Kamal A., Reddy M.K., Ramaiah M.J., Reddy R.J.S., Srikanth Y.V.V., Dastagiri D., Bharathi E.V., Pushpavalli S.N., Sarma P., Pal-Bhadra M. (2011). Synthesis and biological evaluation of estradiol linked pyrrolo[2,1-*c*][1,4]benzodiazepine (PBD) conjugates as potential anticancer agents. Bioorg. Med. Chem..

[51] Kamal A., Reddy M.K., Ramaiah M.J., Srikanth Y.V.V., Reddy R.V.S., Kumar G.B., Pushpavalli S.N., Bag I., Juvekar A., Sen S. (2011). Synthesis of aryl-substituted naphthalene-linked pyrrolobenzodiazepine conjugates as potential anticancer agents with apoptosis-inducing ability. ChemMedChem.

[52] Kamal A., Prabhakar S., Ramaiah M.J., Reddy P.V., Reddy C.R., Mallareddy A., Shankaraiah N., Reddy T.L.M., Pushpavalli S.N., Pal-Bhadra M. (2011). Synthesis and anticancer activity of chalcone-pyrrolobenzodiazepine conjugates linked via 1,2,3-triazole ring side-armed with alkane spacers. Eur. J. Med. Chem..

[53] Kamal A., Shetti R.V., Ramaiah M.J., Swapna P., Reddy K.S., Mallareddy A., Rao M.P.N., Chourasia M., Sastry G.N., Juvekar A. (2011). Carbazole-pyrrolo[2,1-*c*][1,4]benzodiazepine conjugates: Design, synthesis, and biological evaluation. Med. Chem. Commun..

[54] Thurston D.E., Murty V.S., Langley D.R., Jones G.B. (1990). *O*-Debenzylation of a pyrrolo[2,1-*c*][1,4]benzodiazepine in the presence of a carbinolamine functionality: Synthesis of DC-81. Synthesis.

[55] Kamal A., Ramakrishna G., Nayak V.L., Raju P., Rao A.V.S., Viswanath A., Vishnuvardhan M.V.P.S., Ramakrishna S., Srinivas G. (2012). Design and synthesis of benzo[c,d]indolone-pyrrolobenzodiazepine conjugates as potential anticancer agents. Bioorg. Med. Chem..

[56] Kamal A., Srikanth Y.V.V., Ramaiah M.J., Khan M.N.A., Reddy M.K., Ashraf Md., Lavanya A., Pushpavalli S.N., Pal-Bhadra M. (2012). Synthesis, anticancer activity and apoptosis inducing ability of bisindole linked pyrrolo[2,1-*c*][1,4]benzodiazepine conjugates. Bioorg. Med. Chem. Lett..

[57] Kamal A., Ramakrishna G., Ramaiah M.J., Viswanath A., Ayinampudi V.S.R., Bagul C., Mukhopadyay D., Pushpavalli S.N., Pal-Bhadra M. (2013). Design, synthesis and biological evaluation of imidazo[1,5-*a*]pyridine–PBD conjugates as potential DNA-directed alkylating agents. Med. Chem. Commun..

[58] Kamal A., Sreekanth K., Shankaraiah N., Sathish M., Nekkanti S., Srinivasulu V. (2015). Dithiocarbamate/piperazine bridged pyrrolobenzodiazepines as DNA-minor groove binders: Synthesis, DNA-binding affinity and cytotoxic activity. Bioorg. Chem..

[59] Bose D.S., Idrees M., Todewale I.K., Jakka N.M., Rao J.V. (2012). Hybrids of privileged structures benzothiazoles and pyrrolo[2,1-*c*][1,4]benzodiazepin-5-one, and diversity-oriented synthesis of benzothiazoles. Eur. J. Med. Chem..

[60] Langley D.R., Thurston D.E. (1987). A versatile and efficient synthesis of carbinolamine-containing pyrrolo[1,4]benzodiazepines via the cyclisation of *N*-(2-aminobenzoyl)-pyrrolidine-2-carboxaldehyde diethyl thioacetals: total synthesis of prothracarcin. J. Org. Chem..

[61] Wells G., Martin C.R.H., Howard P.W., Sands Z.A., Laughton C.A., Tiberghien A., Woo C.K., Masterson L.A., Stephenson M.J., Hartley J.A. (2006). Design, synthesis, and biophysical and biological evaluation of a series of pyrrolobenzodiazepine-poly(*N*-methylpyrrole) conjugates. J. Med. Chem..

[62] Kotecha M., Kluza J., Wells G., O’Hare C.C., Forni C., Mantovani R., Howard P.W., Morris P., Thurston D.E., Hartley J.A. (2008). Inhibition of DNA binding of the NF-Y transcription factor by the pyrrolobenzodiazepine-polyamide conjugate GWL-78. Mol. Cancer Ther..

[63] Brucoli F., Hawkins R.M., James C.H., Wells G., Jenkins T.C., Ellis T., Hartley J.A., Howard P.W., Thurston D.E. (2011). Novel C8-linked pyrrolobenzodiazepine (PBD)–heterocycle conjugates that recognize DNA sequences containing an inverted CCAAT box. Bioorg. Med. Chem. Lett..

[64] Rahman K.M., Jackson P.J.M., James C.H., Basu B.P., Hartley J.A., de la Fuente M., Schatzlein A., Robson M., Pedley R.B., Pepper C. (2013). GC-targeted C8-linked pyrrolobenzodiazepine-biaryl conjugates with femtomolar *in vitro* cytotoxicity and *in vivo* antitumor activity in mouse models. J. Med. Chem..

[65] Brucoli F., Hawkins R.M., James C.H., Jackson P.J.M., Wells G., Jenkins T.C., Ellis T., Kotecha M., Hochhauser D., Hartley J.A. (2013). An extended pyrrolobenzodiazepine–polyamide conjugate with selectivity for a DNA sequence containing the ICB2 transcription factor binding site. J. Med. Chem..

[66] Kolakowski R.V., Young T.D., Howard P.W., Jeffrey S.C., Senter P.D. (2015). Synthesis of a C2-arylpyrrolo[2,1-*c*][1,4]benzodiazepine monomer enabling the convergent construction of symmetrical and non-symmetrical dimeric analogs. Tetrahedron Lett..

[67] Kamal A., Prabhakar S., Shankaraiah N., Markandey N., Reddy P.V., Srinivasulu V., Sathish M. (2013). AlCl_3_–NaI assisted cleavage of polymer-bound esters with concomitant amine coupling and azido-reductive cyclisation: Synthesis of pyrrolobenzodiazepine derivatives. Tetrahedron Lett..

[68] Kamal A., Shankaraiah N., Devaiah V., Reddy K.L. (2006). Solid-phase synthesis of fused [2,1-*b*]-quinazolinone alkaloids. Tetrahedron Lett..

[69] Hemming K., Chambers C.S., Jamshaid F., O’Gorman P.A. (2014). Intramolecular azide to alkene cycloadditions for the construction of pyrrolobenzodiazepines and azetidinobenzodiazepines. Molecules.

[70] Hemming K., Chambers C.S., Hamasharif M.S., João H., Khan M.N., Nilesh Patel N., Airley R., Day S. (2014). Azide based routes to tetrazolo and oxadiazolo derivatives of pyrrolobenzodiazepines and pyrrolobenzothiadiazepines. Tetrahedron.

[71] Araújo A.C., Rauter A.P., Nicotra F., Airoldi C., Costa B., Cipolla L. (2011). Sugar-based enantiomeric and conformationally constrained pyrrolo[2,1-*c*][1,4]benzodiazepines as potential GABAA ligands. J. Med. Chem..

[72] Cipolla L., Redaelli C., Nicotra F. (2005). Synthesis of a spiro d-proline analogue bearing d-fructose. Lett. Drug Des. Discov..

[73] Legerén L., Domínguez D. (2010). Synthesis of 5-arylpyrrolo[2,1-*c*][1,4]benzodiazepines under mild cyclodehydration conditions. Tetrahedron.

[74] Sharma S.K., Mandadapu A.K., Kumaresan K., Arora A., Gauniyal H.M., Kundu B. (2010). Efficient synthesis of naturally occurring skeleton 5–7–6 tricyclic pyrrolo[2,1-*c*][1,4]benzodiazepin-5-one and its derivatives via cationic π-cyclisation. Synthesis.

[75] Leber J.D., Hoover J.R.E., Holden K.G., Johnson R.K., Hecht S.M. (1988). A revised structure of sibiromycin. J. Am. Chem. Soc..

[76] Gao K., Wu B., Yu C.-B., Chen Q.-A., Ye Z.-S., Zhou Y.G. (2012). Iridium catalyzed asymmetric hydrogenation of cyclic imines of benzodiazepinones and benzodiazepines. Org. Lett..

[77] Molinari A.J., Trybulski E.J., Bagli J., Croce S., Considine J., Qi J., Ali K., Demaio W., Lihotz L., Cochran D. (2007). Identification and synthesis of major metabolites of Vasopressin V_2_-receptor agonist WAY-151932, and antagonist, Lixivaptan^®^. Bioorg. Med. Chem. Lett..

[78] Roszkowski P., Maurin J.K., Czarnocki Z. (2012). First enantioselective synthesis of aptazepine. Synthesis.

[79] Pardo L.M., Tellitu I., Esther Domínguez E. (2010). A versatile PIFA-mediated approach to structurally diverse pyrrolo(benzo)diazepines from linear alkynylamides. Tetrahedron.

[80] Raines S., Chai S.Y., Palopoli F.P. (1976). Mannich reactions. Synthesis of 4,5-dihydropyrrolo[l,2-*a*]quinoxalines, 2,3,4,5-tetrahydro-l*H*-pyrrolo[l,2-*a*][l,4]diazepines and 5,6-dihydro-4*H*-pyrrolo[1,2-*a*][1,4]benzodiazepines. J. Heterocycl. Chem..

[81] Cheeseman G.W.H., Greenberg S.G. (1979). Synthesis of 5,6-dihydro-4*H*-pyrrolo[1,2-*a*][1,4]benzodiazepines. J. Heterocycl. Chem..

[82] Massa S., Mai A., di Santo R., Artico M. (1993). 5,6-Dihydro-4*H*-pyrrolo[1,2-*a*][1,4]benzodiazepine-4,4-diacetic acid diethyl ester, an useful synthon for the synthesis of new polycyclic nitrogen systems of pharmacological interest. J. Heterocycl. Chem..

[83] Massa S., Mai A., Artico M., Corelli F., Botta M. (1989). Synthesis of 3b,4,6,7-tetrahydro-5*H*,9*H*-pyrazino[2,l-*c*]-pyrrolo[1,2-*a*][l,4]benzodiazepine, a valuable precursor of potential central nervous system agents. Tetrahedron.

[84] Massa S., di Santo R., Costi R., Artico M. (1993). Research on nitrogen containing heterocyclic compounds. XX. Synthesis of 8*H*-Imidazo[5,1-*c*]pyrrolo[1,2-*a*][1,4]benzodiazepine and its 6-derivatives. J. Heterocycl. Chem..

[85] Voskressensky L.G., Borisova T.N., Babakhanova M.I., Titov A.A., Chervyakova T.M., Novikov R.A., Butin A.S., Khrustalev V.N., Varlamov A.V. (2014). Transformation of 4-substituted tetrahydropyrrolo-benzodiazepines in a three-component reaction with methyl propiolate and indole. Chem. Heterocycl. Compd..

[86] Voskressensky L.G., Borisova T.N., Babakhanova M.I., Chervyakova T.M., Titov A.A., Butin A.S., Nevolina T.A., Khrustalev V.N., Varlamov A.V. (2013). Synthesis of pyrrolo[1,2-*a*][1,6]benzodiazonines from pyrrolo[1,2-*a*][1,4]benzodiazepines and alkynes containing electron-acceptor substituents. Chem. Heterocycl. Compd..

[87] Duceppe J.S., Gauthier J. (1984). Synthesis of 5*H*-imidazo[2,1-*c*]pyrrolo[1,2-*a*][1,4]benzodiazepine. J. Heterocycl. Chem..

[88] Kayama Y., Hara T., Itoh K., Sunami T. (1977). Diazepines II. A new synthesis of 4*H*-pyrrolo[1,2-*a*][1,4]-benzodiazepine. J. Heterocycl. Chem..

[89] Korakas D., Varvounis G. (1994). A convenient synthesis of 2-aminomethyl-1-arylpyrroles. Synthesis.

[90] Korakas D., Varvounis G. (1994). Synthesis of 5,6-dihydro-4*H*-pyrrolo[1,2-*a*][1,4]benzodiazepine and 10,11-dihydro-5*H*,12*H*-pyrrolo[2,1-*c*][1,4]benzodiazocine derivatives *via* cyclisation of 2-aminomethylpyrroles. J. Heterocycl. Chem..

[91] Butin A.V., Nevolina T.A., Shcherbinin V.A., Trushkov I.V., Cheshkov D.A., Krapivin G.D. (2010). Furan ring opening-pyrrole ring closure: A new synthetic route to aryl(heteroaryl)-annulated pyrrolo[1,2-*a*][1,4]diazepines. Org. Biomol. Chem..

[92] Tempest P., Ma V., Kelly M.G., Jones W., Hulme C. (2001). MCC/S_N_Ar methodology. Part 1: Novel access to a range of heterocyclic cores. Tetrahedron Lett..

[93] Mucedda M., Muroni D., Saba A., Manassero C. (2007). Concise diastereospecific pyrrolo[1,2-*a*][1,4]-benzodiazepinone synthesis. Tetrahedron.

[94] Muroni D., Mucedda M., Saba A. (2009). Efficient synthesis of 1-azatricyclic ring systems from anthranylamide. Heterocycles.

[95] Wang H., Jiang Y., Gao K., Ma D. (2009). Facile synthesis of 1,4-benzodiazepin-3-ones from *o*-bromobenzylamines and amino acids via a cascade coupling/condensation process. Tetrahedron.

[96] Kraus G.A., Yue S. (1983). Amidoalkylation Reactions of Anilines. A direct synthesis of benzodiazepines. J. Org. Chem..

[97] Aiello E., Dattolo G., Cirrincione G., Plescia S.E., Daidone G. (1979). Polycondensed nitrogen heterocycles. VII. 5,6-Dihydro-7*H*-pyrrolo[1,2-*d*][1,4]benzodiazepin-6-ones. A novel series of annelated 1,4-benzodiazepines. J. Heterocycl. Chem..

[98] Dattolo G., Cirrincione G., Aiello E. (1980). Polycondensed nitrogen heterocycles. IX. 5,6-Dihydro-7*H*-pyrrolo[1,2-*d*][1,4]benzodiazepin-6-one. J. Heterocycl. Chem..

[99] Nevolina T.A., Shcherbinin V.A., Serdyuk O.V., Butin A.V. (2011). Furan ring opening-pyrrole ring closure: A new route to pyrrolo[1,2-*d*][1,4]benzodiazepin-6-ones. Synthesis.

[100] Dörr A.A., Lubell W.D. (2015). γ-Turn mimicry with benzodiazepinones and pyrrolobenzodiazepinones synthesized from a common amino ketone intermediate. Org. Lett..

